# MixWILD: A program for examining the effects of variance and slope of time-varying variables in intensive longitudinal data

**DOI:** 10.3758/s13428-019-01322-1

**Published:** 2020-01-02

**Authors:** Eldin Dzubur, Aditya Ponnada, Rachel Nordgren, Chih-Hsiang Yang, Stephen Intille, Genevieve Dunton, Donald Hedeker

**Affiliations:** 1 University of Southern California, Los Angeles, CA USA; 2 Northeastern University, Boston, MA USA; 3 University of Illinois - Chicago, Chicago, IL USA; 4 University of Chicago, Chicago, IL USA

**Keywords:** Intensive longitudinal data, Ecological momentary assessment, Multilevel, Mixed models, Heteroscedasticity, Variance modeling

## Abstract

The use of intensive sampling methods, such as ecological momentary assessment (EMA), is increasingly prominent in medical research. However, inferences from such data are often limited to the subject-specific mean of the outcome and between-subject variance (i.e., random intercept), despite the capability to examine within-subject variance (i.e., random scale) and associations between covariates and subject-specific mean (i.e., random slope). MixWILD (Mixed model analysis With Intensive Longitudinal Data) is statistical software that tests the effects of subject-level parameters (variance and slope) of time-varying variables, specifically in the context of studies using intensive sampling methods, such as ecological momentary assessment. MixWILD combines estimation of a stage 1 mixed-effects location-scale (MELS) model, including estimation of the subject-specific random effects, with a subsequent stage 2 linear or binary/ordinal logistic regression in which values sampled from each subject’s random effect distributions can be used as regressors (and then the results are aggregated across replications). Computations within MixWILD were written in FORTRAN and use maximum likelihood estimation, utilizing both the expectation-maximization (EM) algorithm and a Newton–Raphson solution. The mean and variance of each individual’s random effects used in the sampling are estimated using empirical Bayes equations. This manuscript details the underlying procedures and provides examples illustrating standalone usage and features of MixWILD and its GUI. MixWILD is generalizable to a variety of data collection strategies (i.e., EMA, sensors) as a robust and reproducible method to test predictors of variability in level 1 outcomes and the associations between subject-level parameters (variances and slopes) and level 2 outcomes.

**Electronic supplementary material:**

The online version of this article (10.3758/s13428-019-01322-1) contains supplementary material, which is available to authorized users.

## Introduction

Mixed-effects regression models (aka hierarchical linear models or multilevel models) have become a popular method for analysis of longitudinal and clustered (Goldstein, 2011; Raudenbush & Bryk, 2002) data. These models include both fixed effects (standard regression coefficients) and random effects (terms representing between-subject heterogeneity). The random location effects, defined as the degree to which a subject deviates from the population mean, are used to account for the non-independence of observations within subjects (i.e., clusters)—observations from the same subject will be more similar than observations from different subjects. Although the language and examples in this manuscript apply to longitudinal data, the same models can be used for observations within clusters such as families, classrooms, and clinics.

In this setting, we are particularly interested in examining if the characteristics of time-varying data shown by subjects (both overall average as well as degree of consistency) during a longitudinal study can predict other, potentially future, subject-level characteristics. For example, does the average level of a subject’s positive mood and the amount of fluctuations around that level predict whether a subject is obese, or how much time a subject is sedentary. We might theorize that subjects showing a lot of fluctuations in mood would be less likely to be consistently exercising, and more likely to be obese.

Typically, the intra-individual variability (i.e., error variance), or the within-subjects (WS) variance, and the variance of the random effects, or the between-subjects (BS) variance, are treated as being homogeneous across subject groups or levels of covariates. However, this may not be the case, and assumptions of homogeneity of variance can be relaxed by modeling differences in variances, both between and within subjects. The study of intra-individual variability has received increased attention (Fleeson, 2004; Hertzog & Nesselroade, 2003; Martin & Hofer, 2004; Nesselroade, 2004); these articles describe many of the conceptual issues and some traditional statistical approaches for examining such variation.

Modern data collection procedures (i.e., ecological momentary assessments (EMAs) (Bolger et al., 2003; Stone et al., 1999; Stone & Shiffman, 1994; Dimotakis et al., 2013; Feldman & Barrett, 2001; Larson & Csikszentmihalyi, 1983; Scollon et al., 2003)) allow for collection of much richer datasets (sometimes referred to as intensive longitudinal data (ILD) (Walls & Schafer, 2006)) than standard longitudinal studies. As a result of repeated measurements per day over the course of a study, EMA procedures allow for more flexibility in modeling. In particular, the mixed-effects location-scale (MELS) model (Hedeker et al., 2008) extends the usual mixed-effects regression model by allowing modeling of both the BS and WS variances in terms of covariates, in addition to the usual modeling of the mean in terms of covariates. Specifically, log-linear sub-models for the BS and WS variances are specified, allowing covariates to influence both types of variance. Additionally, a random subject (scale) effect is added to the WS variance specification, allowing the WS variance to be subject-specific, as well as influenced by covariates. Thus, MELS models include both random subject location and scale effects, which are estimated using empirical Bayes methods (Bock, 1989). These subject-specific estimates indicate a baseline mean level (random intercept), the effect of a covariate on the mean (random slope), and the degree of within-subject variability (random scale). In some cases, it may be of interest to examine whether these subject-estimated summaries of the EMA data are related to other subject-level outcomes. In **MixWILD**, the ability to create a variety of stage 1 MELS models is combined with a stage 2 linear or binary/ordinal (logistic) regression using the subject random effects estimates from the stage 1 MELS model to predict subject-level outcomes.

This manuscript describes the use of the software program **MixWILD**, which allows estimation of a stage 1 MELS model including random subject location and scale effects. These random subject effects can be used as predictors of a subject-level outcome that could be continuous (linear regression) or binary/ordinal (logistic regression) in stage 2 of the joint model. Additional subject-level predictors/covariates can be included, and these can also interact with the stage 1 random effects in predicting the stage 2 subject-level outcome.

Since the random subject effects are estimates, we used the plausible value methodology to repeatedly impute the random effects in the stage 2 analysis (Mislevy, 1991). This approach accounts for the uncertainty in the random effect estimates. The stage 2 analyses are repeated for each set of imputed random effect estimates, and then averaged (using Rubin’s rules for multiple imputation) to yield overall regression estimates. Thus, the full model is estimated in three separate steps:

1. A stage 1 MELS model is estimated (“Stage 1: Mixed-effects location scale model”), and subject-specific random effect estimates and variances are produced.
2. Datasets of imputed subject-specific random effects are created.
3. The stage 2 linear or binary/ordinal regression model is estimated (“Stage 2: linear or logistic regression using stage 1 estimates”) for each of the imputed datasets, and averaged estimates are obtained.

Currently, there is only limited statistical software available for conducting two-stage modeling of the aggregated effects of intensively time-varying outcomes (stage 1) on higher-level outcomes (stage 2); therefore, MixWILD will enhance the toolkit for data analysts faced with understanding ILD data. One can estimate such models using SAS PROC NLMIXED and/or Bayesian software programs (e.g., WinBUGS, JAGS, or Stan). However, SAS PROC NLMIXED requires familiarity with syntax and yet cannot test random intercepts and slopes as predictors, mediators, and moderators of outcome variables. On the other hand, Bayesian programs require advanced programming skills and are not specifically designed for applied researchers. Also, our two-stage modeling approach differs in important ways from other approaches of modeling intra-individual variability. For example, others have proposed calculating summary statistics of variability for each person, such as subject-level standard deviations (SD), mean square of successive differences (MSSD), and probability of acute change (PAC; (Solhan et al., 2009)). By computing such summary statistics separately for each subject, these strategies ignore the fact that subjects can vary quite dramatically in terms of the number of observations that they contribute to the analyses. In other words, these approaches treat each summary statistic as if it was equally precise in its estimation across subjects, which is not the case. Our approach recognizes that subjects can vary in terms of their numbers of observations. Furthermore, previous approaches often then use these summary statistics (SD, MSSD, PAC) in subsequent analyses as fixed quantities, which ignore the fact that they are only estimates with varying degrees of precision. As a result, by treating these as fixed and ignoring this source of variation, the standard errors are too small, leading to more false positive results. Instead, in our stage 2 modeling, we use the plausible values re-sampling approach (Mislevy, 1991) to take into account the variability that is inherent in these estimates. Finally, in our stage 1 model, we can characterize a person’s data in terms of means, slopes, and variances, but additionally control for other covariates in the model. Thus, our subject-level variance estimates can adjust for mean levels and trends across time, for example, which is not possible in previously used summary statistic calculations.

The organization of the manuscript is as follows: “Stage 1: Mixed-effects location scale model” describes the stage 1 MELS model, Section “Stage 2: linear or logistic regression using stage 1 estimates” describes the stage 2 regression models, Section “MixWILD Software Overview” provides screenshots and detailed instructions on using **MixWILD**, as well as explanation of the output. A simulated intensive longitudinal dataset incorporating EMA, in which subjects were measured up to eight times each day during a 7-day measurement period, is used to demonstrate applied examples in “Applied examples”. Section “Conclusion and future work” discusses and summarizes the program.

## Stage 1: Mixed-effects location scale model

**MixWILD** allows a wide variety of models in stage 1 depending on the options chosen. Beginning with a random intercept model (2.0.1), we consider that model as well as two possible extensions of that model.

For measurement *y* of subject *i* (*i* = 1,2,…,*N* subjects) on occasion *j* (*j* = 1,2,…,*n*_*i*_ occasions):

2.0.1 $$ y_{ij} = \boldsymbol{x}_{ij}^{\top} \boldsymbol{\beta} + {\upsilon}_{i} + \epsilon_{ij} , \epsilon_{ij} \sim N(0, \sigma^{2}_{\epsilon}),   {\upsilon}_{i} \sim N(0, \sigma^{2}_{\upsilon}), $$

In Eq. 2.0.1 and subsequent equations, ***x***_*ij*_ is the vector of regressors for the mean (typically including a “1” for the intercept as the first element) and ***β*** is the corresponding vector of regression coefficients. The regressors can either be at the subject level, vary across occasions, or be interactions of subject-level and occasion-level variables.

A traditional multilevel model may be used if covariates are not expected to predict WS variance. For instance, a researcher may be interested in the effects of an individual’s perception of safety on his or her positive affect. Thus, the random intercept represents the between-subject variability of affect (i.e., deviation from the overall mean), and the researcher may be interested in whether perceived safety is associated with subject-specific means (i.e., does perceived safety predict positive affect?) and the between-subject variance (i.e., does perceived safety predict how an individual’s mean positive affect differs from the overall mean?).

Since the modeling of individual-level variation is of particular interest, we can further extend the models to allow covariates to influence the magnitude of the error variance, and even further allow each subject to have their own amount of WS variance, above and beyond the effects of covariates.

In the following sections, we give more explanation about those two extended models: a mixed-effects location scale (MELS) model with the option to model BS variance in terms of covariates, and a mixed-effects multiple location scale (MEMLS) model.

**Fig. 1:**
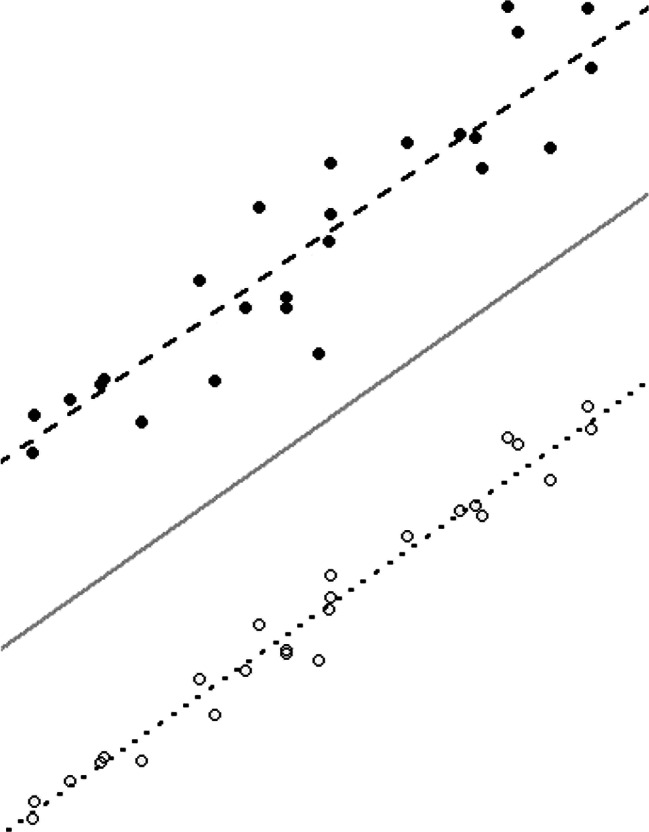
A visual representation of the mixed-effects location scale model

When choosing a model, if subjects are only expected to vary in their intercept and a researcher is interested is in modeling the effect of various covariates on the WS and BS variance, then the MELS model should be used (see Fig. 1 for a visual example). Extending the prior example examining positive affect and perceived safety, the random scale (i.e., WS variance) in the model would be the extent to which a subject’s positive affect deviates from their own mean positive affect. Thus, a researcher would be additionally interested in whether perceived safety predicts the amount an individual deviates from his or her typical level of positive affect.

An extension of this model in Eq. 2.0.1 is to allow modeling of the variance of the random intercept with covariates, rather than requiring it to be constant across all subjects (2.0.2). As examples, we would expect more subject heterogeneity in a disease population than in a healthy one, or we might expect to see more subject heterogeneity as subjects grew older. This model will be extended to allow modeling of the WS variance and expanded on in “MELS model”.

2.0.2 $$ \begin{array}{@{}rcl@{}} y_{ij} &=& \boldsymbol{x}_{ij}^{\top} \boldsymbol{\beta} + {\upsilon}_{i} + \epsilon_{ij} , \epsilon_{ij} \sim N(0, \sigma^{2}_{\epsilon}),\\ {\upsilon}_{i} &\sim& N(0, \sigma^{2}_{{\upsilon}_{i}}), \sigma_{{\upsilon}_{i}}^{2}  =  \exp(\boldsymbol{u}_{i}^{\top} \boldsymbol{\alpha}), \end{array} $$

In Eq. 2.0.2, ***u***_*ij*_ is a vector of regressors (typically including a “1” for the intercept as the first element) and ***α*** is the corresponding vector of coefficients. The regressors can either be at the subject level, vary across occasions, or be interactions of subject-level and occasion-level variables.

**Fig. 2:**
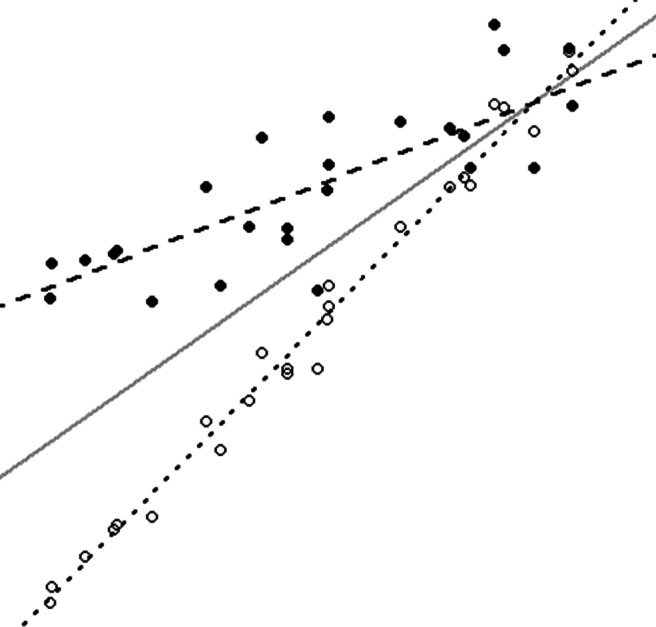
A visual representation of the mixed-effects location scale model

If instead subjects are expected to vary not just in their intercept, but also in their responses to a time-varying covariate, having a slope random effect will be advantageous and the MEMLS model should be used (see Fig. 2 for a visual example). Further extending the prior example, the relationship between perceived safety and positive affect could be identified as the random slope. Hence, a researcher would also be interested in whether differences in positive affect occur as a result of change in perceived safety.

A different extension of Eq. 2.0.1 is to allow a random slope or other random effect in the mean modeling (2.0.3). An example would be if we wanted to allow subjects to not only have their own mean, but also differing trends over time. This model will be extended to allow modeling of the WS variance and expanded on in “Mixed-effects multiple location scale (MEMLS) model”.

2.0.3 $$ y_{ij} = \boldsymbol{x}_{ij}^{\top} \boldsymbol{\beta} + \boldsymbol{z}_{ij}^{\top}\boldsymbol{\upsilon}_{i} + \epsilon_{ij} , \epsilon_{ij} \sim N(0, \sigma^{2}),  \boldsymbol{\upsilon}_{i} \sim N(\mathbf{0}, \boldsymbol{\Sigma}_{\upsilon}), $$

In Eq. 2.0.3, ***z***_*ij*_ is a vector of occasion-level regressors (typically including a “1” for the intercept as the first element) and ***υ***_*i*_ is the vector of random location effects for subject *i*. These random location subject effects allow subject-specific differentiation in the response to occasion-level regressors.

For all three models, samples of the each subject’s random effect values can then be used as predictors in a stage 2 model, if desired.

### MELS model

The mixed-effects location scale model and the corresponding program have been well-explained in Hedeker et al., (2008). Visually, Fig. 1 shows a simple example of the model. The average across all subjects is depicted by the solid line, and the lines of two subjects are shown as dotted and dashed lines. Here, the average solid line has the same slope as each subject. In general, there will be a line for each subject in the dataset, but only two are shown here for simplicity. In this random intercept model, each subject’s line is parallel to the averaged line based on their covariate values. The subject shown with a dashed line has a greater random intercept (location), while the dotted line has a lower random intercept (location). A subject’s random location effect (i.e., the amount that a subject deviates from the mean) is designated by *υ*_*i*_. In the figure, this is represented by the distance between lines–positive for the dashed line and negative for the dotted line. The amount of spread across the lines indicates the BS variance–if the lines are close together then subjects are more similar (smaller variance) and vice versa. How much variation the individual points have relative to each subject’s line indicates the WS variance. In the figure, the subject with open circles has a low WS variance, while the subject with filled circles has a larger WS variance. The WS variance is modeled in terms of covariates as well as a random subject (scale) effect *ω*_*i*_. Thus, the consistency/erraticism of a subject may be explained by covariates, as well as a unique individual contribution.

In terms of the statistical model, the measurement *y* of subject *i* (*i* = 1,2,…,*N* subjects) on occasion *j* (*j* = 1,2,…,*n*_*i*_ occasions) is modeled as follows:

2.1.1 $$ y_{ij} = \boldsymbol{x}_{ij}^{\top} \boldsymbol{\beta} + {\upsilon}_{ij} + \epsilon_{ij} ,\epsilon_{ij} \sim N(0, \sigma^{2}_{\epsilon_{ij}}), {\upsilon}_{ij} \sim N(0, \sigma^{2}_{{\upsilon}_{ij}}), $$

where

2.1.2 $$ \sigma_{{\upsilon}_{ij}}^{2}  =  \exp(\boldsymbol{u}_{ij}^{\top} \boldsymbol{\alpha}), $$

and

2.1.3 $$ \sigma_{\epsilon_{ij}}^{2}  =  \exp(\boldsymbol{w}_{ij}^{\top} \boldsymbol{\tau} + \tau_{l} {\upsilon}_{i}+ \tau_{q} {\upsilon^{2}_{i}} + \omega_{i}),   \omega_{i}\sim N(0,\sigma_{\omega}^{2}).  $$

In Eq. 2.1.3, ***w***_*ij*_ is a vector of regressors for the WS variance (typically including a “1” for the intercept as the first element) and ***τ*** is the corresponding vector of regression coefficients. These could be the same or different variables as in ***x***_*ij*_, and can be at the subject level, vary across occasions, or be interactions of subject-level and occasion-level variables.

Also in Eq. 2.1.3, the random scale effect (*ω*_*i*_) allows the WS variance to vary across subjects beyond the contribution of covariates. Similar to the random location effect in Eq. 2.0.1, the covariates entered in a model may not account for all of the reasons that subjects differ from each other.

The variances are subscripted by *i* and *j* to indicate that their values change depending on the values of the covariates *u*_*ij*_ and *w*_*ij*_ (and their coefficients). The number of parameters associated with these variances does not vary with *i* or *j*. The exponential function is used to ensure that the resulting variances are strictly positive. Note that although we have used different letters to represent the covariates in the different models, there is no restriction and the same covariates could be used.

The model also allows the random intercept (the random location effect *υ*_*i*_) to influence the WS variance. A quadratic relationship could be useful for rating scale data with ceiling and/or floor effects, where subjects that have mean levels (i.e., random intercept) at either the maximum or minimum value of the rating scale also have near-zero variance (i.e., scale). For example, if the rating scale goes from 1 to 10, then any subject with a mean level near either 1 or 10 would almost certainly have a small variance, giving rise to the potential for a quadratic relationship between the mean and variance. In this regard, **MixWILD** allows for three possibilities to describe the relationship between random intercept and random scale: (1) no association (*τ*_*l*_ = *τ*_*q*_ = 0); (2) linear association only (*τ*_*l*_≠ 0,*τ*_*q*_ = 0); and (3) linear and quadratic association (*τ*_*l*_≠ 0,*τ*_*q*_≠ 0). For a given program run, the user can select one of these three possibilities using the NCOV option, described in “MixWILD Software Overview”.

As described in Hedeker and Nordgren (2013), the parameters of this model (*β*, *α*, *τ*, *τ*_*l*_, *τ*_*q*_, and $\sigma _{\omega }^{2}$) are estimated using maximum likelihood and the Newton–Raphson algorithm. Once the model has converged to a solution, empirical Bayes methods (Bock, 1989) are used to obtain subject-specific estimates for *υ*_*i*_ (random location intercept) and *ω*_*i*_ (random scale), along with the variance-covariance matrix associated with these estimates, which are saved for use in stage 2. These correspond to estimates of the mean and variance-covariance of the posterior distribution of the random effects.

### Mixed-effects multiple location scale (MEMLS) model

Extending the model presented in the previous section, a researcher may be interested in understanding how the slopes of the lines vary by subject for time-varying covariates. Such random slopes can be used to generalize the above model, allowing for a vector of random location effects instead of only a random intercept.

Visually, Fig. 2 shows a simple example. Unlike Fig. 1, the rate of change can vary by subject. The average across all subjects is depicted with the solid gray line, and the location averages (mean plus slope) of two subjects are presented as dashed lines. Hypothetical data points for these two subjects are also included in the plot. In a given dataset, there will be as many dashed lines as there are subjects, but for simplicity only two subjects are plotted.

Relative to the overall (solid) line, the position of each dashed or dotted line when the covariate is equal to zero is indicative of a person’s random intercept location effect *υ*_1*i*_, which indicates how a subject deviates from the mean response. Relative to the solid line, the difference in slope of each dashed or dotted line shows the effect of that subject’s random slope effect *υ*_2*i*_.

In this example, the subject shown with a dotted line has a lower value (negative *υ*_1*i*_) when the covariate value is small, but increases at a faster rate (larger *υ*_2*i*_). How close together the lines are, and how similar the slopes are is indicative of how much subject heterogeneity is observed. Finally, the amount of variation of a subject’s data points (i.e., relative to the dashed or dotted lines) is indicative of that subject’s WS variance. In the example, the subject with open circles is much more tightly clustered (smaller *ω*_*i*_) than the subject with closed circles.

The measurement *y* of subject *i* (*i* = 1,2,…,*N* subjects) on occasion *j* (*j* = 1,2,…,*n*_*i*_ occasions) can be modeled as follows:

2.2.1 $$ y_{ij} = \boldsymbol{x}_{ij}^{\top} \boldsymbol{\beta} + \boldsymbol{z}_{ij}^{\top}\boldsymbol{\upsilon}_{i} + \epsilon_{ij} ,\epsilon_{ij} \sim N(0, \sigma^{2}_{\epsilon_{ij}}), \boldsymbol{\upsilon}_{i} \sim N\left( \mathbf{0}, \boldsymbol{\Sigma}_{\upsilon}\right), $$

where

2.2.2 $$ \sigma_{\epsilon_{ij}}^{2}  =  \exp(\boldsymbol{w}_{ij}^{\top} \boldsymbol{\tau} + \boldsymbol{\tau}_{\upsilon} \boldsymbol{\upsilon}_{i}+ \omega_{i}),\omega_{i}\sim N(0,\sigma_{\omega}^{2}). $$

As shown above, the random effects and errors are assumed to follow normal distributions, and errors are assumed to be independent of the random effects.

In Eq. 2.2.2, ***w***_*ij*_ is a vector of regressors for the WS variance (typically including a “1” for the intercept as the first element) and ***τ*** is the corresponding vector of regression coefficients. These could be the same or different variables as in ***x***_*ij*_, and can be at the subject level, vary across occasions, or be interactions of subject-level and occasion-level variables.

Also in Eq. 2.2.2, the random scale effect (*ω*_*i*_) allows the WS variance to vary across subjects beyond the contribution of covariates. Similar to the random location effect in Eq. 2.0.1, the covariates entered in a model may not account for all of the reasons that subjects differ from each other.

As in Hedeker and Nordgren (2013), an association between the location and scale random effects can be induced by including the location random effects as predictors in the within-subjects variance model, using ***τ***_*υ*_, which are terms from the Cholesky decomposition of the variance/covariance matrix. In this regard, **MixWILD** allows for two possibilities to describe the relationship between random location and random scale: (1) no association (***τ***_*υ*_ = **0**) or (2) association (***τ***_*υ*_≠**0**). For a given program run, the user can select one of these two possibilities using the NCOV option, described in “MixWILD Software Overview”.

As in the MELS model, empirical Bayes methods (Bock, 1989) are used to obtain estimates of the multiple random location effects ***υ***_*i*_ and random scale effect *ω*_*i*_, along with the variance-covariance matrix associated with these estimates. These correspond to estimates of the mean and variance-covariance of the posterior distribution of the random effects. These are saved for use in stage 2.

## Stage 2: linear or logistic regression using stage 1 estimates

Once the subject-specific location (intercept and/or slope) and scale estimates for the random effects have been obtained, they may be used in subsequent stage 2 analyses. However, since these are estimates, the degree of certainty/uncertainty in these estimates needs to be included in the stage 2 analyses. For this, similar to the concept of multiple imputation for missing data, a number of datasets are created (i.e., re-sampled) using the mean and variance estimates augmented by random number generation. Since the random effects are assumed to have come from a normal distribution, multiple imputed values are obtained from a multivariate normal distribution with means and variance/covariance as estimated. This results in multiple datasets, each with a single set of imputations of the random effects. The number of datasets created is set by the reader; generally it is wise to use a large number, say 500, to ensure more precise results.

These stage 1 random effects can then be used to model a stage 2 subject-level outcome that is either continuous (linear regression) or binary/ordinal (logistic regression). Additionally, other subject-level covariates can be included as main effects and interactions with the random effects in the stage 2 model. The stage 2 analyses are repeated for each set of imputed random effect estimates, and after all the analyses have been performed, overall means and standard errors are obtained (similar to what is done in multiple imputation) to produce the stage 2 output.

## MixWILD Software Overview

**Fig. 3:**
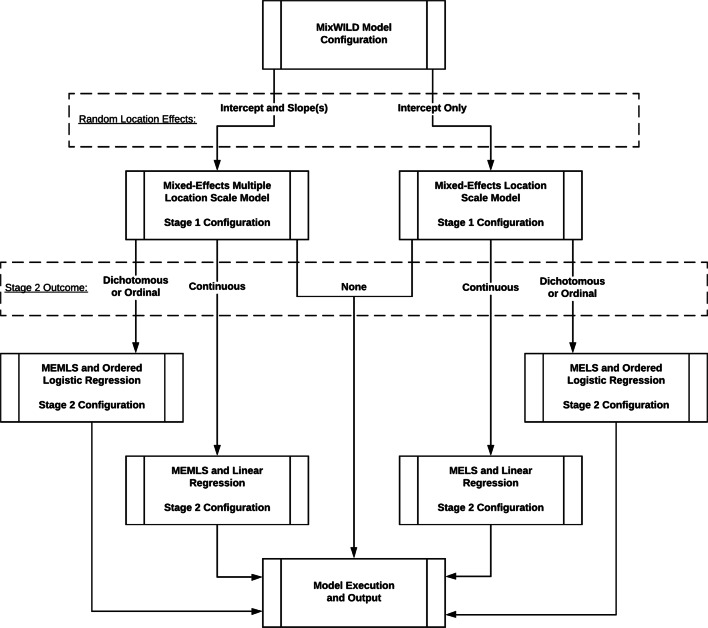
A model flowchart of **MixWILD** components

The **MixWILD** software is used to assist users in adding model parameters and displaying output of the analysis without relying on a command-line interface. It allows users to select the data file to process, assign missing value codes, add or remove regressors from different levels, and adjust other miscellaneous parameters specific to model execution. Figure 3 illustrates the flow of parameter selection in MixWild and how the selection of random location effects and stage 2 outcome impacts the execution of the various modeling stages. **MixWILD** software implements a model-view-controller (MVC) framework (Burbeck, 1992), with the **MixWILD** graphical user interface (GUI) acting as the view and its interactive components as the controller. The variable definition library, acting as the model, specifies parameters and exposes getter and setter functions to the view and controller. The defined parameters are then saved to disk to be accessed by **MixWILD** binaries for execution of statistical procedures. The **MixWILD** GUI has been developed using JAVA and is compatible with both the Windows and macOS operating systems. Figure 3 illustrates a model flowchart of different **MixWILD** components.

As shown in the figure, users first specify how random location effects will be modeled, either as intercept only or as intercept and slope(s). This specification informs how the software proceeds to the stage 1 configuration, providing the model-specific user interface for MELS or MEMLS as needed. In the same configuration menu, users specify the type of outcome at stage 2, which leads to a customized user interface at stage 2 for dichotomous/ordered logistic regression or linear regression if a user specifies dichotomous/ordinal or continuous, respectively. If the user indicates no stage 2 outcome, the stage 2 configuration menu is bypassed and no stage 2 model is executed. A total of four permutations of models exist in the version v1.0-beta.7, with output from models separated by stage 1 and stage 2.

### Creating a new model

**Fig. 4:**
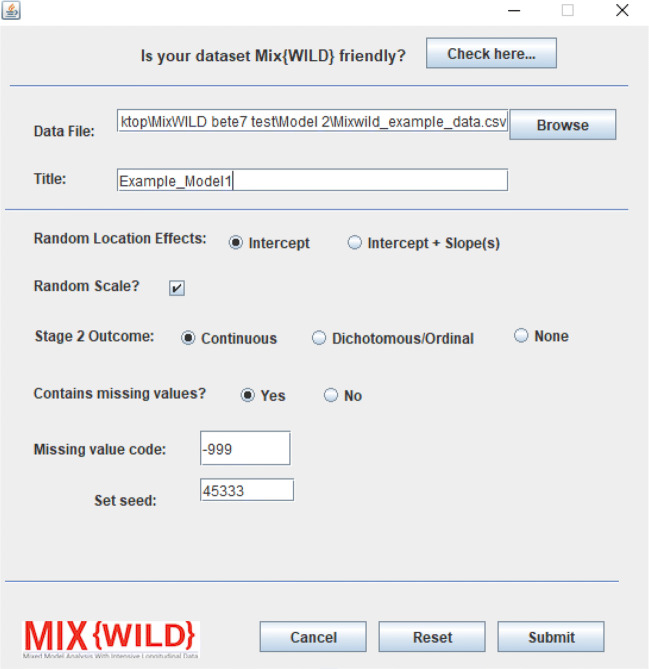
Create a new model by importing the data file and setting model parameters

To create new models, users can access the New Model option under the File menu. Prior to specifying model parameters, users are asked to identify the location of their data. In order to ensure that the file is compatible with **MixWILD**, users can click on the instructions on the top of the window as shown in Fig. 4. For a dataset to be valid for **MixWILD**, it must:

1. be saved as a valid comma-separated (.csv) file,
2. not contain blank missing values,
3. contain only real, non-zero numeric missing value codes (if missing values are present),
4. be sorted by the unique level 2 identifier (e.g., ID variable), and
5. contain variable names in the header.

Once a valid data file is imported, model specification options are enabled. Users can assign a short custom title to identify their model for future reference. A subtitle is automatically generated to distinguish models written by users and those automatically generated by **MixWILD**. The title and subtitle are displayed as headers in the definition file (explained in a subsequent section).

Users first specify whether they would like to include random slopes in addition to an intercept for the random location effects in the stage 1 model. Selecting Intercept Only assumes the mean of the response does not differ between subjects as a result of some covariate and engages the MELS model, allowing users to specify covariates for WS and BS variances. On the other hand, selecting Intercept and Slope(s) engages the MEMLS model, allowing users to test for differences in the association between the response and time-varying covariates (i.e., random slope). Note that this option does not permit the BS variances to be modeled in terms of covariates. As an additional option, users may choose to disable random scale (i.e., WS variance which varies by subject) when running more traditional multilevel models at stage 1 or when there is insufficient within-subject variation to allow for it to vary at the subject level.

Next, users are asked to specify whether the stage 2 outcome will be continuous or dichotomous/ordinal; alternately, users may forgo a stage 2 model entirely. If the model includes a stage 2 outcome, then **MixWILD** allows users to set a seed that varies between 1 and 65,000. Users can edit the default seed randomly chosen by **MixWILD**. Finally, users may specify a non-zero numeric code that matches the missing value codes in the dataset, if any exist. **MixWILD** defaults to no missing values to emphasize that it does not recognize the presence of missing values on its own, and therefore, users must be aware of the missing value codes used in their datasets. After specifying the new model parameters, users are asked to configure the stage 1 and stage 2 models.

### Stage 1 configuration

**Fig. 5:**
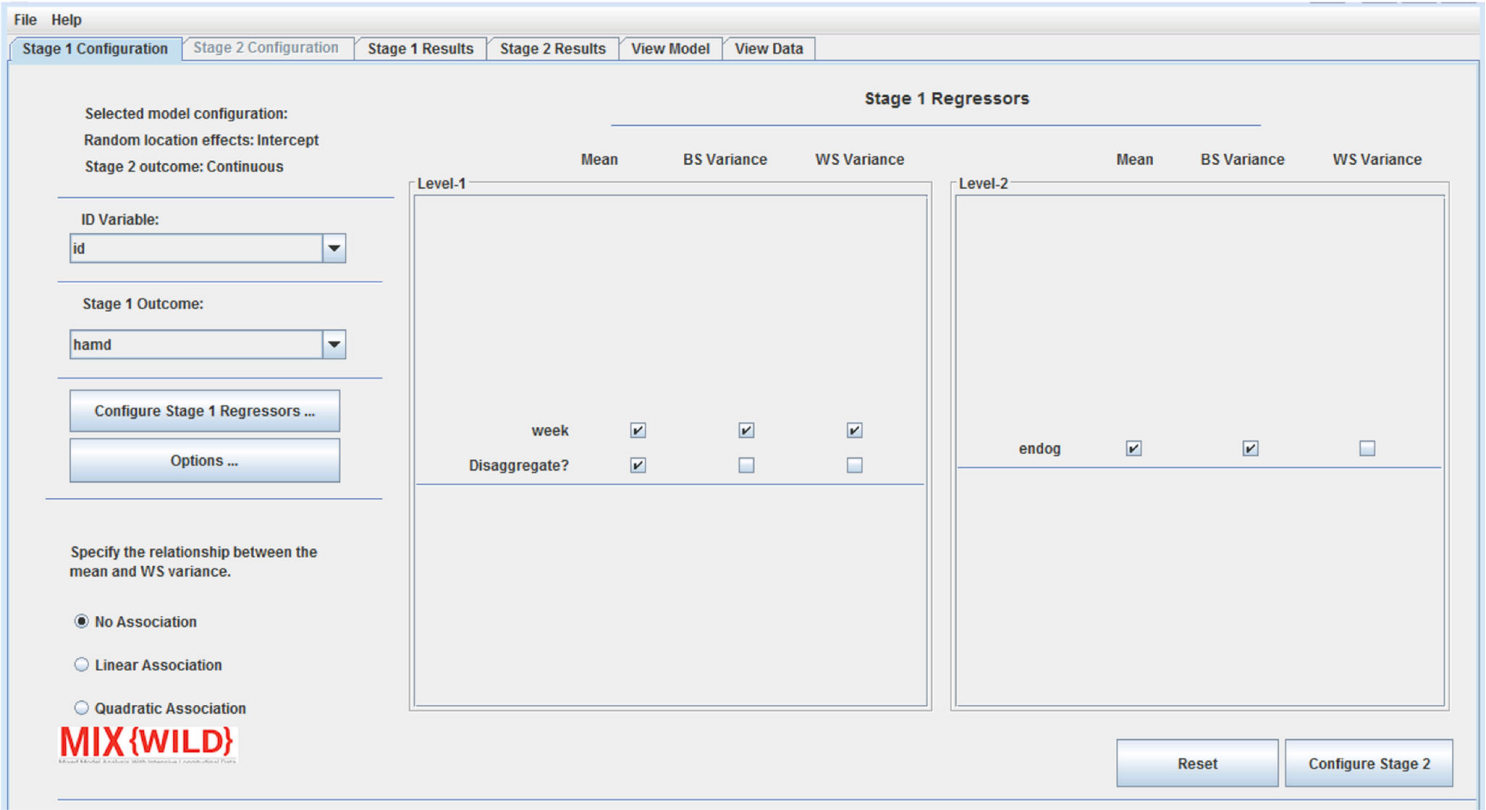
Stage 1 configuration that allows adding variables to level 1 and level 2 of stage 1. Level 1 and level 2 regressors are added from stage 1 regressor window (explained in the next section)

Prior to proceeding with stage 1 configuration, users may choose to validate their data using the View Data tab. Figure 5 provides a screenshot of the stage 1 model configuration. For reference, the selected model configuration, as specified in the New Model window, is displayed on this screen. First, users can define their ID variable (i.e., the unique identifying value for each subject in the study) and the stage 1 outcome variable from the drop-down boxes. By default, the interface uses the first and second column to automatically choose ID and stage 1 outcome, respectively. Next, users are able to select regressors from their data using the Configure Stage 1 Regressors button.

#### Add stage 1 regressors

**Fig. 6:**
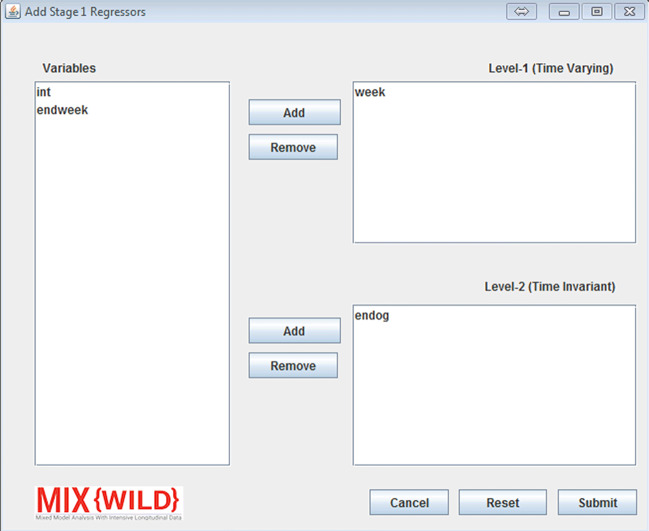
Add regressors from the data file to level 1 and level 2 of stage 1

From the stage 1 configuration window, users are able to add or remove regressors used in the model as shown in Fig. 6. Level 1 variables are time-varying variables and level 2 includes time-invariant variables. It is important to note that the software does not validate whether a variable is time-varying or time-invariant. Once a variable is added to either the level 1 or level 2 list, it is hidden from the main variable list to ensure that there are no duplicate variables in both the level 1 and level 2 variable lists. Users can revert a variable to the variable list by removing them from the added list. Once the variables are selected, users can submit their choices to go back to stage 1 configuration. The reset button allows users to restore all variables on the regressor configuration window.

### Configure model-specific attributes

**MixWILD** allows additional model run time parameters that users can specify using the Options button under stage 1 Configuration. Table 1 (Appendix) summarizes the options offered by **MixWILD**, including default values and valid ranges, where applicable.

**Fig. 7:**
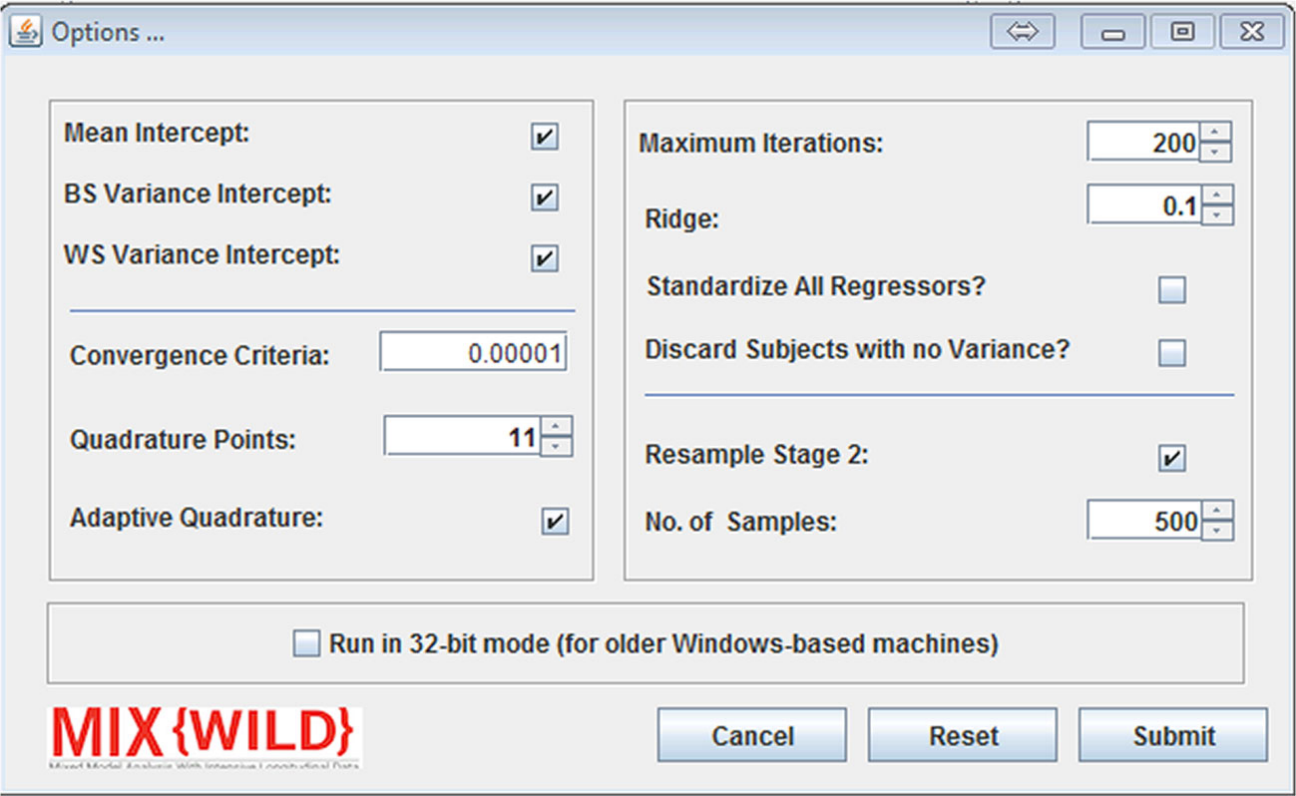
Advanced options to add to the model

Users are then able to submit changes or reset to default. If the software detects that a user is running a Windows operating system, an option appears allowing the user to execute **MixWILD** statistical binaries in an experimental mode for 32-bit operating systems. Figure 7 shows how users can enable different advanced attributes to be included in their models.

### Stage 2 configuration

**Fig. 8:**
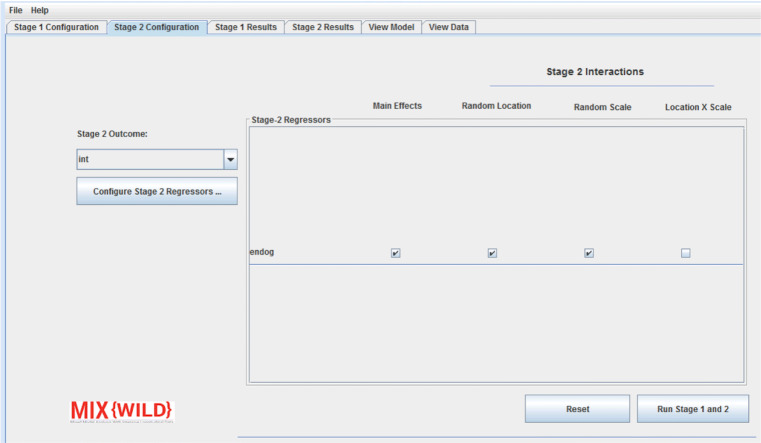
Configure regressors for stage 2 analysis

**MixWILD** validates stage 1 configuration once either the stage 2 configuration tab, the Configure stage 2 button, or the Run Stage 1 button (when no stage 2 outcome is specified) are pressed. Users then proceed to stage 2 configuration, where they are asked to select the time-invariant stage 2 outcome and regressors. Users should note that if a time-varying variable is selected, the subject-level mean will be used throughout the stage 2 model, but the program will not output warning messages to indicate this transformation. If a dichotomous or ordinal stage 2 outcome is specified, an option to check categories will be presented as a convenience feature for users to verify that their variable is valid. Once stage 2 regressors are added, users can specify main effect and two-way and three-way with random location and random scale (if applicable) from stage 1 (as seen in Fig. 8). The regressor by random location interaction may be either a single regressor by random intercept interaction or two interactions: regressor by random intercept and regressor by random slope. However, in both cases, the three-way interaction will only use the random intercept component of the random location (i.e., regressor by random intercept by random scale). An interaction of location by scale is automatically specified in every stage 2 model, but may be disabled by checking the box Suppress All Interactions, which limits the model to the main effects of stage 2 regressors, random location, and random scale (if applicable).

#### Specify model parameters

As seen in Fig. 6, the stage 1 regressor configuration window allows users to specify the contribution of previously selected variables in level 1 (time-varying) and level 2 (time-invariant) tables. For each variable in level 1 and level 2, users can select their contribution to the stage 1 outcome using appropriate checkboxes. Under level 1, users are able to disaggregate the pooled effects of time-varying covariates into between- and within-subject components by generating a level 2 subject mean centered variable and a level 1 deviation from the subject mean variable, respectively (see Appendix). The stage 1 user interface changes dynamically to conform to specifications in the MELS and MEMLS models. If a user indicates a MELS model, they are asked to specify whether a linear or quadratic relationship between the mean and within-subject variance will be included in the model. Further, **MixWILD** will request specification of mean-level model (i.e., betas), BS variance, and WS variance regressors. If a multiple location effects model is selected, the user has the option to allow for an association between the random location (intercept and slope(s)) and within-subject variance. **MixWILD** will then present the user with the option to specify mean, random slope, and scale regressors. Note, random slope specification is excluded from level 2 regressors because level 2 observations have no within-subject variance. A reset button allows users to reset all the changes and restart model configuration.

### Variable definition library

**Fig. 9:**
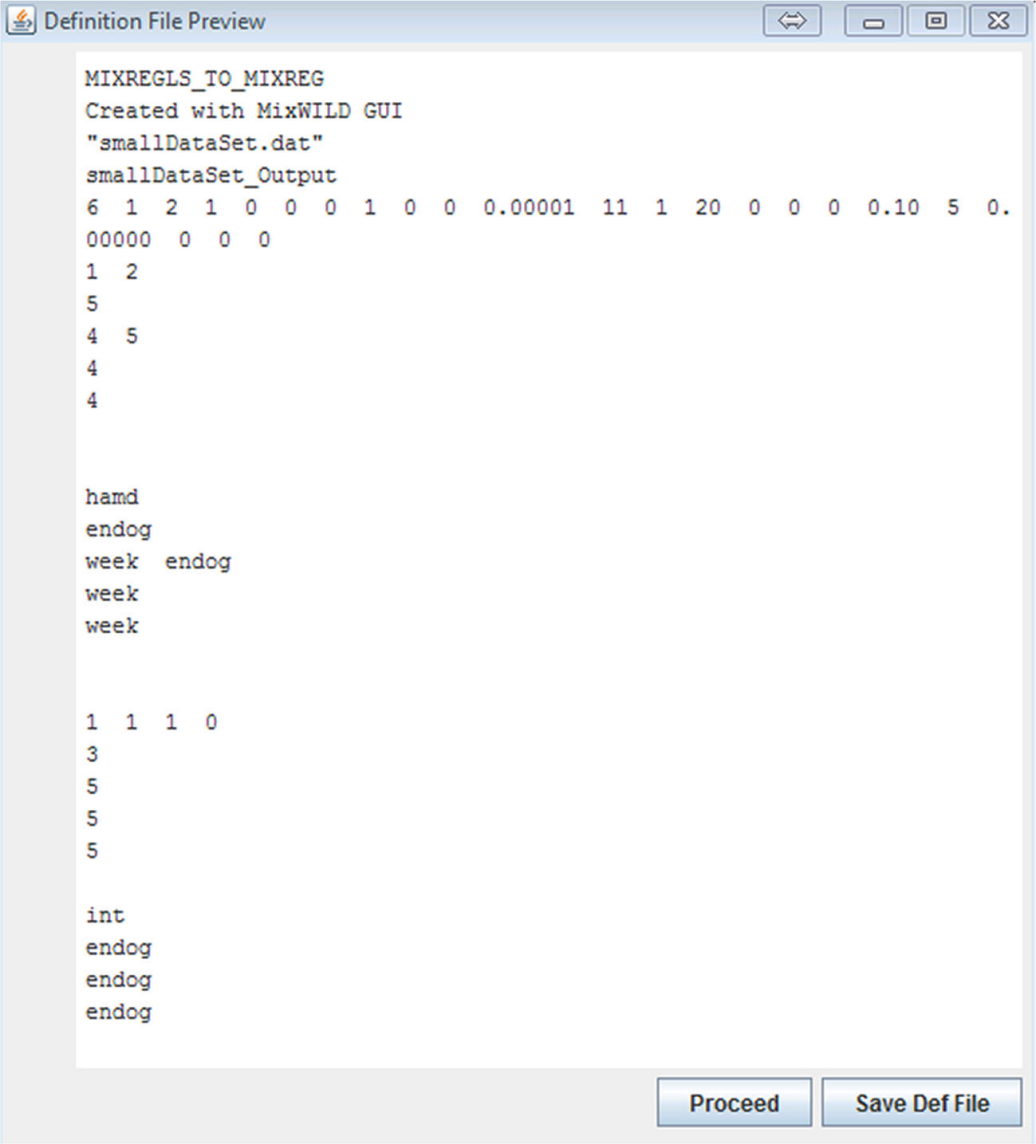
Variable definition preview. Users can save the .def file for later reference

The **MixWILD** variable definition library performs a final validation on stage 1 and stage 2 configuration prior to generating an intermediate definition file (.def) that is saved to the working directory. The intermediate file is generated by translating each parameter from the **MixWILD** model to a plain-text format readable by statistical binaries. The external definition file is subsequently accessed by the **MixWILD** interface to present users with a preview of the definition file prior to proceeding with model execution (as shown in Fig. 9).

### Executable models

The GUI relies on packaged executables, which correspond to the four permutations of statistical procedures available in **MixWILD**: Stage 1 MELS models with linear or logistic stage 2 regressions (MixregLS Mixreg and MixregLS Mixor, respectively) and stage 1 MEMLS models with linear or logistic stage 2 regressions (MixregMLS Mixreg and MixregMLS Mixor, respectively). On model execution, **MixWILD** selects the appropriate executables to read the intermediate definition file and run the selected models. The progress, executed in a background command-line shell, is presented during execution in plain-text for troubleshooting logging purposes. If the model fails, the log is not deleted to allow the user to identify the source of the error.

**MixWILD** copies these executables in a local folder of the user’s system, where folder name is generated in real-time using a system time stamp as a float value in milliseconds since January 1st, 1970 (i.e., Unix epoch time). This process allows for logging of all analyses performed using **MixWILD** based on the time stamp of when the program was accessed. More importantly, it reduces errors and allows for troubleshooting in the event that a model fails. By generating output and definition files in isolated folders, **MixWILD** prevents conflicts across different sessions as a result of identically-named files. Limiting the redundancy in the **MixWILD** work folder as a result of multiple filenames allows users to troubleshoot models in-depth when errors are encountered. In addition to viewing progress after a model is complete, users can see model execution progress in a pop-up window as shown in the image below. As soon as the analysis is complete and successful, the copied executables are deleted from the local folder. As a result, if the model fails, the user can implement command-line tools to quickly rerun binaries in order to identify the cause of errors. The user may also choose to archive the folder and send the persistent session to others for additional support.

To create optimized **MixWILD** executables, preprocessor directives are read at compile-time to indicate whether the OS is a 32-bit or 64-bit Windows machine. If neither is detected, macOS is assumed and 64-bit Unix binaries are generated instead. This method allow for streamlined development using a single source code, with differences only in file-system-specific lines where the command-line shell (i.e., command prompt vs. bash) is called. There are descriptive statistics and three sets of sub-model results in the stage 1: the first sub-model does not include scale parameters (i.e., standard multilevel model), the second sub-model includes scale parameters but not random scale parameters, and the third sub-model includes both the scale and the random scale parameters. The simulated subject-specific random effects are saved to a data file with the suffix *ebvar.dat*. The stage 2 output includes descriptive statistics and final model summary from either the linear or logistic regression.

### Model output

**Fig. 10:**
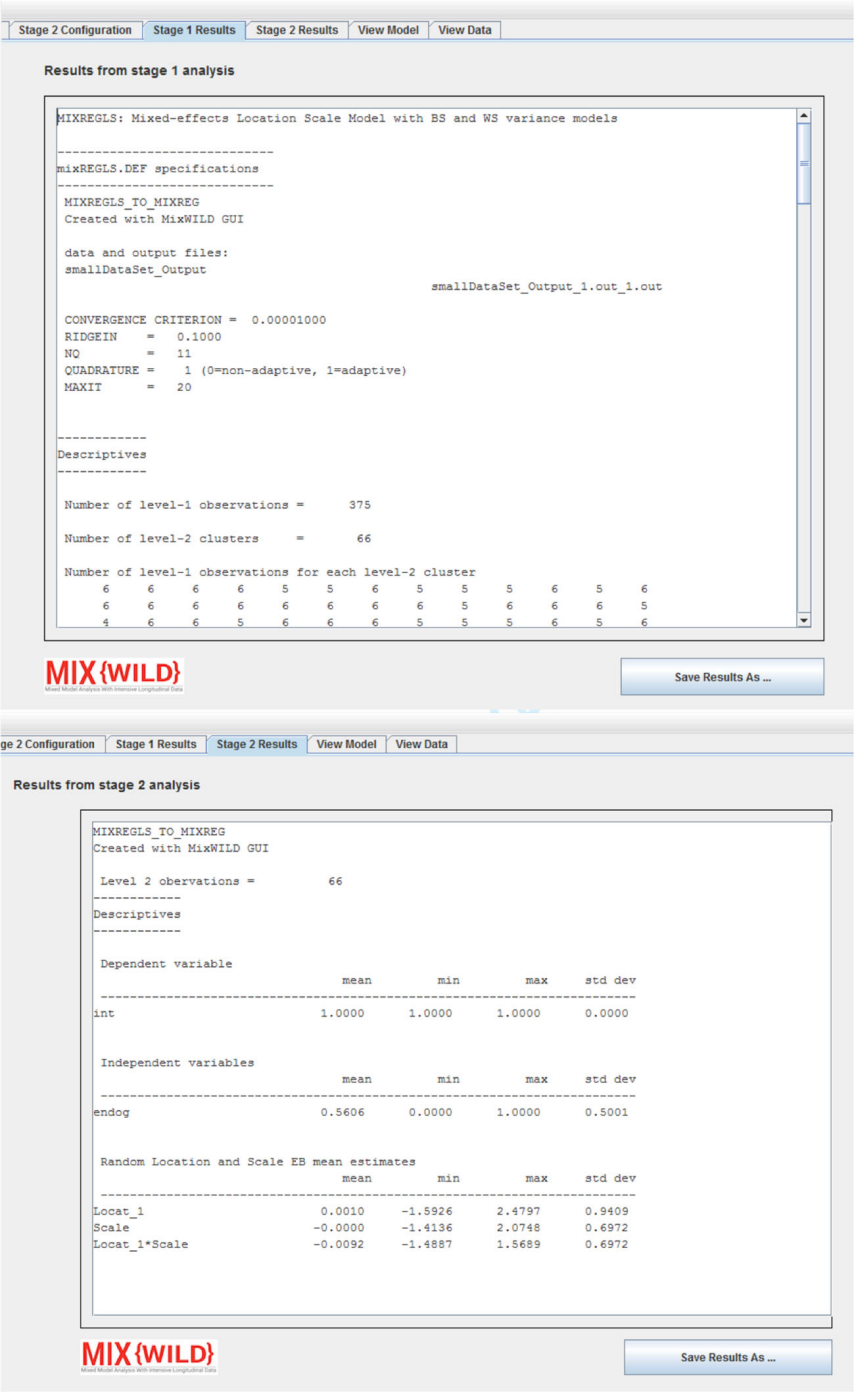
Stage 1 (top) and stage 2 (bottom) analysis output

When models are executed successfully, the output of stage 1 and stage 2 analyses are displayed in their respective tabs in **MixWILD** (as shown in Fig. 10 top and bottom). Users can choose to save the output files outside of the working directory, as well as specify alternate file extensions. As a convenience, **MixWILD** also allows users to copy the output text directly from the output window to the system clipboard. A GitHub-hosted website (http://github.com/reach-lab/MixWildGUI) is available for users to sign up for prompt updates to the application.

## Applied examples

To better understand the types of questions that can be addressed using the two-stage mixed effects approach, two examples will be illustrated, covering both the MELS model and the MEMLS model. In the **first example**, the software first estimates a mixed-effects location scale model in stage 1, including a random subject intercept and a random subject scale effect. As stated prior, a random subject intercept effect reflects a subject’s mean (or location), whereas a random scale effect reflects a subject’s variability. For this example, the stage 2 component is a single-level linear regression model predicting a continuous subject-level outcome using the random subject effects from the stage 1 model as regressors, with the option of including random effects as main effects and interactions with other subject-level regressors. This **second example** first estimates a MEMLS model in stage 1, including a random subject intercept and slope, as well as a random subject scale effect. Hence, the random subject intercept and slope are considered location effects because they reflect a subject’s mean response, while the random subject scale effect reflects a subject’s variability. In this example, the stage 2 component is a single-level logistic regression model that predicts a binary or ordinal subject-level outcome using the random subject effects from the stage 1 model as main effects or interactions with other subject-level regressors. Neither of the examples presented here were formally preregistered. They are presented here for the purpose of demonstrating **MixWILD** software’s use for the analysis of EMA data for behavioral research. However, the data and code for the software can be made available to researchers on request.

### Does subject-level change in positive affect (PA) and variation in PA predict daily sedentary time?

The first applied example is in the context of a multi-method longitudinal study utilizing momentary self-reports of positive affect collected from smartphones and physical activity data collected from waist-worn accelerometers (Maher et al., 2019). The primary aim of the study is to determine whether within-subject mean (i.e., random intercept) and within-subject variance (i.e., random scale) of momentary positive affect (a within-subject, continuous, time-varying variable) predicts between-subject average sedentary hours per day (a between-subject, continuous, time-invariant variable), after controlling for sex (a between-subject, categorical, time-invariant variable) and day of the week at stage 1 and age (a between-subject, continuous, time-invariant variable) at stage 2. Day of the week is coded as a continuous, within-subject, time-varying variable coded such that Monday = 0 and Sunday = 6, hence a linear association can be interpreted as each day approaching the end of the week. Further, the study seeks to understand whether subjects’ age (a continuous, between-subject, time-invariant variable) moderates the effect of subjects’ mean (i.e., random intercept) and variance (i.e., random scale) in momentary positive affect in predicting subject-level average hours per day of sedentary behavior, after controlling for sex and day of week. The study will employ a MELS model using MixWILD, followed by a stage 2 linear regression using estimates of random components from stage 1.

For stage 1, subjects *i* = 1,2,…,*N*, occasions *j* = 1,2,…,*n*_*i*_:

5.1.1 $$ \begin{array}{@{}rcl@{}} \texttt{pa}_{ij} &=& \beta_{0}+\beta_{1}\texttt{dow}_{ij}+\beta_{2}\texttt{sex}_{i}+ {\upsilon}_{i}\\ &&+ \epsilon_{ij} ,\epsilon_{ij} \sim N(0, \sigma^{2}_{\epsilon_{ij}}), {\upsilon}_{i} \sim N(0, \sigma^{2}_{{\upsilon}_{ij}}), \end{array} $$

where

5.1.2 $$ \sigma_{{\upsilon}_{ij}}^{2}  =  \exp(\alpha_{0}+\alpha_{1}\texttt{sex}_{i}), $$

and

5.1.3 $$ \sigma_{\epsilon_{ij}}^{2}  =  \exp(\tau_{0}+\tau_{1}\texttt{sex}_{i}+ \tau_{l} {\upsilon}_{i}+ \omega_{i}),   \omega_{i}\sim N(0,\sigma_{\omega}^{2}).  $$

For stage 2, subjects *i* = 1,2,…,*N*:

5.1.4 $$ \begin{array}{@{}rcl@{}} \texttt{adsh}_{i} &=& \beta^{*}_{0}+\beta^{*}_{1}\texttt{age}_{i}+ \beta^{*}_{2}\hat{\upsilon}_{i}+\beta^{*}_{3}(\hat{\upsilon}_{i} \times \texttt{age}_{i}) +\beta^{*}_{4}\hat{\omega}_{i}\\ &&+\beta^{*}_{5}(\hat{\omega}_{i}\times\texttt{age})_{i} + \beta^{*}_{6}(\hat{\upsilon}_{i}\times\hat{\omega}_{i})+\epsilon^{*}_{i} ,\\ \epsilon_{i}^{*} &\sim& N(0, \sigma^{2}_{\epsilon^{*}}). \end{array} $$

*β*^∗^ is used to designate the fixed effects in stage two (Eq. 5.1.4) as different from those in stage one (Eq. 5.1.1) and *𝜖* ∗ is used to distinguish the error terms.

#### Model specification

**Fig. 11:**
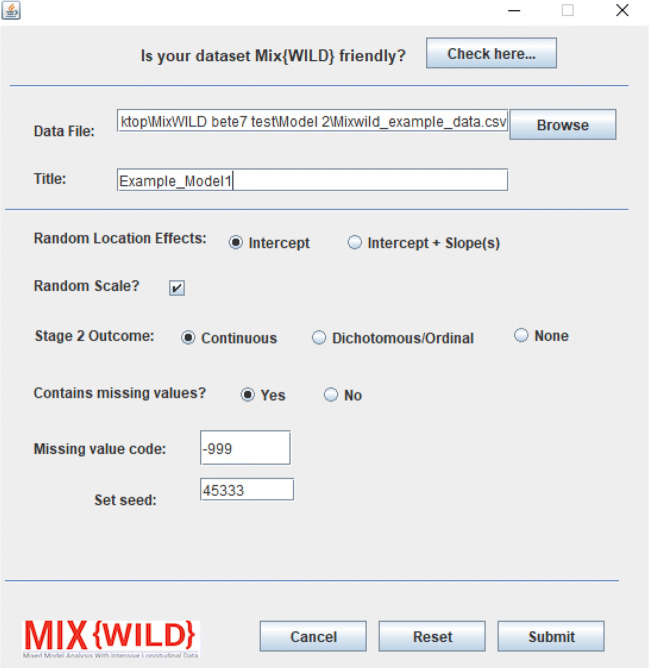
Configure model parameters for a two-stage MELS model

The model is configured in **MixWILD** using the following parameters after specifying a data file location and title (see Fig. 11):

1. **Random Location Effects:** Here, Intercept is specified, thus telling the software to assume only a random subject intercept, but allowing modeling of covariates on between-subject variance.
2. **Random Scale:** Random scale is left enabled by default as the study question examines how the outcome varies within subjects.
3. **Stage 2 Outcome:** The stage 2 outcome in this model is a continuous variable, hence Continuous is specified.
4. **Contains Missing Values and Missing Value Code:** The data contains missing values, specified as -999 in the supplementary dataset.

Next, the ID variable is selected at stage 1, and positive affect is specified as the stage 1 time-varying outcome variable as indicated in Fig. 13 (Appendix). Day of the week (shortened to DOW) is added as a time-varying covariate and is allowed to affect the mean-level model without disaggregation of its effects. Sex (male = 1, female = 0) is added as a time-invariant covariate and is allowed to affect the mean model, the BS variance model, and the WS variance model. Further, the model allows for a linear relationship between the random intercept of the outcome (i.e., WS mean) and random scale (i.e., WS variance). Subjects may report relatively high positive affect based on prior literature, and there is expected to be less variation in these subjects (i.e., ceiling effects (Eid & Diener, 1999)). However, some scales may exhibit floor and ceiling effects, in which case a quadratic relationship may be more appropriate to account for low variance in low valence responses. The model options are left at defaults, therefore assuming intercepts in the mean model, BS variance, and WS variance equations. Once stage 1 is configured, average sedentary hours per day is set as the subject-level stage 2 outcome and regressors are selected as indicated in Fig. 14 (Appendix). For this specific research question, age is entered in the model and selected to interact with random intercept and random scale, without specifying a three-way interaction. Once the model configuration is accepted and executed, the resulting output is displayed, shortened for readability in subsequent blocks of text.

#### Stage 1 results

Excerpted results from stage 1 are shown below, with only the final sub-model shown. A series of three models (with the subsequent model using the previous model’s coefficients as starting values) is run to increase stability and allow comparisons with and without random scale. The final model shows that as day of the week increases by 1 unit, mean positive affect increases by 0.39 units (*z* = 8.58,*p* < .001), while sex does not have a significant effect on mean positive affect (*z* = − 1.21,*p* = .23). There is also no significant effect of sex on either the between-subject variability (*z* = − 1.32,*p* = .19) or the within-subject variability (*z* = − 1.08,*p* = .28), after adding the random scale effect. There is significant variability in scale across subjects, as indicated by the random scale standard deviation; a significant random scale standard deviation indicates that subjects differ from each other in their degree of WS variance (i.e., scale) (*z* = 19.58,*p* < 0.001). Further, the final sub-model shows that, as anticipated, the random scale is negatively associated with the random intercept (*z* = − 6.45,*p* < 0.001). Hence, subjects with overall higher mean positive affect had less WS variability in their momentary responses, likely as a result of a ceiling effect in the affect response scale.

Log Likelihood = -47914.216Akaike's Information Criterion = -47923.216Schwarz's Bayesian Criterion = -47945.843Variable Estimate AsymStdError z-value p-value------------------------- ------------ ------------ ------------ ------------BETA (regression coefficients)Intercept 42.57868 0.55031 77.37179 0.00000DOW 0.39158 0.04563 8.58138 0.00000SEX -0.73865 0.60904 -1.21281 0.22520ALPHA (BS variance parameters: log-linear model)Intercept 4.28473 0.09386 45.65031 0.00000SEX -0.14654 0.11109 -1.31913 0.18713TAU (WS variance parameters: log-linear model)Intercept 4.78679 0.03843 124.56564 0.00000SEX -0.04782 0.04447 -1.07528 0.28225Random scale standard deviationStd Dev 0.40712 0.02080 19.57251 0.00000Random location (mean) effect on WS varianceLoc Eff -0.14581 0.02253 -6.47252 0.00000

#### Stage 2 results

The results from stage 2 are presented below. The stage 2 results table contains the intercept, subject-level regressors (in this case, age) predicting the outcome (average hours per day in sedentary time), the effect of the subject-level mean (i.e. random location denoted as Locat_1) and any interactions (denoted as scale) on sedentary time, the effect of within-subject variance (i.e., random scale) and any interactions on sedentary time, and the interaction between random intercept and random scale on sedentary time, any specified three-way interactions (in this case, none), and the residual variance. After controlling for all other variables, age was positively associated with average daily sedentary time, such that older subjects spend more time being sedentary (*z* = 4.07,*p* < 0.001). Neither a subject’s mean nor variance predicts their average daily sedentary time, nor are these associations moderated by age (*p* > 0.05).

Average Log Likelihood = -2106.858 (sd= 0.754)Akaike's Information Criterion = -2113.858Schwarz's Bayesian Criterion = -2131.457Variable Estimate AsymStdError z-value p-value------------------------- ------------ ------------ ------------ ------------Intercept 9.37497 0.06913 135.61136 0.00000Age 0.01809 0.00445 4.06683 0.00005Locat_1 0.07526 0.06594 1.14148 0.25367Locat_1*Age -0.00311 0.00362 -0.86029 0.38963Scale 0.03044 0.08354 0.36436 0.71559Scale*Age -0.00054 0.00475 -0.11386 0.90935Locat_1*Scale -0.00667 0.08898 -0.07493 0.94027Residual_Variance 2.45392 0.10338 23.73673 0.00000

### Do day of week differences in positive affect predict obesity risk?

The second applied example is in the context of a longitudinal study utilizing momentary self-reports of positive affect collected from smartphones and exploring affect-related obesity risk among subjects (Maher et al., 2019). The primary aim of the study is to examine whether within-subject mean (i.e., random intercept) and within-subject variance (i.e., random scale) of momentary positive affect (a within-subject, continuous, time-varying variable) predicts subject-level obesity risk (a between-subject, dichotomous, time-invariant variable), after controlling for sex (a between-subject, categorical, time-invariant variable), whether a momentary response was provided on the weekday or weekend (a within-subject, dichotomous, time-varying variable) at stage 1, and age (a between-subject, continuous, time-invariant variable) at stage 2. Additionally, the study seeks to understand whether subjects differ from each other in the extent to which positive affect changes on weekends as compared to weekdays, after controlling for subject-level mean and subject-level variance (i.e., the random slope of weekend/weekday in terms of positive affect) at stage 2. The last set of aims seek to understand whether: (a) the variability between subjects in the association (i.e., random slope) between weekday/weekend and momentary positive affect predicts subject-level obesity risk, (b) the age of a subject moderates the associations between mean levels (i.e., random intercept) and variances (i.e., random scale) in positive affect in predicting obesity risk, and (c) the age of a subject could moderate weekend-positive affect association (i.e., random slope) in predicting obesity risk. The study will employ a MEMLS model using MixWILD, followed by a stage 2 logistic regression using estimates of random components from stage 1.

For stage 1, subjects *i* = 1,2,…,*N*, occasions *j* = 1,2,…,*n*_*i*_:

5.2.1 $$ \begin{array}{@{}rcl@{}} \texttt{pa}_{ij} &=& \beta_{0}+\beta_{1}\texttt{w\_end}_{ij}+\beta_{2}\texttt{sex}_{i}+ {\upsilon}_{1i} + {\upsilon}_{2i}\texttt{w\_end}_{ij}\\ &&+ \epsilon_{ij} ,\epsilon_{ij} \sim N(0, \sigma^{2}_{\epsilon_{ij}}), \boldsymbol{\upsilon}_{i} \sim N(\mathbf{0}, \boldsymbol{\Sigma}_{\upsilon}), \end{array} $$

where

5.2.2 $$ \sigma_{\epsilon_{ij}}^{2}  =  \exp(\tau_{0}+ \tau_{\upsilon1} {\upsilon}_{1i}+ \tau_{\upsilon2} {\upsilon}_{2i}+\omega_{i}),   \omega_{i}\sim N(0,\sigma_{\omega}^{2}).  $$

For stage 2, subjects *i* = 1,2,…,*N*:

5.2.3 $$ \begin{array}{@{}rcl@{}} logit(P[\texttt{obese}_{i}=1]) &=& \beta^{*}_{0} + \beta^{*}_{1}\texttt{age}_{i} + \beta^{*}_{2}\hat{\upsilon}_{1i} + \beta^{*}_{3}(\hat{\upsilon}_{1i}\times \texttt{age}_{i})\\ &&+ \beta^{*}_{4}\hat{\upsilon}_{2i} + \beta^{*}_{5}(\hat{\upsilon}_{2i}\times \texttt{age}_{i})\\ &&+\beta^{*}_{6}\hat{\omega}_{i} + \beta^{*}_{7}(\hat{\omega}_{i}\times\texttt{age}_{i}) + \beta^{*}_{8}(\hat{\upsilon}_{1i}\times\hat{\omega}_{i})\\ &&+ \beta^{*}_{9}(\hat{\upsilon}_{1i}\times\hat{\omega}_{i}\times \texttt{age}_{i}) \\ &&+ \beta^{*}_{10}(\hat{\upsilon}_{2i}\times\hat{\omega}_{i}) + \beta^{*}_{11}(\hat{\upsilon}_{2i}\times\hat{\omega}_{i}\times\texttt{age}_{i}).\\ \end{array} $$

As in the previous section, *β*^∗^ is used to designate the fixed effects in stage two (Eq. 5.2.3) as different from those in stage one (Eq. 5.2.1).

#### Model specification

**Fig. 12:**
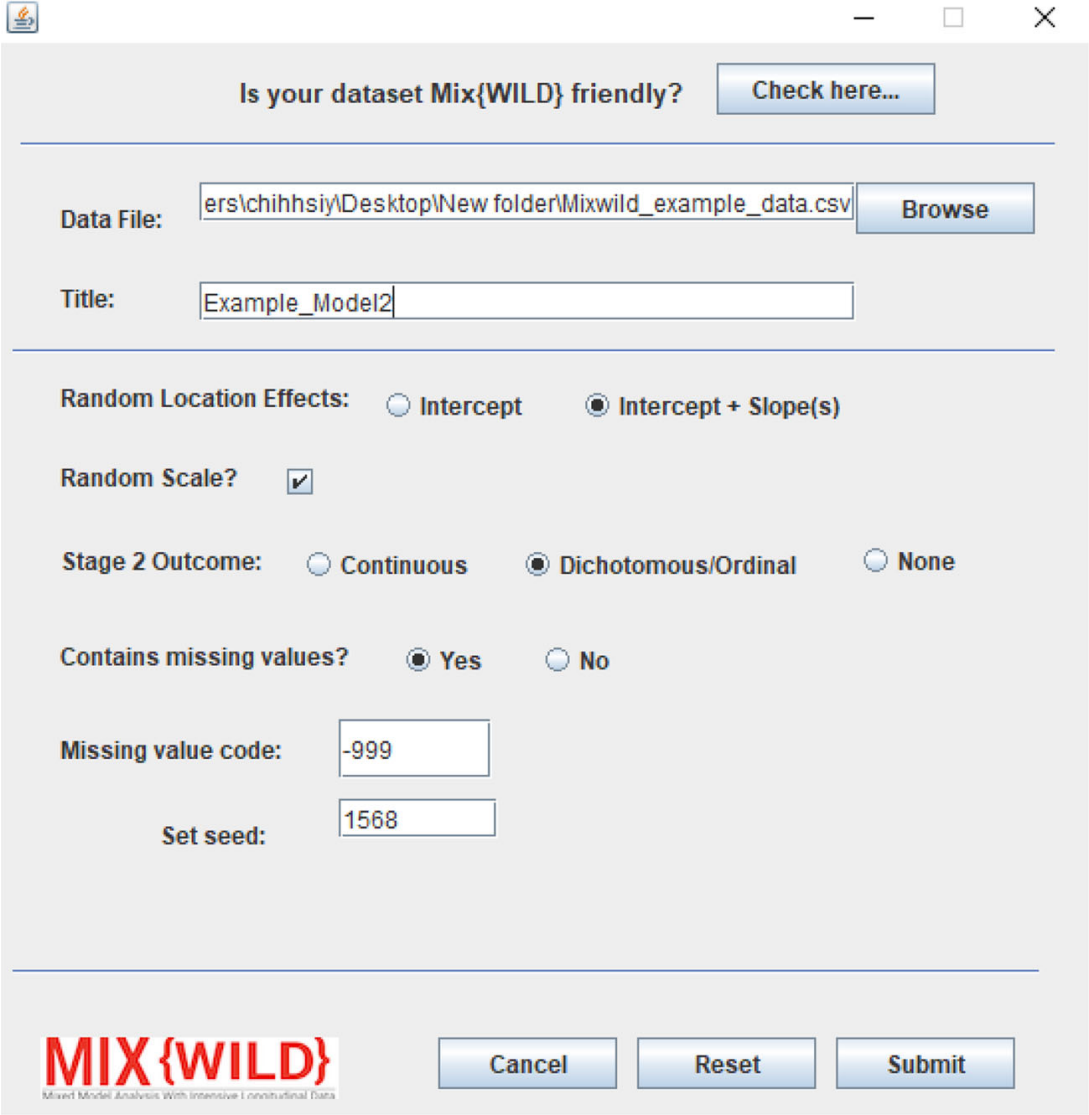
Configure model parameters for a two-stage MEMLS Model

**Fig. 13:**
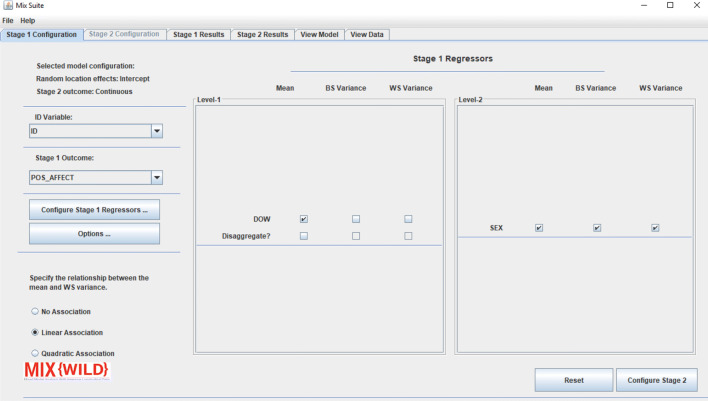
Configure stage 1 regressors for a two-stage MELS Model

**Fig. 14:**
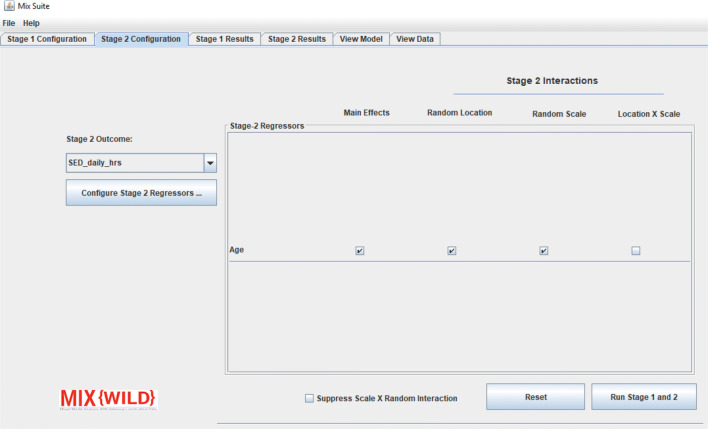
Configure stage 2 regressors for a two-stage MELS Model with a continuous outcome in stage 2

The model is configured in **MixWILD** using the following parameters after specifying a data file location and title (see Fig. 12):

1. **Random Location Effects:** Here, Intercept and Slope(s) is specified, thus telling the software to constrain the modeling of effects on between-subject variance, but allow for modeling of multiple random location effects (intercept and one or more slopes).
2. **Random Scale:** Random scale is left enabled by default as the study question examines how the outcome varies within subjects.
3. **Stage 2 Outcome:** The stage 2 outcome in this model is a dichotomous variable, hence Dichotomous is specified.
4. **Contains Missing Values and Missing Value Code:** The data contains missing values, specified as -999 in the supplementary dataset.

**Fig. 15:**
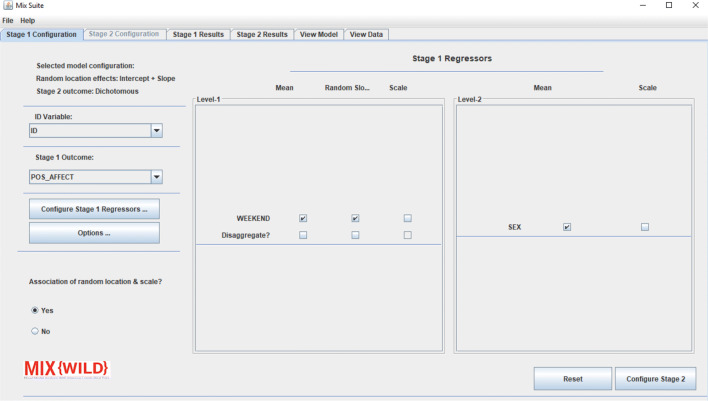
Configure stage 1 regressors for a two-stage MEMLS Model

**Fig. 16:**
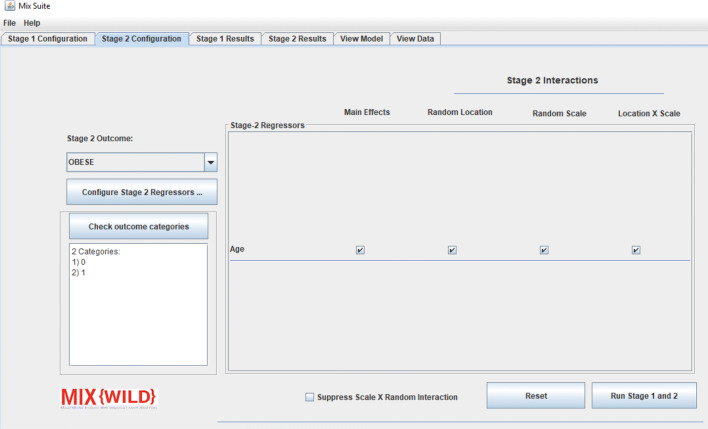
Configure stage 2 regressors for a two-stage MEMLS Model with a categorical outcome in stage 2

Next, the ID variable is selected at stage 1, and positive affect is specified as the stage 1 time-varying outcome variable as indicated in Fig. 15 (Appendix). Whether an observation takes place on a weekday (coded as 0) or weekend (coded as 1) is added as a time-varying covariate and is allowed to affect the mean and random slope without disaggregation of effects. Sex (male = 1, female = 0) is added as a time-invariant covariate and is allowed to affect the mean only. The model has no regressors on the WS variance (i.e., random scale). However, the model tests for association between person-level mean and within-subject variance of the outcome, as is indicated by the specified association of the random location and random scale effects. The model options are left at defaults, therefore assuming intercepts in the mean, BS variance, and WS variance equations. Once stage 1 is configured, whether or not a subject is obese (obese = 1, not obese = 0) is set as the person-level stage 2 outcome and regressors are selected as indicated in Fig. 16 (Appendix). For this specific research question, age is entered in the model and selected to interact with random location and random scale. A three-way interaction is specified between age, random location, and random scale on obesity risk. Once the model configuration is accepted and executed, the resulting output is displayed, shown in abbreviated form in subsequent text blocks.

#### Stage 1 results

Excerpted results from stage 1 are shown below, with only the final sub-model shown. A series of three models (with the subsequent model using the previous model’s coefficients as starting values) is run to increase stability and allow comparisons with and without random scale. The final model shows that there was greater positive affect on the weekend as compared to weekdays, and no sex differences for positive affect in the example (*z* = 7.06,*p* < 0.001 and *z* = − 1.15,*p* = 0.25, respectively). There is significant variability in scale across subjects, as indicated by the random scale standard deviation (*z* = 19.26,*p* < 0.001). In other words, subjects differ from each other in their degree of within-subject variability. It also shows that subjects differed significantly between each other based on mean levels of positive affect (i.e., random location intercept) and differed in their association between weekend and positive affect (i.e., random slope as indicated by the weekend regressor) (*z* = 18.33,*p* < 0.001 and *z* = 5.54,*p* < 0.001, respectively). The random intercept and random slope were negatively associated with each other (*Covariance12*), indicating that subjects with higher mean levels of positive affect on weekdays (i.e., higher levels of the intercept) did not show as much increase in positive affect on weekends, relative to subjects with lower positive affect on weekdays (*z* = − 4.16,*p* < 0.001). Lastly, there was no significant association between random slope and WS variance (i.e., random location effects on WS variance); in other words, erratic positive affect in a subject was not associated with change in positive affect on weekend days relative to weekdays (*z* = − 1.05,*p* = 0.29).

Log Likelihood = -49747.866Akaike's Information Criterion = -49757.866Schwarz's Bayesian Criterion = -49783.190Variable Estimate AsymStdError z-value p-value------------------------- ------------ ------------ ------------ ------------BETA (regression coefficients)Intercept 42.96071 0.50136 85.68796 0.00000WEEKEND 1.67701 0.23744 7.06279 0.00000SEX -0.65533 0.56931 -1.15108 0.24970Random (location) Effect Variances and CovariancesIntercept 71.74435 3.91378 18.33122 0.00000Covariance12 -9.81601 2.35683 -4.16493 0.00003WEEKEND 14.26340 2.57335 5.54274 0.00000TAU (WS variance parameters: log-linear model)Intercept 4.71828 0.01968 239.78506 0.00000Random location effects on WS variance (log-linear model)Intercept -0.12884 0.02304 -5.59199 0.00000WEEKEND -0.03716 0.03530 -1.05270 0.29248Random scale standard deviationStd Dev 0.40514 0.02103 19.26143 0.00000

#### Stage 2 results

The stage 2 results table contains the intercept, subject level regressors (in this case, age) predicting the subject-level outcome (obesity, as a dichotomous variable), the effect of the subject-level mean (i.e., random intercept denoted as Locat_1) and any interactions on obesity risk, the effect of the within-subject association (i.e., random slope denoted as Locat_2) between weekday/weekend and positive affect and any interactions on obesity risk, the effect of within-subject variance and any interactions on obesity risk, and the interaction between random intercept and random scale on obesity risk, and any specified three-way interactions (in this case, with age). After controlling for all other variables, age was positively associated with increased obesity risk (i.e., older subjects are more likely to be obese than younger subjects)(*z* = 6.25,*p* < 0.001). The random intercept for positive affect negatively predicts obesity risk when age equals zero (equivalent to age of 29 years) and the random scale and random slope are zero (*z* = − 2.66,*p* < 0.01). Since the random effects are centered around zero, a random scale of zero represents the average scale. For subjects with average scale of positive affect, higher levels of mean positive affect are associated with reduced obesity risk. In this model, random slope did not significantly predict obesity risk (*z* = 0.26,*p* = 0.80). Finally, the interaction between age and random scale was significant in predicting obesity risk, suggesting that the positive association of age and obesity risk is more pronounced for subjects that are more erratic or less stable in their momentary positive affect response (*z* = 3.17,*p* < 0.005). In other words, subjects who are older and who have higher variability in positive affect are more likely to be obese. All other interactions were not significant.

Average Log Likelihood = -633.437 (sd= 3.667)Akaike's Information Criterion = -643.437Schwarz's Bayesian Criterion = -668.761Variable Estimate AsymStdError z-value p-value------------------------- ------------ ------------ ------------ ------------Intercept -0.24106 0.13037 -1.84905 0.06445Age 0.05172 0.00828 6.24617 0.00000Locat_1 -0.26560 0.09986 -2.65974 0.00782Locat_1*Age 0.00534 0.00599 0.89198 0.37241Locat_2 -0.02665 0.13925 -0.19139 0.84822Locat_2*Age 0.00258 0.00913 0.28207 0.77789Scale 0.16841 0.12401 1.35805 0.17445Scale*Age 0.02499 0.00788 3.17250 0.00151Locat_1*Scale 0.16108 0.15288 1.05363 0.29205L*S*Age -0.00128 0.00977 -0.13116 0.89565

## Conclusion and future work

This paper presented **MixWILD**, a GUI implementation of a novel statistical software that can be used to enhance inferences made from intensive longitudinal data, such as those gathered using EMA. Although **MixWILD** may be used as a basic hierarchical modeling tool to test hypotheses in clustered data with many observations, researchers interested in how variability of predictors affects their outcome of interest will benefit most from this approach, as illustrated in the applied examples. Other permutations of **MixWILD** cover additional study hypotheses. For instance, a researcher interested in physical activity may examine whether moderate-vigorous physical activity (MVPA) has significant WS variability (i.e., random scale) across the week at stage 1, and then examine whether this variability of MVPA predicts obesity risk at stage 2. Similarly, a researcher interested in mood disorders may hypothesize that subjects vary in their relationship between momentary anhedonia and sedentary behavior at stage 1, and subsequently examine whether this association (i.e., random slope) predicts change over time in a standard depression inventory. However, the interactive component and statistical back-end of **MixWILD** have several limitations.

The interactive component of **MixWILD** is still in active development, with features such as the ability to open **MixWILD** archives (i.e., previously run models) and automatically generated models with regressors (under View Model) expected to be implemented soon. The statistical component of **MixWILD** is limited by its inability to run three-level models at stage 1, run two-level models at stage 2, or use count outcomes at stage 2, the latter two of which is currently under development. The software currently provides Maximum Likelihood (ML) estimates, a further addition would be to add Restricted Maximum Likelihood (REML). Moreover, **MixWILD** and its statistical models do not support R, SAS, and STATA procedures and we hope to develop them as part of our future work.

We have proposed a two-stage modeling approach in MixWILD, however simultaneous joint modeling can also be used in some cases for similar purposes. However, as pointed out by Murawska et al., (2012), if only the variables in the first stage mixed model (and not the second stage outcome model) provide information about the random effects, then it is more appropriate to separate the estimation. Conceptually, we can imagine a case where the first stage is estimated using the first wave of an EMA dataset, and the second stage is based on the second wave. It could be argued that a joint model may not be appropriate in this case, because they are from non-overlapping periods of time.

The supplemental data file allows users to replicate results presented in this manuscript. A website is available at https://reach-lab.github.io/MixWildGUI/ for users to download the latest release sign up for update notices, as well as download additional documentation that includes an updated user guide. All analyses presented in the manuscript were conducted in **MixWILD** Soft Release v1.0-beta.7; some user interface elements may change over time. Change logs and source code for interactive components of **MixWILD** is available at https://github.com/reach-lab/MixWildGUI and source code for the statistical procedure is available at https://github.com/reach-lab/MixWild. The version control system also serves a primary point for users to submit issues and feature requests for the program. A separate Git repository for the statistical component of the code is accessible by contacting the corresponding authors or any member of the development team. Finally, researchers, programmers, and statisticians can also contribute new features to **MixWILD** by accessing our open-source code. All software is licensed under GNU General Public License v3.0 (GPL-3).

### Electronic supplementary material

Below is the link to the electronic supplementary material.

(PDF 6.15 MB)

(CSV 634 KB)

## Appendix A: Terminology

The following section defines terms relevant to intensive longitudinal data analysis that will be referenced throughout the manuscript, the user interface, and program output.

**MixWILD** assumes a standard two-level model with a continuous level 1 outcome at stage 1, where *i* represents each subject and *j* represents each timepoint.

Consider the model

A.0.1 $$ Y_{ij} = \beta_0 + {\upsilon}_{0i} + \epsilon_{ij} $$

**Grand mean**: refers to the mean of the outcome across all time points and subjects, ignoring clustering, as indicated by the intercept in the model (*β*_0_).
**Subject-level mean**: is the mean of the outcome across all time points for a given subject, and specified by including the subject deviation from the intercept in the model (*β*_0_ + *υ*_0*i*_).
**Between-subject variance**: is defined as the variance across subject-level mean ($\sigma ^{2}_{\upsilon }$). In a homogeneous sample of subjects, this value would approach 0.
**Within-subject variance**: is defined as the error variance for a given subject ($\sigma ^{2}_{\epsilon _{ij}}$). This value would be near 0 in a subject whose outcomes are extremely well modeled.

The **mean model** can then be expanded to allow for the addition of covariates. Note that the interpretation of *β*_0_ is now the overall mean when the value of all covariates is 0. *β*_0_ + *υ*_0*i*_ is the subject level mean when all covariates equal 0.

A.0.2 $$ Y_{ij} = \beta_0 + {\upsilon}_{0i} + (\beta_1+{\upsilon}_{1i})X_{ij} + \beta_2 W_i + e_{ij} $$

Definitions of terms that are relevant to covariates in **MixWILD** can be defined by addition of components to the means model:

**Time-invariant covariates**: are level 2 variables measured once per subject, such as socio-demographic variables (*W*_*i*_).
**Time-varying covariates**: are level 1 variables measured multiple times per subject, including temporal variables such as time of day and day of week (*X*_*i**j*_). Given that time-varying covariates produce pooled effects in multilevel models, for those variables that are continuous or binary, additional statistical transformation can be used to disaggregate between- and within-subject effects. Within-subject covariates are created when time-varying covariates are centered at their subject-level mean ($X_{ij}-\bar {X}_{i}$), and those subject-level means become additional variables ($\bar {X}_{i}$). The disaggregation procedure allows for interpretation of within- and between-subjects effects of covariates on the outcome.
**Mean effect**: refers to the relationship between a covariate and the mean of the outcome, this is the equivalent of a fixed effect coefficient in a traditional multilevel model (*β*_1_ or *β*_2_). For example, physical activity may negatively associated with age or positively associated with concurrent positive affect.
**Subject-level mean effect**: refers to the relationship between the covariate and the mean of the outcome for a specific subject. (*β*_1_ + *υ*_1*i*_).
**Between-subject variance effect**: is defined as the relationship between a covariate and the variance of the subject-level means. [Only in MELS model]
**Within-subject variance effect**: refers to the relationship between a covariate and within-subject variance of the outcome.
**Random scale effect**: allows different subjects to have different amounts of within-subject variance, beyond that modeled by covariates. The variance of the random scale effect may be influenced by that subject’s location random effects. For instance, subjects with high mean levels of physical activity may be more consistent in their positive affect, or subjects may be less erratic (i.e., low WS variance) in their positive affect when they are engaged in physical activity.

## Appendix B: Tables

**Table 1:** Advanced options to configure the model

Advanced option	Interaction mode	Usage in models
Mean intercept, BS variance intercept, WS variance intercept	Check-boxes (checked by default)	Include submodel intercepts
Convergence criteria	Spinner (between 0 and 1, with 0.00001 as default)	To set the accuracy level of the model
Quadrature points	Spinner (between 1 to 1,000, with 25 as default)	More quadrature points results in more accurate estimate of integral, but takes more time to execute
Adaptive quadrature	Check-box (checked by default)	To personalize quadrature to each subject
Maximum number of iterations	Spinner (between 1 to 1000, with 200 as default)	To prevent the model from running indefinitely
Ridge	Spinner (between 0 and 1, with 0.1 as default)	To improve convergence for computationally challenging data
Standardize all regressors	Check-box (off by default)	To set variables on the same scale if needed
Discard subjects with no variance	Check-box (off by default)	Subjects with identical values for all observations of the outcome variable can cause estimation problems for the model with random scale; This option excludes such subjects
Resample stage 2	Check-box (checked by default), followed by the number of resamples (between 1 and 10,000, with 200 as default)	To account for the uncertainty in the EB estimates
