# Supplementary material for: MixWILD: A program for examining the effects of variance and slope of time-varying variables in intensive longitudinal data
Source: Behav Res Methods. 2020 Jan 2;52(4):1403–27. doi: 10.3758/s13428-019-01322-1 (PMC7406537; doi:10.3758/s13428-019-01322-1)
Supplement: Supplementary file 1 — (PDF 6.15 MB) [file 13428_2019_1322_MOESM1_ESM.pdf]

THE UNIVERSITY OF CHICAGO   USC University of Southern California

MIXWILD

User's Guide

Mixed Model Analysis With Intensive Longitudinal Data

Donald Hedeker
Genevieve Fridlund Dunton

©2018, University of Chicago & University of Southern California

The development of MixWILD program was supported by the National Heart, Lung, and Blood Institute, National Institute of Health - "Novel Statistical Models for EMA Studies of Physical Activity" Grant 5R01HL121330-05.

We thank Chih-Hsiang (Jason) Yang for his great effort in creating this document.
We also thank Rachel Nordgren, Eldin Dzubur, Aditya Ponnada, and Stephen Intille who contributed heavily to the development of this software.

Special thanks to Chih-Ping Chou, Jimi Huh, and Jaclyn Maher for their helpful comments and suggestions.

The suggested citation for this program is:

MixWILD: A freeware program for mixed model analysis with intensive longitudinal data (in prep).

Table of Contents

1. Overview of Mix Suite: The two-stage approach 1
   1.1. Instructions for downloading the MixWILD program 2
   1.2. Instructions for formatting your dataset to be MixWILD friendly 2
   1.3. Description of the example dataset 3
   1.4. MixWILD compatibility notes for Windows and macOS users 4
2. Example 1: Running a standard multilevel model (MLM) using MixWILD 5
   2.2. Step-by-step instructions for specifying a standard multilevel model 6
   2.2. Brief interpretation of the multilevel model results 14
3. Example 2: Running Mixregls - Linear Regression model in MixWILD 15
   3.1. Step-by-step instructions on running Mixregls - Linear Regression model 16
   4.2. Brief interpretation of the Mixregls - Linear Regression model results 29
4. Example 3: Running Mixregls- Logistic Regression model in MixWILD 31
   4.1. Step-by-step instructions for running Mixregls-Logistic regression model 32
   4.2. Brief interpretation of Mixregls-Logistic regression model results 41
5. Example 4: Running Mixregmls- Linear regression model in MixWILD 43
   5.1. Step-by-step instructions on running Mixregmls - Linear regression model 44
   5.1. Brief interpretation of Mixregmls - Linear Regression model results 55
6. Example 5: Running Mixregmls - Logistic regression model in MixWILD 57
   6.1. Step-by-step instructions for running a Mixregmls- Logistic regression model 58
   6.3. Brief interpretation of Mixregmls-Logistic regression model result 69
7. Appendix A: Steps to take when the program does not converge to a solution 73
8. Appendix B: Steps to plot the random subject effects from the ebvar file solution 76

1. Overview of Mix Suite: The two-stage approach

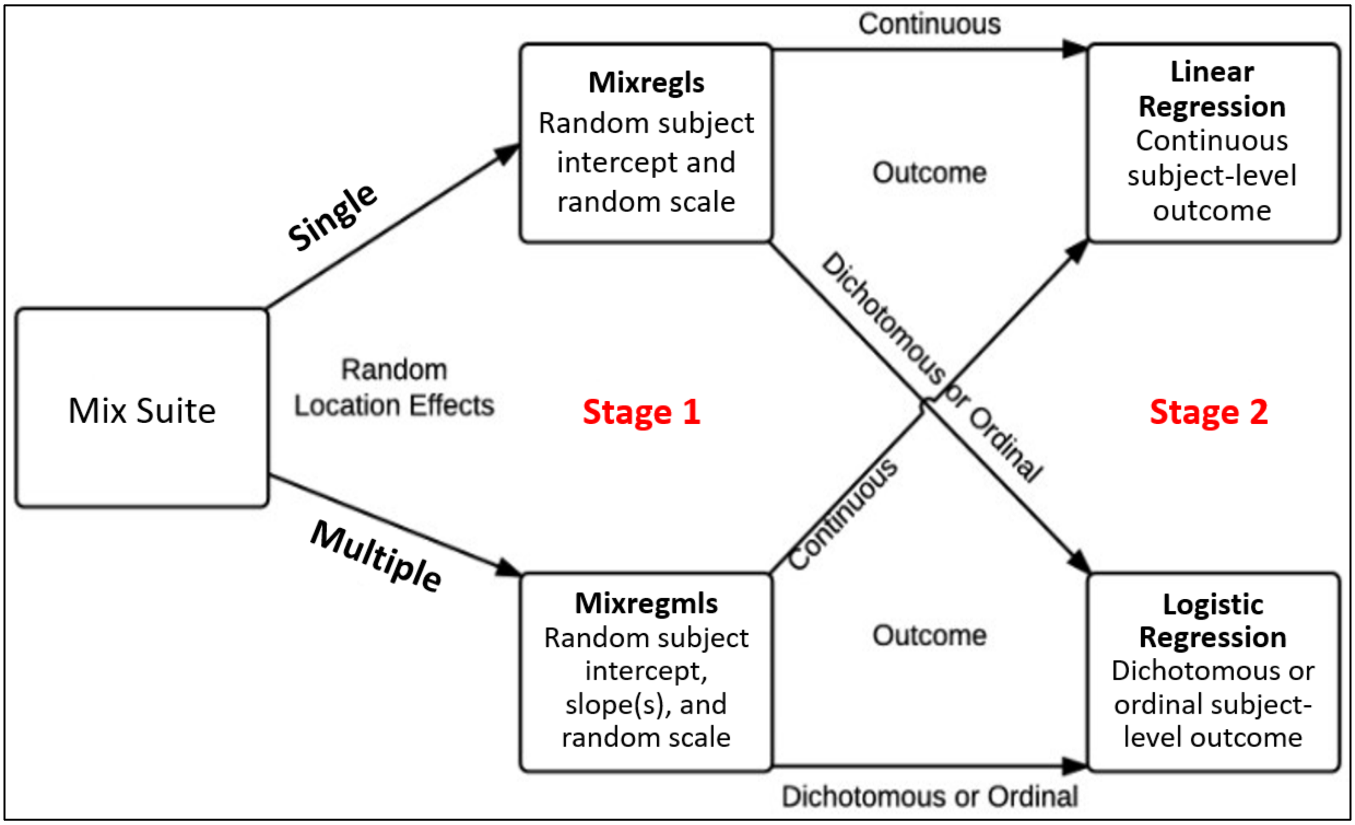

Stage 1 models

Mixregls (Mixed effects Regression Location Scale modeling): This multilevel model estimates a mixed-effects location scale model, including a ***random subject intercept*** and ***a random subject scale effect.*** A random subject intercept effect reflects a subject's mean (or location), and a random scale effect reflects a subject's variability, respectively.

Mixregmls (Mixed effects Regression Multiple Location Scale modeling): This model estimates a mixed-effects location scale model, including a ***random subject intercept and slope(s)***, as well ***as a random subject scale effect***. The random subject intercept and slope(s) are considered location effects because they reflect a subject's mean response, while the random subject scale effect reflects a subject's variability.

Stage 2 models

Linear Regression model: This single level linear regression model predicts a ***continuous subject-level outcome*** using the random subject effects from the Stage 1 model as regressors. The random effects can be included in this linear regression model as main effects and as interactions with other subject-level regressors.

Logistic Regression model: This single level logistic regression model predicts a ***binary or ordinal subject-level outcome*** using the random subject effects from the Stage 1 model as regressors. The random effects can be included in the (binary or ordinal) logistic regression model as main effects and as interactions with other subject-level regressors.

1.1. Instructions for downloading the MixWILD program

1. Visit: https://reach-lab.github.io/MIXWILDGUI/
2. Submit your email prior to downloading the application in the web page to receive notifications on major software updates.
3. Click on macOS (64-bit) or Windows (32-bit) to download the program.
4. Select your directory to save the program.
5. When finished downloading, double-click on the MixWILD icon 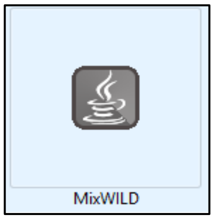

and follow the instructions to complete installation.

1.2. Instructions for formatting your dataset to be MixWILD friendly

1. The dataset should be a **.csv file** with variable names in the **first row**.
2. Data should be in the **long format** and sorted ascending or descending by ID number.
3. Missing values should **not be blank or periods (.)** in the dataset and should be coded as **numeric values** only (e.g., '-999').

Example dataset overview

A	B	C	D	E	F	G	H	I	J	K
ID	AGE	SEX	WEEKEND	DOW	OBESE	BMI	NEG_AFFECT	POS_AFFECT	MVPA_daily_mins	SED_daily_hours
11	7.47	1	1	6	1	22.13	20	44	31.9	8.33
11	7.47	1	0	0	1	22.13	30	44	31.9	8.33
11	7.47	1	1	6	1	22.13	10	46	31.9	8.33
11	7.47	1	1	6	1	22.13	14	47	31.9	8.33
11	7.47	1	0	4	1	22.13	16	47	31.9	8.33
11	7.47	1	1	5	1	22.13	10	49	31.9	8.33
11	7.47	1	1	5	1	22.13	50	49	31.9	8.33
12	5.47	1	0	1	0	21.26	60	19	24.35	8.6
12	5.47	1	0	1	0	21.26	-999	23	24.35	8.6
12	5.47	1	1	6	0	21.26	-999	32	24.35	8.6
12	5.47	1	1	6	0	21.26	-999	33	24.35	8.6
12	5.47	1	0	0	0	21.26	27	35	24.35	8.6
12	5.47	1	1	5	0	21.26	21	36	24.35	8.6
12	5.47	1	1	5	0	21.26	-999	46	24.35	8.6
12	5.47	1	1	6	0	21.26	-999	58	24.35	8.6
12	5.47	1	1	5	0	21.26	12	-999	24.35	8.6
12	5.47	1	1	5	0	21.26	50	-999	24.35	8.6
13	3.47	1	0	0	1	21.12	-999	47	34.56	8.57
13	3.47	1	0	1	1	21.12	50	50	34.56	8.57
13	3.47	1	0	0	1	21.12	-999	53	34.56	8.57
13	3.47	1	1	6	1	21.12	10	60	34.56	8.57
13	3.47	1	1	6	1	21.12	-999	61	34.56	8.57

1.3. Description of the example dataset

Intensive longitudinal data (Number of subjects=1245) from an ecological momentary assessment (EMA) study (range between 4-14 days) will be used as an example dataset to demonstrate different models in the MixWILD program.

Variables in the example dataset

ID: Participant ID number

SEX: 0 (female); 1 (male)

AGE: Number of years (centered around mean age=29.29)

WEEKEND: 0 (weekday); 1 (weekend)

DOW: Day of week; 0: Monday, 1: Tuesday, …, 6: Sunday

POS_AFFECT: Levels of positive affect reported in each prompt

NEG_AFFECT: Levels of negative affect reported in each prompt

MVPA_daily_mins: Daily averaged moderate-to-vigorous physical activity time in minutes

SED_daily_hours: Daily averaged sedentary time in hours

OBESE: 0 (not obese); 1 (obese)

BMI: Body Mass Index (centered around mean BMI=24.66)

Descriptive summary of the example variables

	Variable	Type	Time-varying vs. Time-invariant	N	Mean	SD	Min	Max	Range
1	ID	Nominal	Time-invariant	15167	639.07	393.94	1	1369	1368
2	AGE (centered)	Continuous	Time-invariant	14851	0	16.37	-23.53	47.47	71
3	SEX	Dichotomous	Time-invariant	14899	0.74	0.44	0	1	1
4	DOW	Categorical / Ordinal	Time-varying	15167	3.53	2.25	0	6	6
5	WEEKEND	Dichotomous	Time-varying	15167	0.5	0.5	0	1	1
6	POS_AFFECT	Continuous	Time-varying	13514	43.75	13.7	10	70	60
7	NEG_AFFECT	Continuous	Time-varying	13491	28.91	14.11	10	70	60
8	MVPA_daily_mins	Continuous	Time-invariant	14750	41.96	12.34	15.07	88.46	73.39
9	SED_daily_hours	Continuous	Time-invariant	14094	9.41	1.56	5.35	15.3	9.95
10	OBESE	Dichotomous	Time-invariant	14432	0.47	0.5	0	1	1
11	BMI (centered)	Continuous	Time-invariant	14511	0	6.87	-11.25	23.89	35.14

1.4. MixWILD compatibility notes for Windows and macOS users

The user interface for MixWILD runs in a Java runtime environment that provides feature parity between Windows and Mac versions. Native 64-bit binaries for macOS and Windows written in Fortran are used to execute statistical analyses and generate model output.

- To allow for compatibility with older operating systems and architecture, the Windows version features an option to use 32-bit binaries.

- Users running MixWILD in a virtual machine, such as VMWare or Parallels, should ensure working directories are isolated from hypervisor processes that allow sharing between host and guest. These include common directories such as *Downloads*, *Desktop*, and *Documents*. Instead, create a new folder located at “C:/MixWILD” to improve compatibility.

Suggested references for mixed-effects location-scale model

- Hedeker, D., & Nordgren, R. (2013). MIXREGLS: a program for mixed-effects location scale analysis. *Journal of statistical software*, *52*(12), 1.

- Hedeker, D., Mermelstein, R.J., & Demirtas, H. (2012). Modeling between-subject and within-subject variances in ecological momentary assessment data using mixed-effects location scale models. *Statistics in medicine*, *31*(27), 3328-3336.

- Maher J.P., Dzubur, E., Nordgren, R. Huh, J., Chou, C.P., Hedeker, D., Dunton, G. F. Do fluctuations in positive affective and physical feeling states predict physical activity and sedentary time? *Psychology of Sport and Exercise. In press.*

- Maher, J.P., Huh, J., Intille, S., Hedeker, D., & Dunton, G.F. (2018). Greater variability in daily physical activity is associated with poorer mental health profiles among obese adults. Mental Health and Physical Activity, 14, 74-81.

2. Example 1: Running a standard multilevel model (MLM) using MixWILD

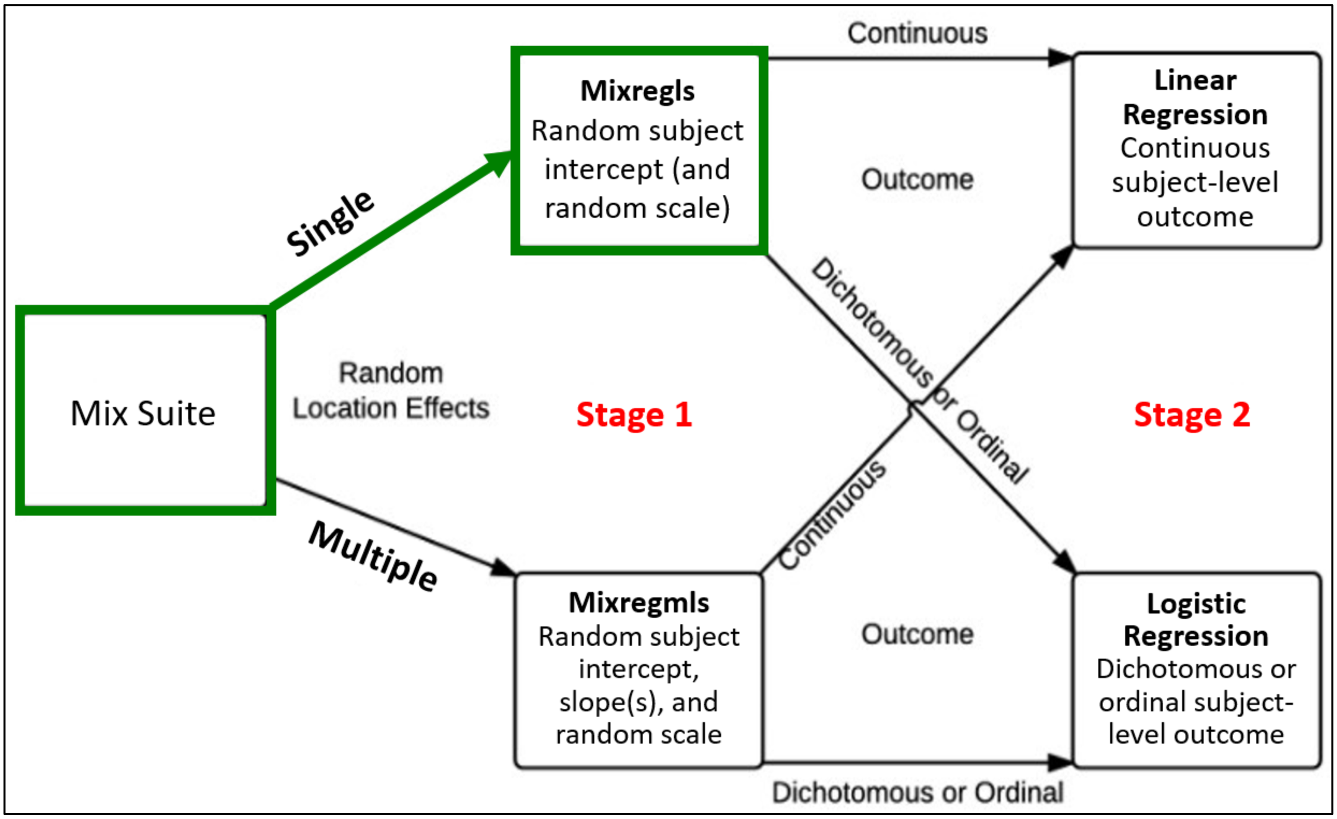

Example question for applying standard multilevel model in MixWILD

- Examine whether participants' momentary negative affect (within-subject, continuous, time-varying variable) can predict their momentary positive affect (within-subject, continuous, time-varying variable), after controlling for sex.

Regressors: Sex (time-invariant); Negative affect (time-varying)
Outcome: Positive affect (time-varying)

Note:

- A standard multilevel model can be specified in the Stage 1 interface by selecting the "None" option for the Stage 2 Outcome.
- Interaction terms are not generated automatically by the Stage 1 interface. If you would like to test interaction(s), you must manually create interaction variable(s) in your dataset before running the program.

2.2. Step-by-step instructions for specifying a standard multilevel model in MixWILD

1. Double-click on the MixWILD icon to open the main window.
2. Click on **“File”** and then select **“New Model”** (or use keyboard shortcut Ctrl + N).

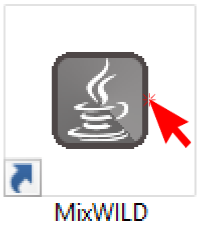

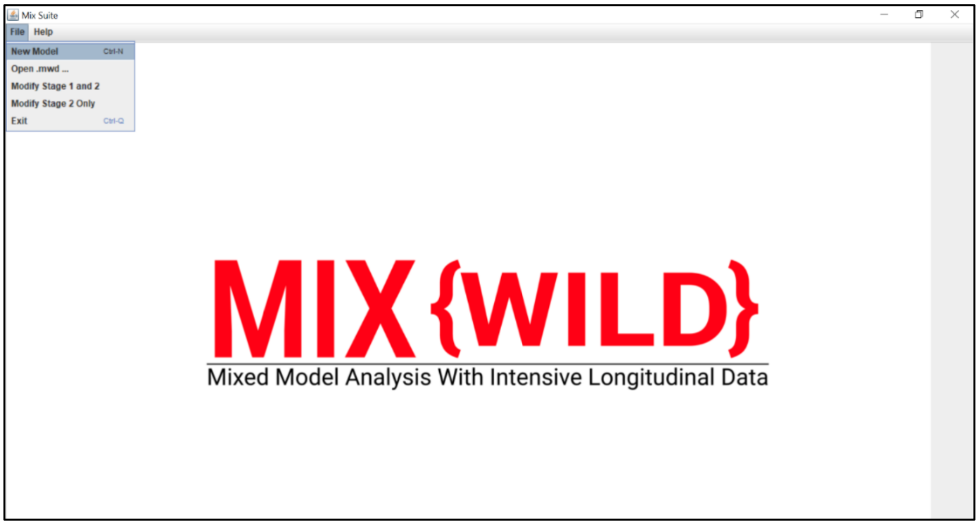

3. Click on **“Instructions”** to make sure your data are in the correct format.

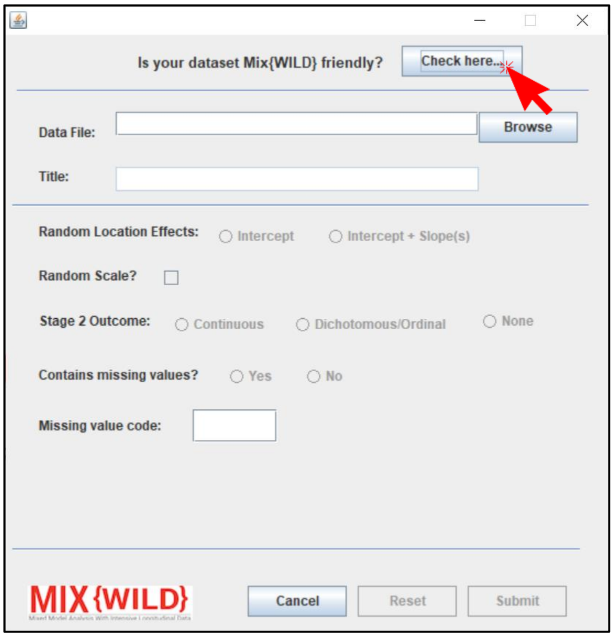

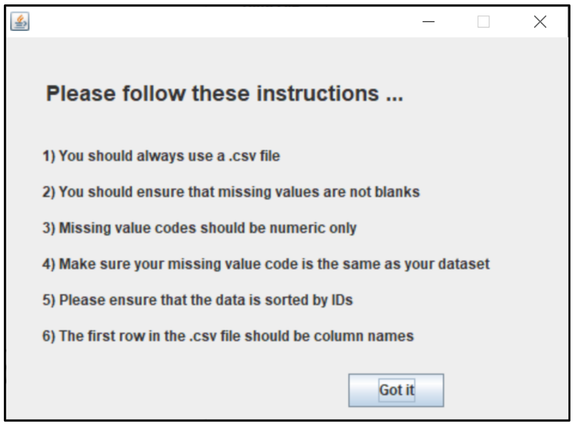

4. Click on **"Browse"** to select the location of your data file (in .csv) and then click **"Open"**.

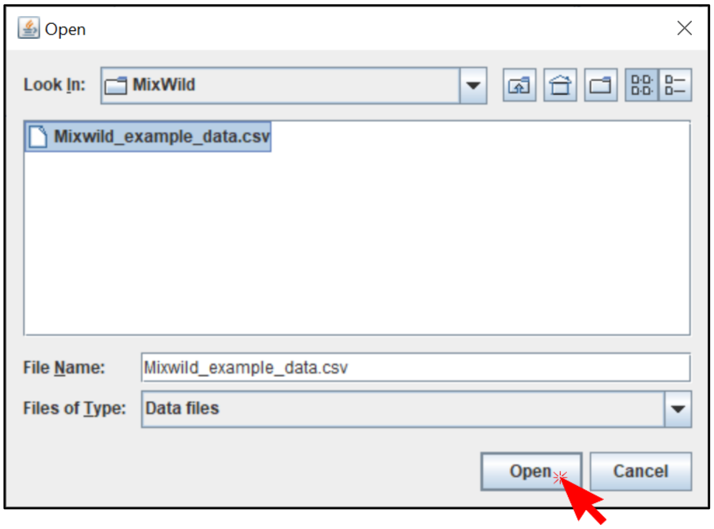

5. Click on **"View Data"** to preview your data file and to verify the data and format are correct.

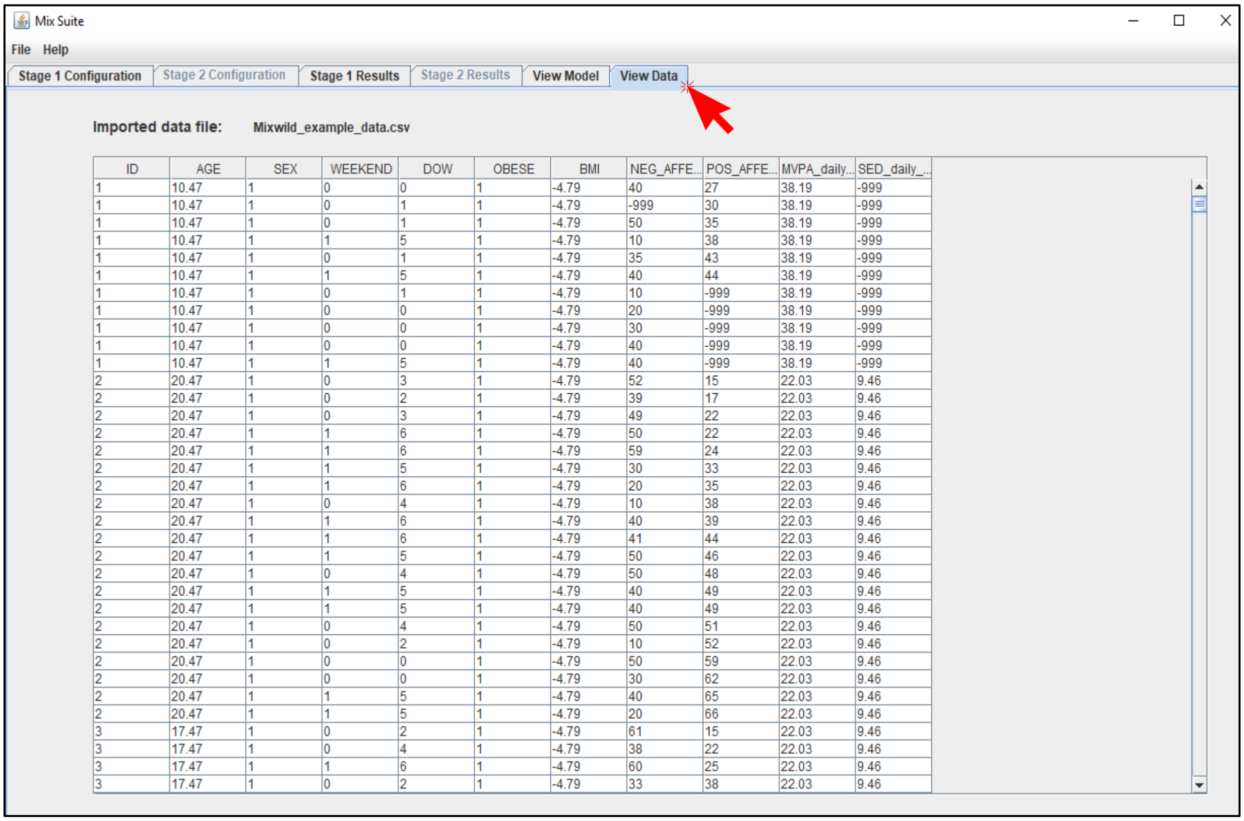

ID	AGE	SEX	WEEKEND	DOW	OBESE	BMI	NEG_AFFE...	POS_AFFE...	MVPA_daily...	SED_daily_...
1	10.47	1	0	0	1	-4.79	40	27	38.19	-999
1	10.47	1	0	1	1	-4.79	-999	30	38.19	-999
1	10.47	1	0	1	1	-4.79	50	35	38.19	-999
1	10.47	1	1	5	1	-4.79	10	38	38.19	-999
1	10.47	1	0	1	1	-4.79	35	43	38.19	-999
1	10.47	1	1	5	1	-4.79	40	44	38.19	-999
1	10.47	1	0	1	1	-4.79	10	-999	38.19	-999
1	10.47	1	0	0	1	-4.79	20	-999	38.19	-999
1	10.47	1	0	0	1	-4.79	30	-999	38.19	-999
1	10.47	1	0	0	1	-4.79	40	-999	38.19	-999
1	10.47	1	1	5	1	-4.79	40	-999	38.19	-999
2	20.47	1	0	3	1	-4.79	52	15	22.03	9.46
2	20.47	1	0	2	1	-4.79	39	17	22.03	9.46
2	20.47	1	0	3	1	-4.79	49	22	22.03	9.46
2	20.47	1	1	6	1	-4.79	50	22	22.03	9.46
2	20.47	1	1	6	1	-4.79	59	24	22.03	9.46
2	20.47	1	1	5	1	-4.79	30	33	22.03	9.46
2	20.47	1	1	6	1	-4.79	20	35	22.03	9.46
2	20.47	1	0	4	1	-4.79	10	38	22.03	9.46
2	20.47	1	1	6	1	-4.79	40	39	22.03	9.46
2	20.47	1	1	6	1	-4.79	41	44	22.03	9.46
2	20.47	1	1	5	1	-4.79	50	46	22.03	9.46
2	20.47	1	0	4	1	-4.79	50	48	22.03	9.46
2	20.47	1	1	5	1	-4.79	40	49	22.03	9.46
2	20.47	1	1	5	1	-4.79	40	49	22.03	9.46
2	20.47	1	0	4	1	-4.79	50	51	22.03	9.46
2	20.47	1	0	2	1	-4.79	10	52	22.03	9.46
2	20.47	1	0	0	1	-4.79	50	59	22.03	9.46
2	20.47	1	0	0	1	-4.79	30	62	22.03	9.46
2	20.47	1	1	5	1	-4.79	40	65	22.03	9.46
2	20.47	1	1	5	1	-4.79	20	66	22.03	9.46
3	17.47	1	0	2	1	-4.79	61	15	22.03	9.46
3	17.47	1	0	4	1	-4.79	38	22	22.03	9.46
3	17.47	1	1	6	1	-4.79	60	25	22.03	9.46
3	17.47	1	0	2	1	-4.79	33	38	22.03	9.46

6. Add a title for your output files. This title name is later displayed in your output files.
7. Select **"Intercept"** from Random Location Effects specification and uncheck **"Random Scale"**.
8. Select **"None"** for Stage 2 Outcome.
9. Click on missing values if there are any in your dataset; specify the missing value code in the box (e.g., '-999').

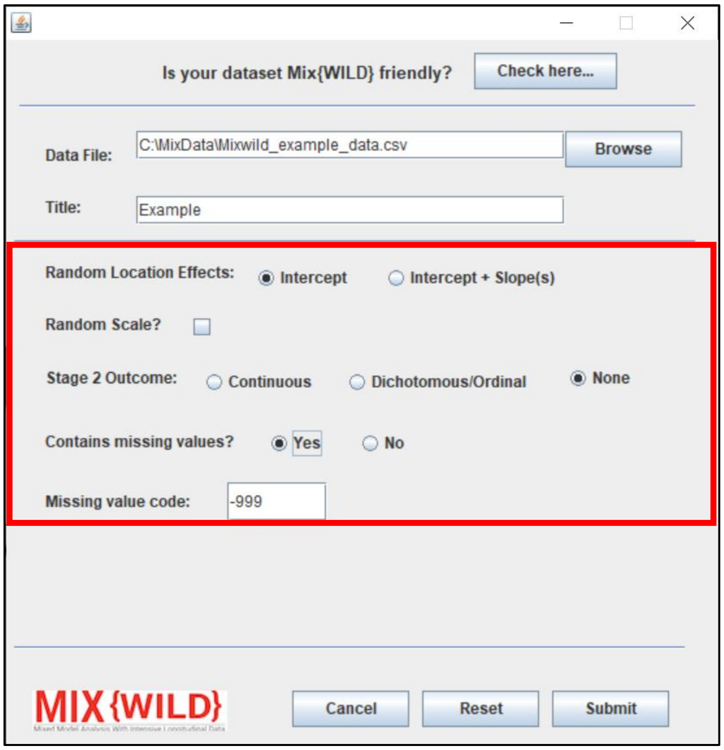

10. After you submit this first page, you will advance to the page that enables you to configure your Stage 1 model.

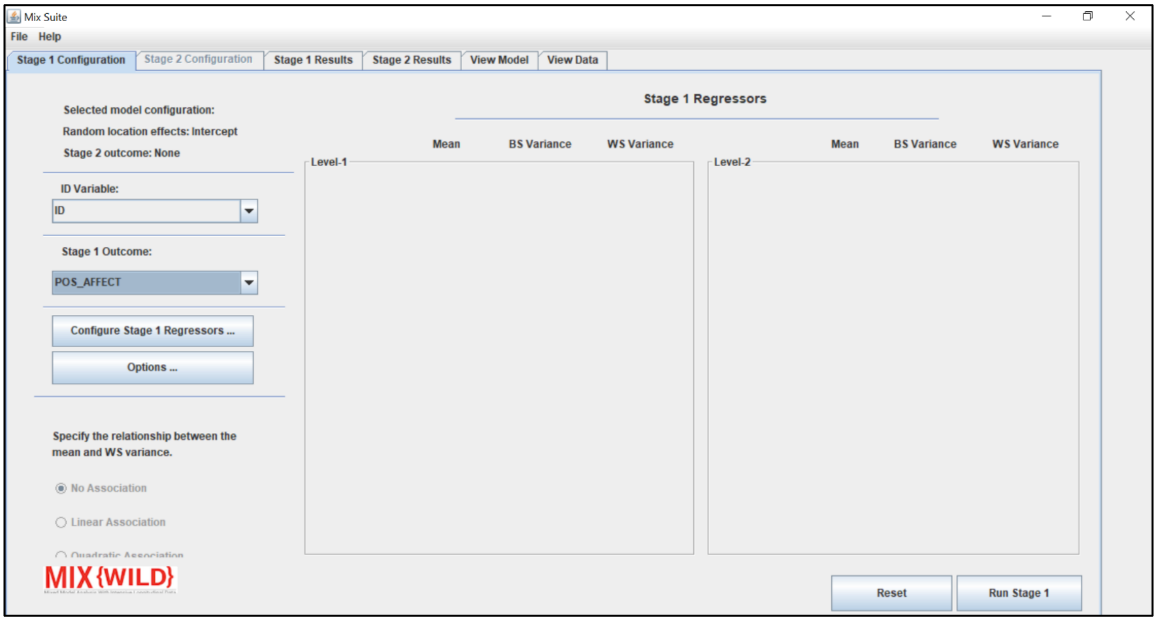

11. On the Stage 1 Configuration page, select your ID variable and positive affect (a time-varying variable) as your outcome variable.
12. By default, **"No Association"** is assumed between the mean and within-subject variance (only relevant for models with random scale). For random scale models, a linear or quadratic association is also possible.

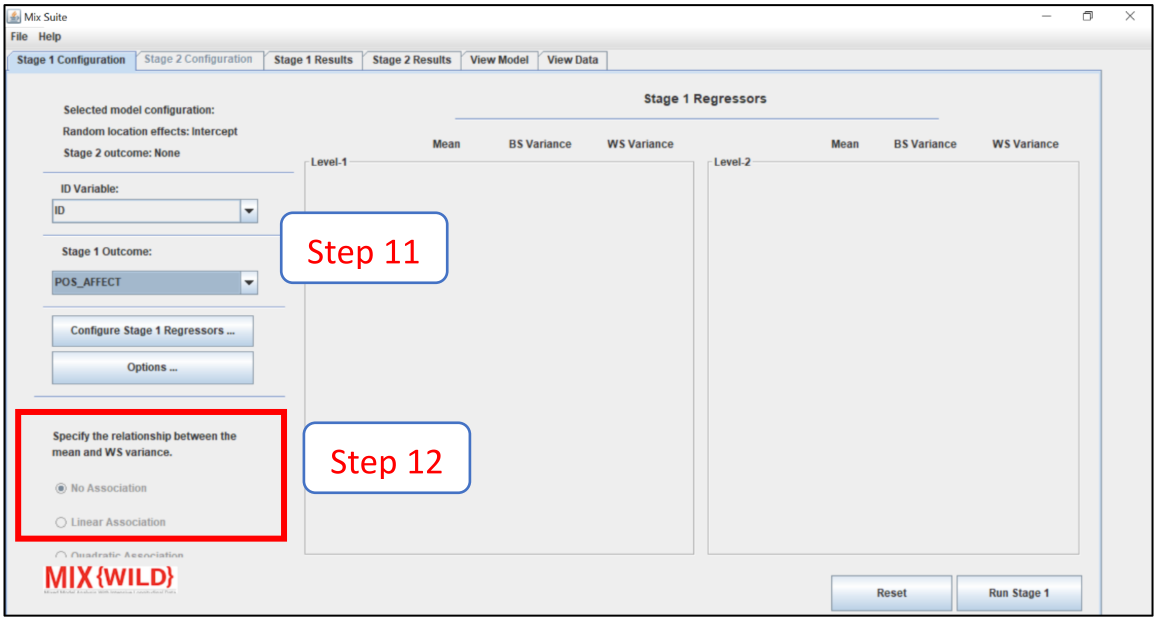

13. Click on **"Modify Stage 1 regressors"** to add other regressors.
14. Select and add time-varying regressor **"NEG_AFFECT"** and time-invariant regressor **"SEX"** into the corresponding boxes.

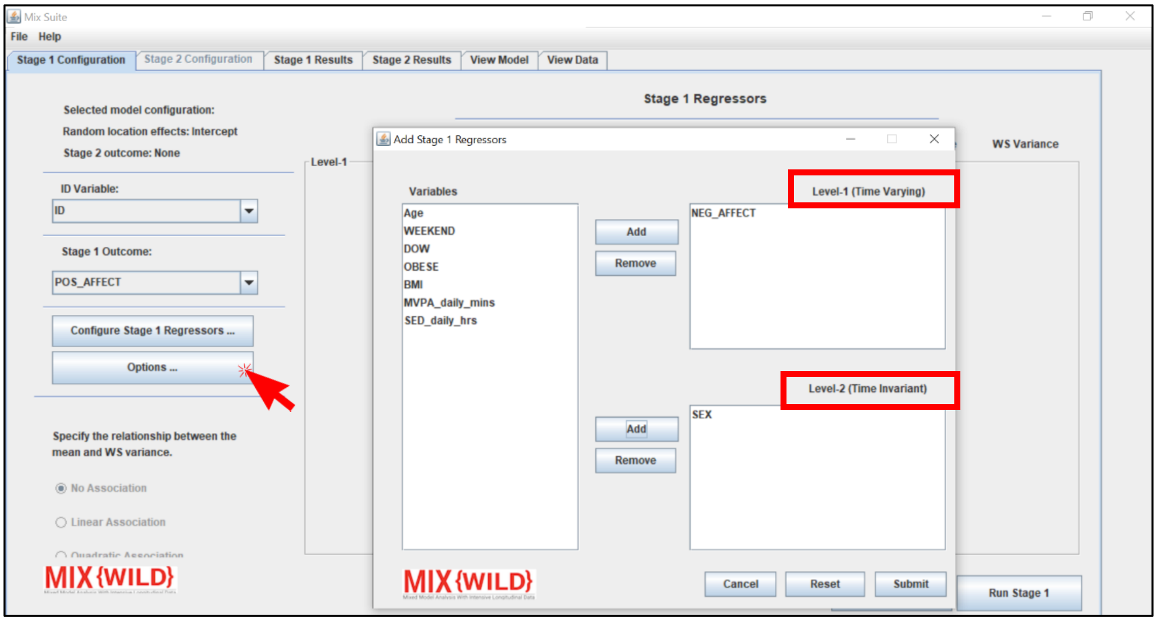

15. Select the box in the mean column to allow regressors to predict the mean level of outcome only.
16. Select **"Disaggregate"** for time-varying variable(s) in the mean column if the decomposition of the within-subject and between-subject effects in predicting the outcome is desired.

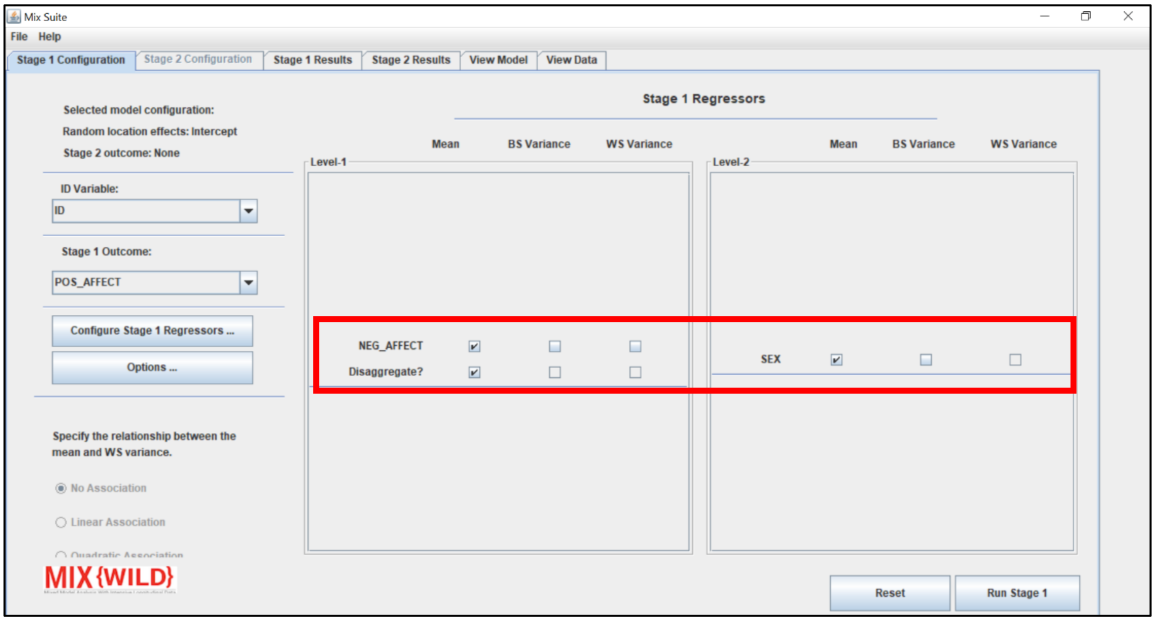

17. Click on **"Options"** to change other default settings if needed.
18. For models that do not include a random scale effect, removing subjects with no variance in outcome variable (positive affect in this example) is not necessary. Click on **"Submit'** to continue.

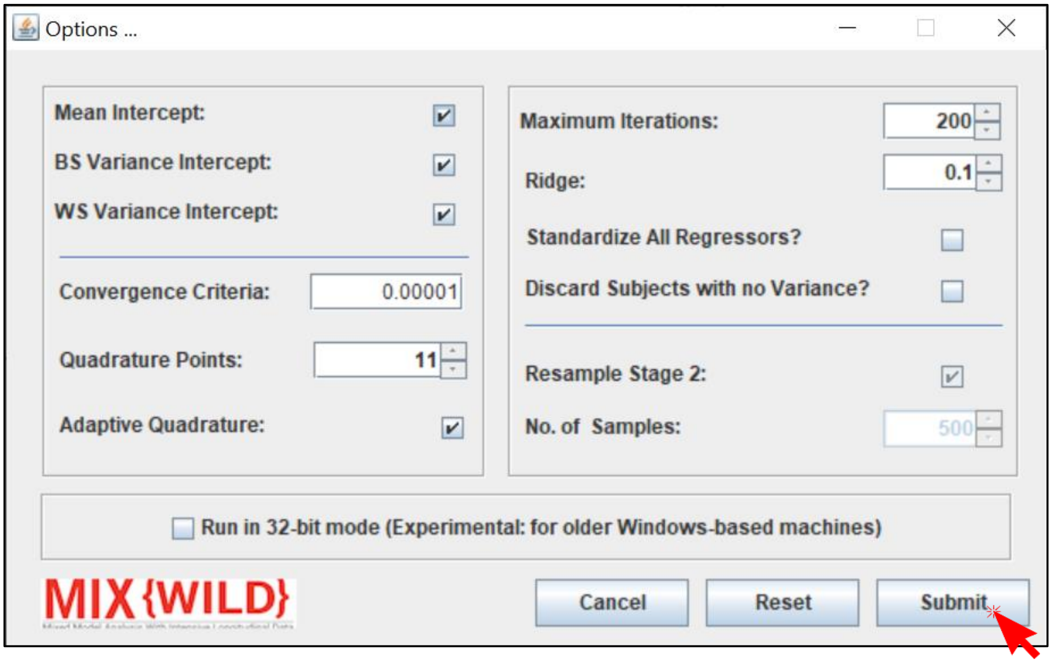

19. Verify your model specifications and click on **“Run Stage 1”** to generate the definition (Def) file. The definition file contains the syntax that instructs the program to estimate the specified model. You can save the definition file by clicking “Save Def File”.

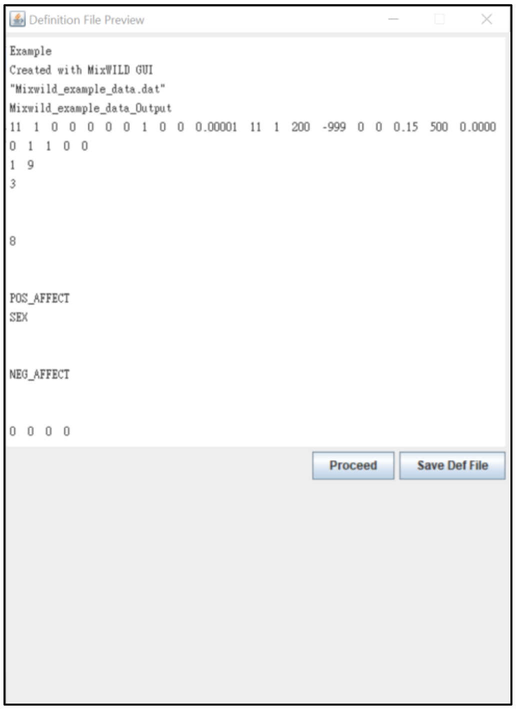

20. In the definition file, click on **“Proceed”** to run your model and generate model output files.
21. A window will appear while model estimation is in progress.

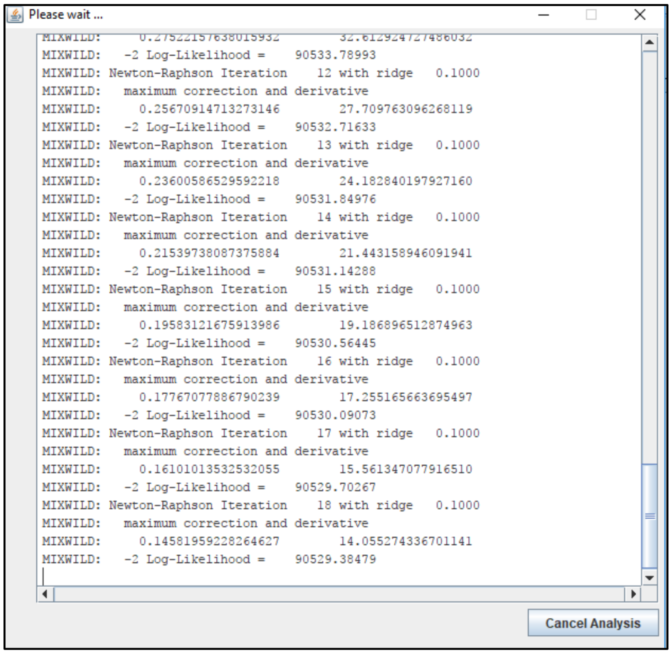

22. If the following warning message appears, it indicates that computational difficulties were encountered and prevented the model parameters from being estimated successfully. In this case, confirm the format of your dataset and your model specifications. Troubleshooting suggestions are listed in Appendix A.

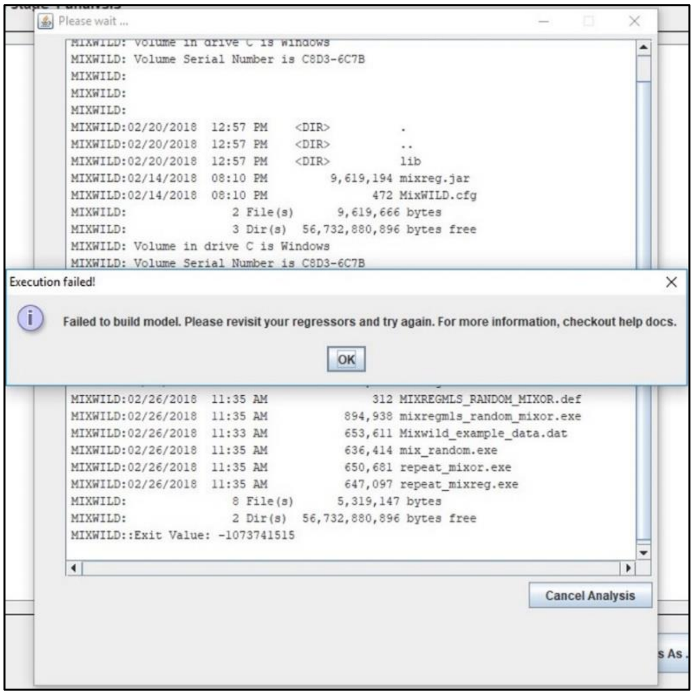

23. When the model analyzing is completed, the Stage 1 results can be seen by clicking the **"Stage 1 Results"** box.

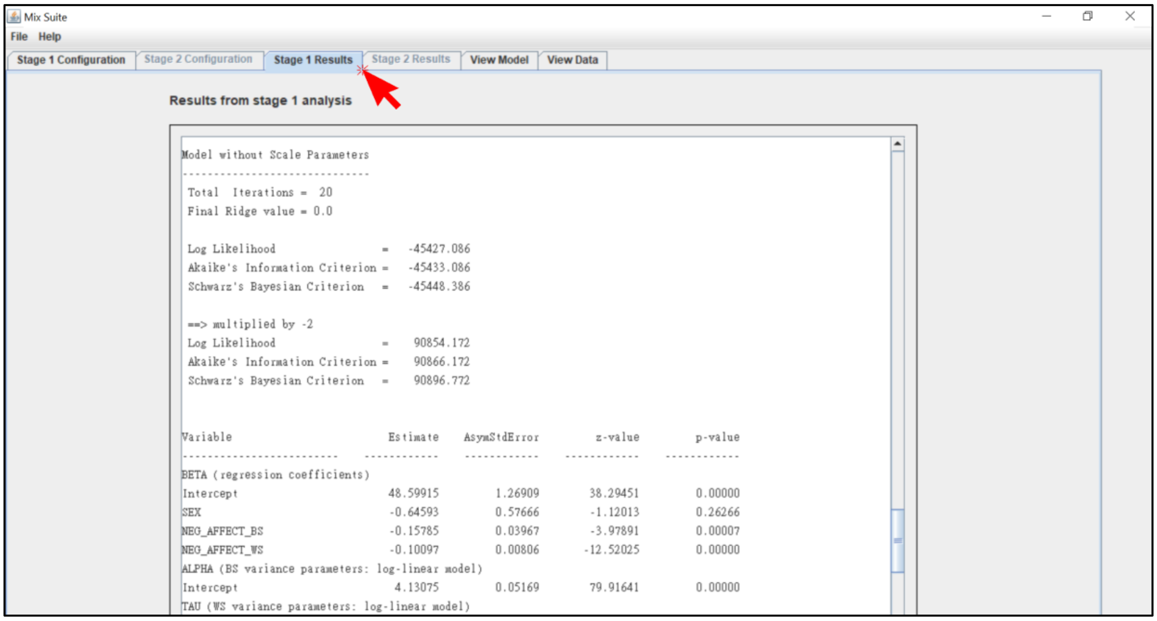

24. All files generated from the program can be found in a folder with the prefix MixWILD under the same directory of your dataset.

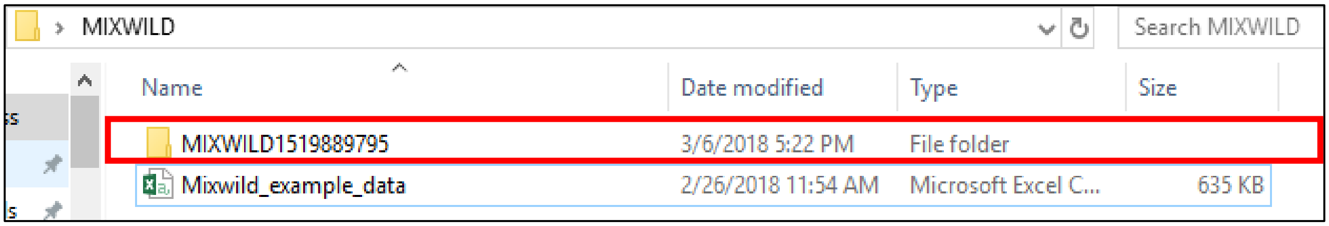

25. In the Output_1 txt file, you can find your results that are identical to the results found in the Stage 1 results box

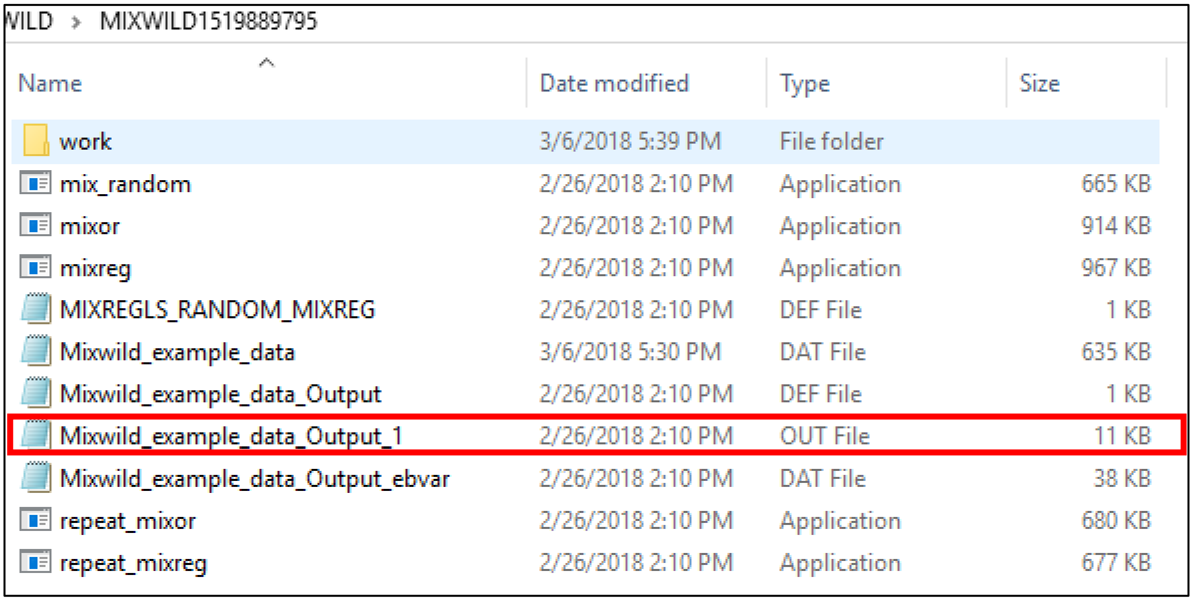

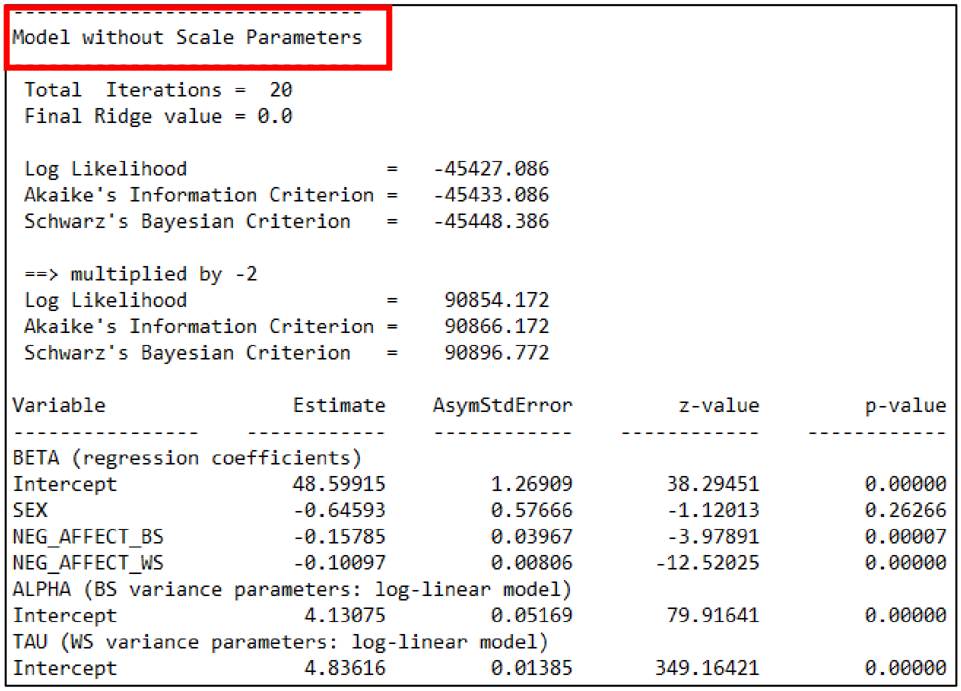
```
------------------------------
Model without Scale Parameters
------------------------------
 Total  Iterations =  20
 Final Ridge value = 0.0

 Log Likelihood                 =   -45427.086
 Akaike's Information Criterion =   -45433.086
 Schwarz's Bayesian Criterion   =   -45448.386

 ==> multiplied by -2
 Log Likelihood                 =    90854.172
 Akaike's Information Criterion =    90866.172
 Schwarz's Bayesian Criterion   =    90896.772

Variable                Estimate    AsymStdError         z-value         p-value
 ----------------    ------------    ------------    ------------    ------------
BETA (regression coefficients)
Intercept               48.59915         1.26909        38.29451         0.00000
SEX                     -0.64593         0.57666        -1.12013         0.26266
NEG_AFFECT_BS           -0.15785         0.03967        -3.97891         0.00007
NEG_AFFECT_WS           -0.10097         0.00806       -12.52025         0.00000
ALPHA (BS variance parameters: log-linear model)
Intercept                4.13075         0.05169        79.91641         0.00000
TAU (WS variance parameters: log-linear model)
Intercept                4.83616         0.01385       349.16421         0.00000
```

2.2. Brief interpretation of the multilevel model results

```
------------------------------
Model without Scale Parameters
------------------------------
 Total  Iterations =  20
 Final Ridge value = 0.0

 Log Likelihood                 =   -45427.086
 Akaike's Information Criterion =   -45433.086
 Schwarz's Bayesian Criterion   =   -45448.386

 ==> multiplied by -2
 Log Likelihood                 =    90854.172
 Akaike's Information Criterion =    90866.172
 Schwarz's Bayesian Criterion   =    90896.772

Variable                Estimate    AsymStdError         z-value         p-value
----------------    ------------    ------------    ------------    ------------
BETA (regression coefficients)
Intercept               48.59915         1.26909        38.29451         0.00000
SEX                     -0.64593         0.57666        -1.12013         0.26266
NEG_AFFECT_BS           -0.15785         0.03967        -3.97891         0.00007
NEG_AFFECT_WS           -0.10097         0.00806       -12.52025         0.00000
ALPHA (BS variance parameters: log-linear model)
Intercept                4.13075         0.05169        79.91641         0.00000
TAU (WS variance parameters: log-linear model)
Intercept                4.83616         0.01385       349.16421         0.00000
```

- Notice that the model results do not include effects on the BS or WS variances; as they were not specified earlier. Alpha and Tau intercepts are default parameters in the output table. These represent the BS and WS variances of positive affect, respectively, on the log scale.
- Results show that after controlling for sex, individuals who have higher mean negative affect have lower mean levels of positive affect (BS estimate = -0.1579, $p < .001$).
- When individuals have higher momentary negative affect than their own mean, they have lower momentary positive affect (WS estimate = -0.1010, $p < .001$).
- The BS variance in positive affect is reported as the Intercept estimate under Alpha, estimated as exp(4.13) = 62.18. The WS variance in positive affect is reported as the Intercept estimate under Tau, estimated as exp(4.84) = 126.47. Thus, the intraclass correlation (ICC) for positive affect is estimated as 62.18 / (62.18 + 126.47) = 0.33.

3. Example 2: Running Mixregls - Linear Regression model in MixWILD

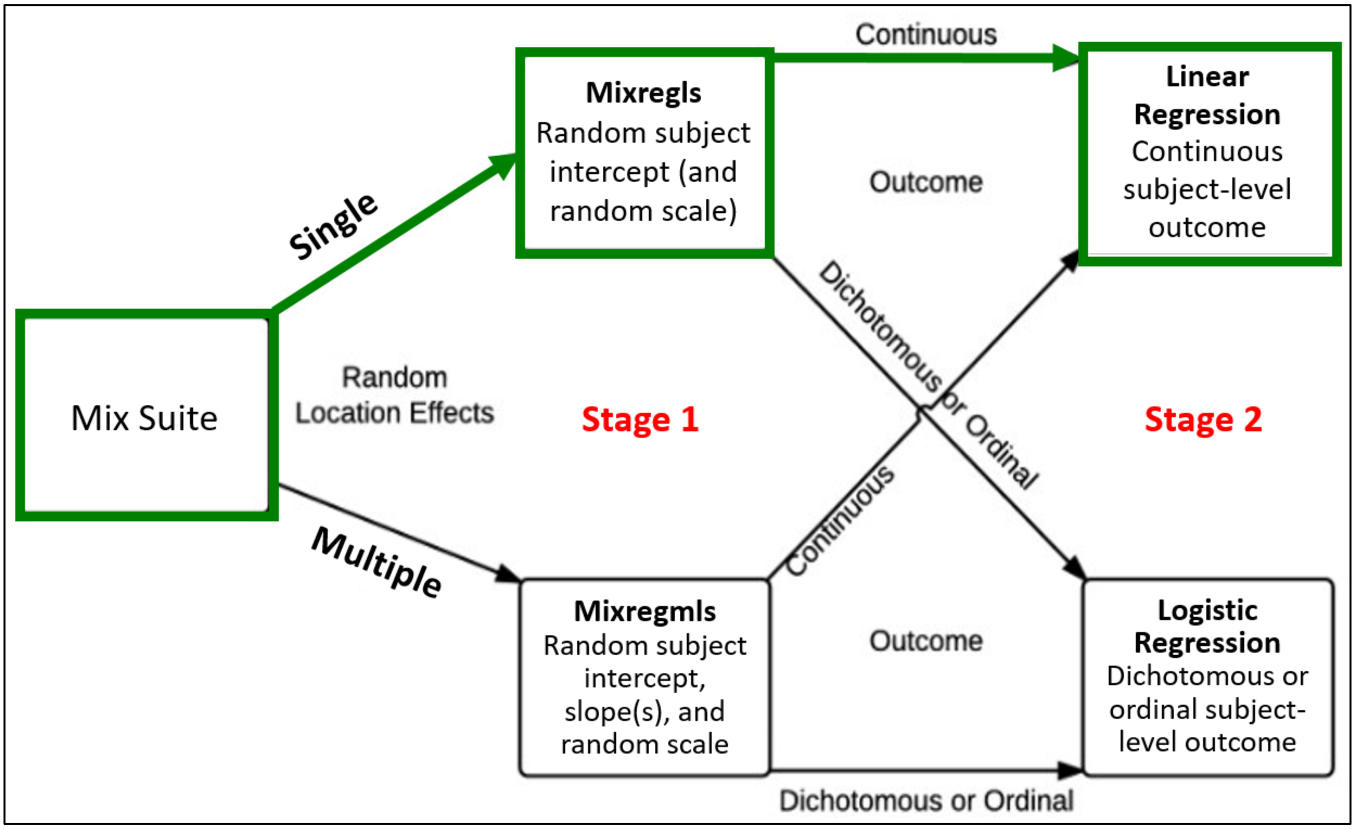

Example research questions for Mixregls-Linear Regression model

- Examine whether subject-level mean (i.e., random intercept or within-person mean) and subject level variance (i.e., random scale or degree of within-person / intraindividual variability) of momentary positive affect (within-subject, continuous, time-varying variable) predicts subject-level average sedentary hours per day (between-subject, continuous, time-invariant variable), after controlling for sex and day of week (DOW).
- Examine whether participants' age (between-subject, time-invariant variable) ***moderates*** the associations between participants' mean level (i.e., random intercept) and (i.e., random scale) variance in momentary positive affect in predicting subject-level average hours per day of sedentary behavior (between-subject, continuous, time-invariant variable), after controlling for sex and day of week (DOW).

Stage 1 outcome variable: Positive affect (time-varying)
Stage 1 regressors: Day of week (time-varying); Sex (time-invariant)
Stage 2 outcome variable: Average sedentary hours per day (time-invariant)
Stage 2 regressors: Age (time-invariant); Age x Random Intercept interaction; Age x Random Scale interaction

3.1. Step-by-step instructions on running Mixregls - Linear Regression model in MixWILD

1. Double-click on the MixWILD icon to open the main window.
2. Click on **"File"** and then select **"New Model"** (or use keyboard shortcut Ctrl + N).

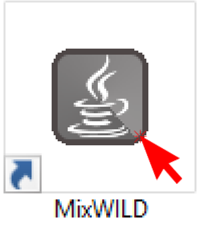

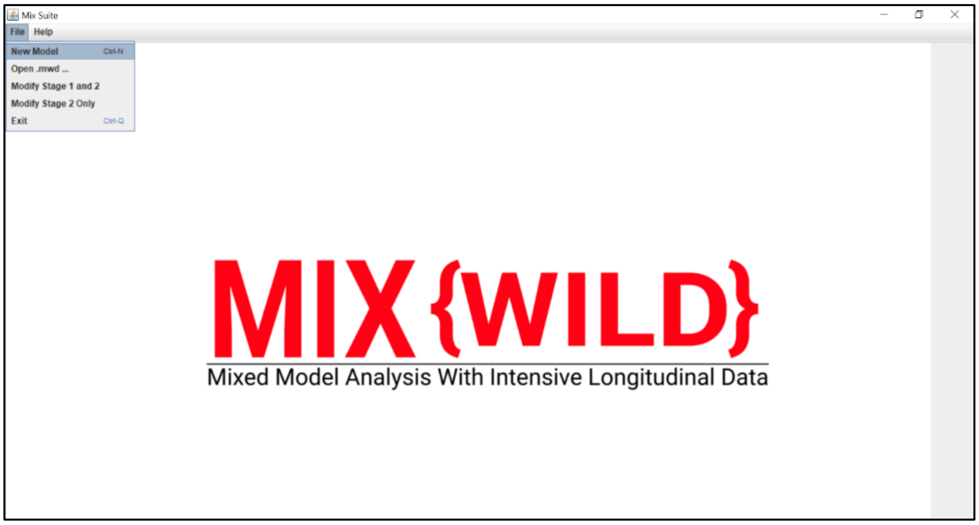

3. Click on **"Instructions"** to make sure your data are in the correct format.

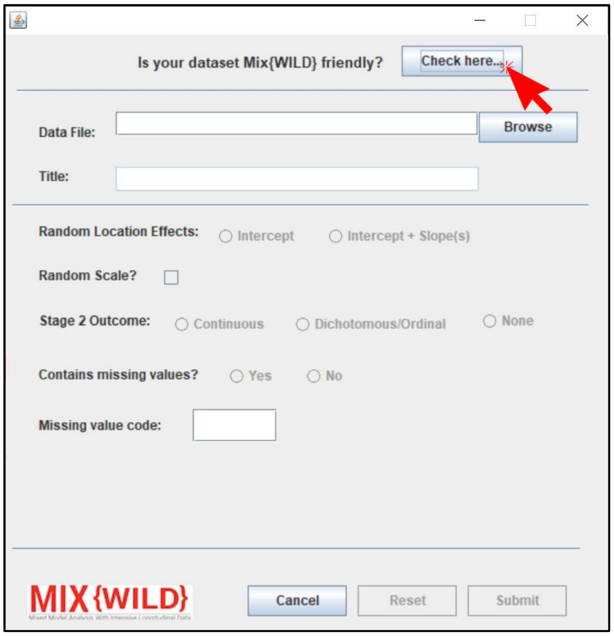

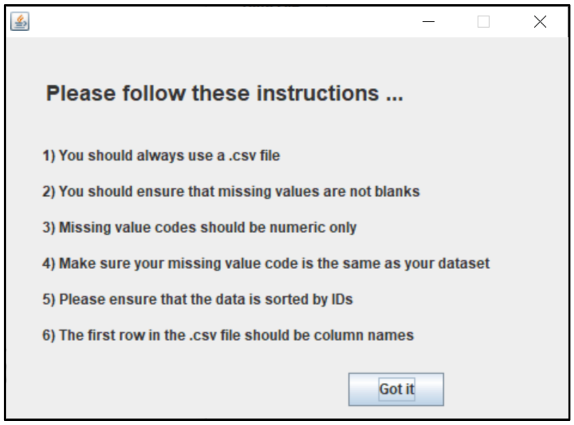

4. Click on **“Browse”** to select the location of your data file (in .csv) and then click **“Open”**.

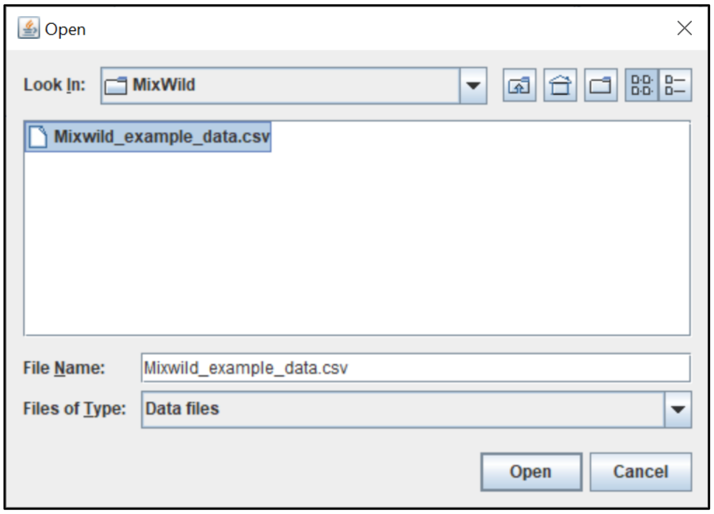

5. Click on **“View Data”** to preview your data file to verify your data and format are correct.

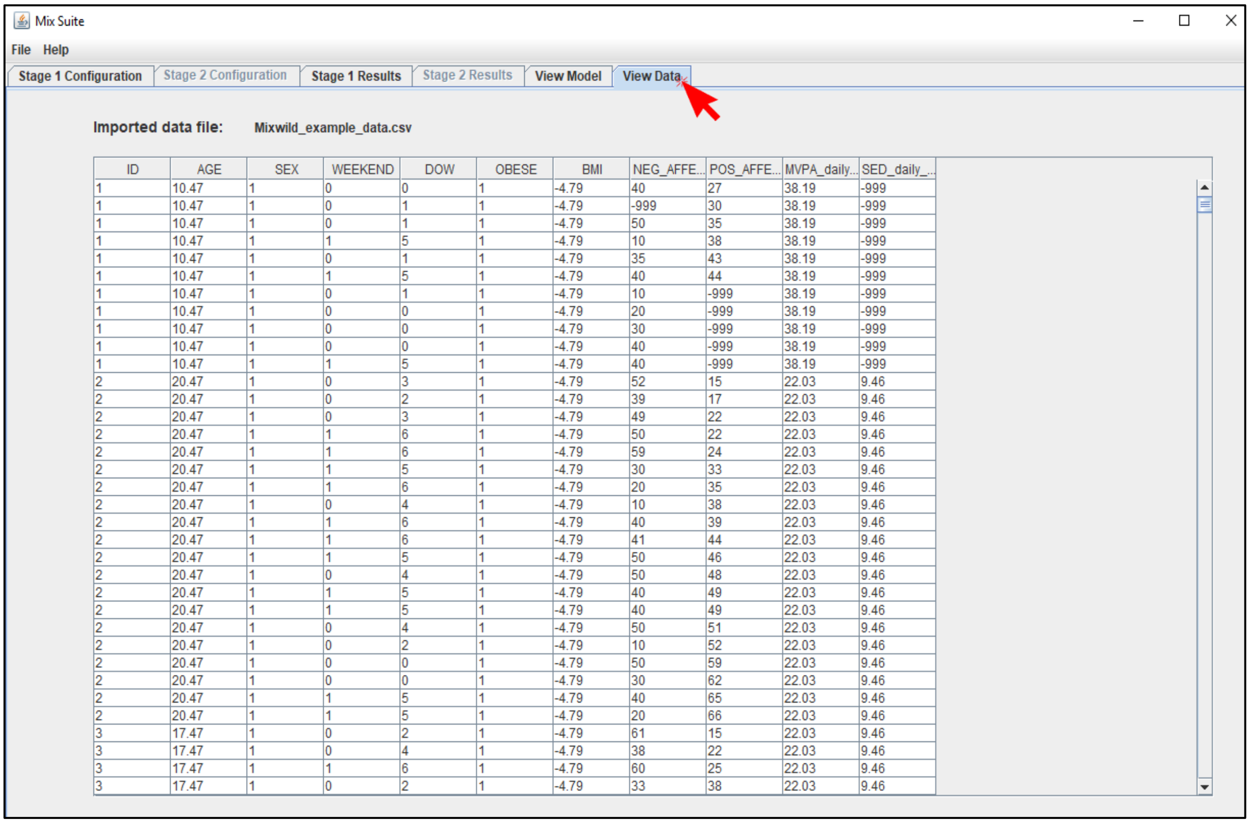

ID	AGE	SEX	WEEKEND	DOW	OBESE	BMI	NEG_AFFE...	POS_AFFE...	MVPA_daily...	SED_daily_...
1	10.47	1	0	0	1	-4.79	40	27	38.19	-999
1	10.47	1	0	1	1	-4.79	-999	30	38.19	-999
1	10.47	1	0	1	1	-4.79	50	35	38.19	-999
1	10.47	1	1	5	1	-4.79	10	38	38.19	-999
1	10.47	1	0	1	1	-4.79	35	43	38.19	-999
1	10.47	1	1	5	1	-4.79	40	44	38.19	-999
1	10.47	1	0	1	1	-4.79	10	-999	38.19	-999
1	10.47	1	0	0	1	-4.79	20	-999	38.19	-999
1	10.47	1	0	0	1	-4.79	30	-999	38.19	-999
1	10.47	1	0	0	1	-4.79	40	-999	38.19	-999
1	10.47	1	1	5	1	-4.79	40	-999	38.19	-999
2	20.47	1	0	3	1	-4.79	52	15	22.03	9.46
2	20.47	1	0	2	1	-4.79	39	17	22.03	9.46
2	20.47	1	0	3	1	-4.79	49	22	22.03	9.46
2	20.47	1	1	6	1	-4.79	50	22	22.03	9.46
2	20.47	1	1	6	1	-4.79	59	24	22.03	9.46
2	20.47	1	1	5	1	-4.79	30	33	22.03	9.46
2	20.47	1	1	6	1	-4.79	20	35	22.03	9.46
2	20.47	1	0	4	1	-4.79	10	38	22.03	9.46
2	20.47	1	1	6	1	-4.79	40	39	22.03	9.46
2	20.47	1	1	6	1	-4.79	41	44	22.03	9.46
2	20.47	1	1	5	1	-4.79	50	46	22.03	9.46
2	20.47	1	0	4	1	-4.79	50	48	22.03	9.46
2	20.47	1	1	5	1	-4.79	40	49	22.03	9.46
2	20.47	1	1	5	1	-4.79	40	49	22.03	9.46
2	20.47	1	0	4	1	-4.79	50	51	22.03	9.46
2	20.47	1	0	2	1	-4.79	10	52	22.03	9.46
2	20.47	1	0	0	1	-4.79	50	59	22.03	9.46
2	20.47	1	0	0	1	-4.79	30	62	22.03	9.46
2	20.47	1	1	5	1	-4.79	40	65	22.03	9.46
2	20.47	1	1	5	1	-4.79	20	66	22.03	9.46
3	17.47	1	0	2	1	-4.79	61	15	22.03	9.46
3	17.47	1	0	4	1	-4.79	38	22	22.03	9.46
3	17.47	1	1	6	1	-4.79	60	25	22.03	9.46
3	17.47	1	0	2	1	-4.79	33	38	22.03	9.46

6. Add title to your output files. This title name is later displayed in your output files.
7. Select **“Intercept”** from Random Location Effects specification for the subject-level mean and check **“Random Scale”** for subject-level variability
8. Select **“Continuous”** for the Stage 2 outcome.
9. Click on missing values if there are any in your dataset; specify the missing value code in the box (i.e., -999 in the example dataset).

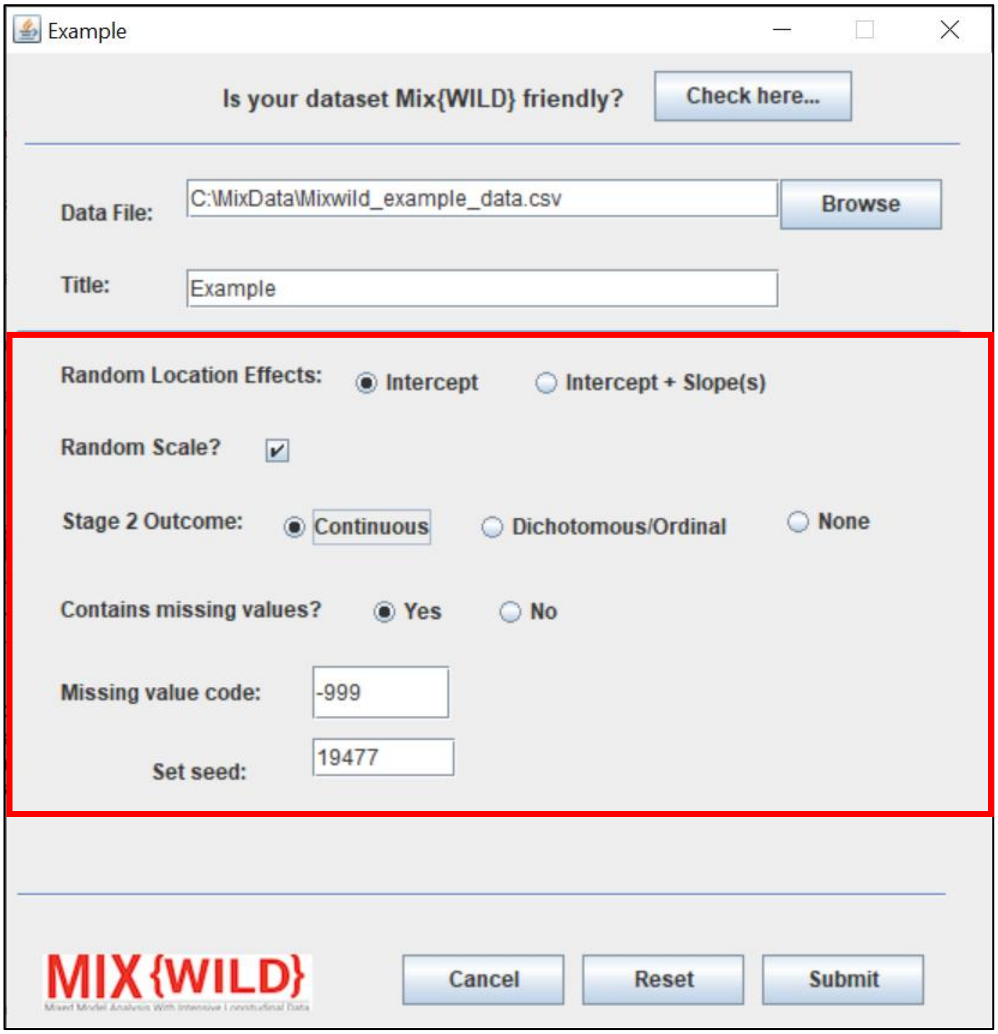

10. After you submit, the interface will take you to the page that enables you to configure your Stage 1 model.
11. On the Stage 1 configuration page, select your ID variable and positive affect as your Stage 1 outcome variable.
12. Specify the association between mean and within- subject (WS) variance of the outcome variable, which is the association of the random location and random scale effects. **“Linear Association”** is selected for the following example. For random scale models, a quadratic association is also possible.

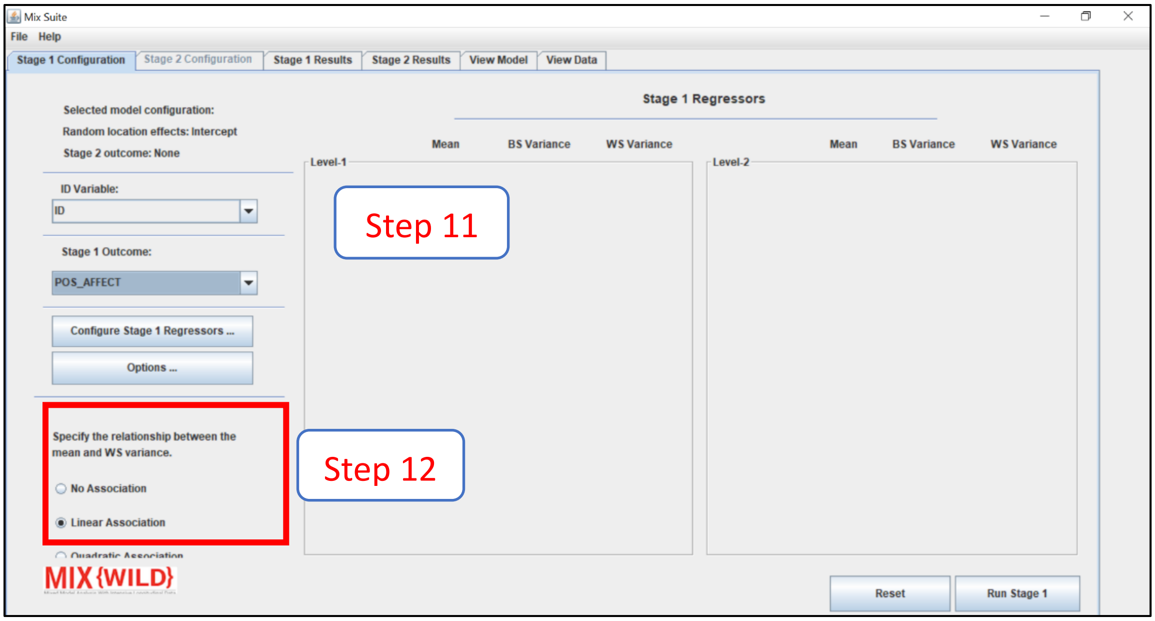

13. Click on **"Modify Stage 1 regressors"** to select other regressors.
14. Select and add time-varying regressor **"DOW"** and time-invariant regressor **"SEX"**. (Note that for demonstration, day of the week (DOW) is treated as a linear regressor of positive affect. The user might want to instead treat this as a factor in the model by creating 6 dummy codes for the 7 days.

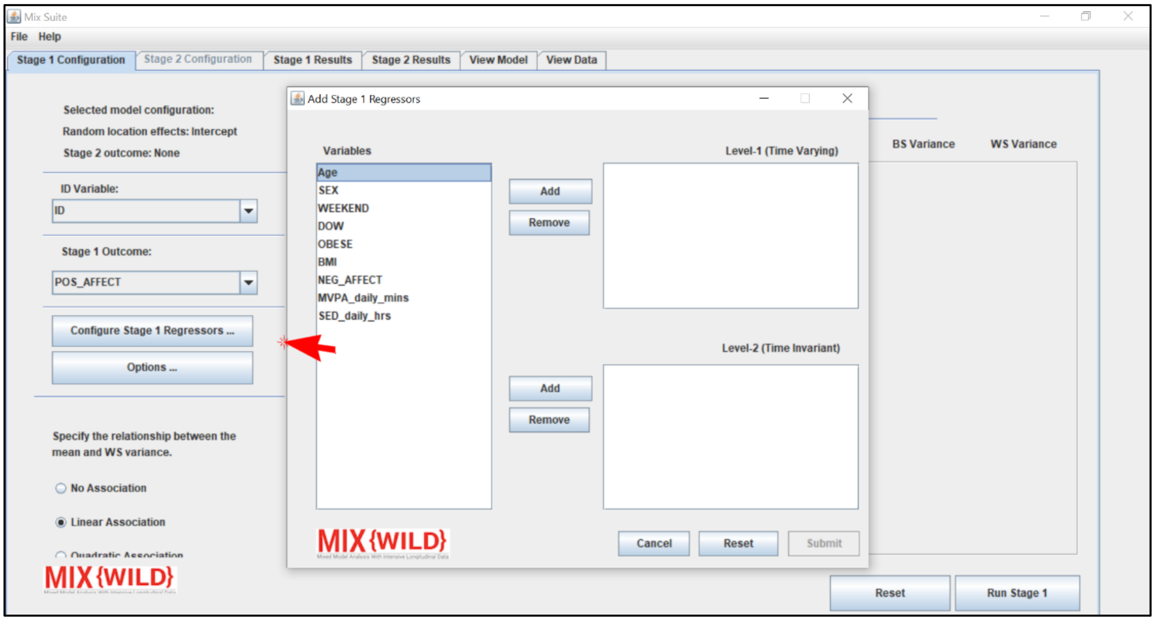

15. Select to allow Stage 1 regressors to predict mean, between-subject variance, and/or within-subject variance of Stage 1 outcome.

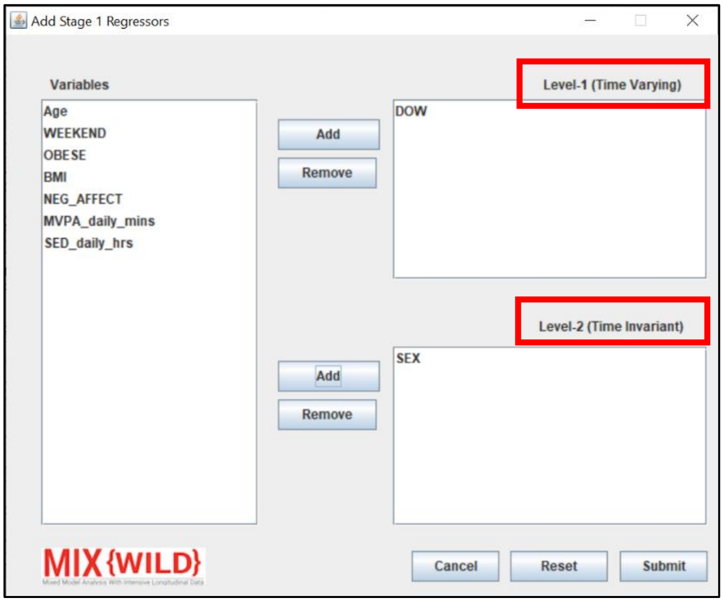

15. Select the boxes in the mean column, BS Variance column, and WS Variance column to allow Stage 1 regressors to predict the mean, between-subject variance, and/or within-subject variance of Stage 1 outcome, respectively. Day of week is select to predict the mean.
16. Select **"Disaggregate"** for each of the time-varying variable(s) for which decomposition of the within-subject and between-subject effects in predicting Stage 1 outcome is desired. Day of week is not aggregated in this example.

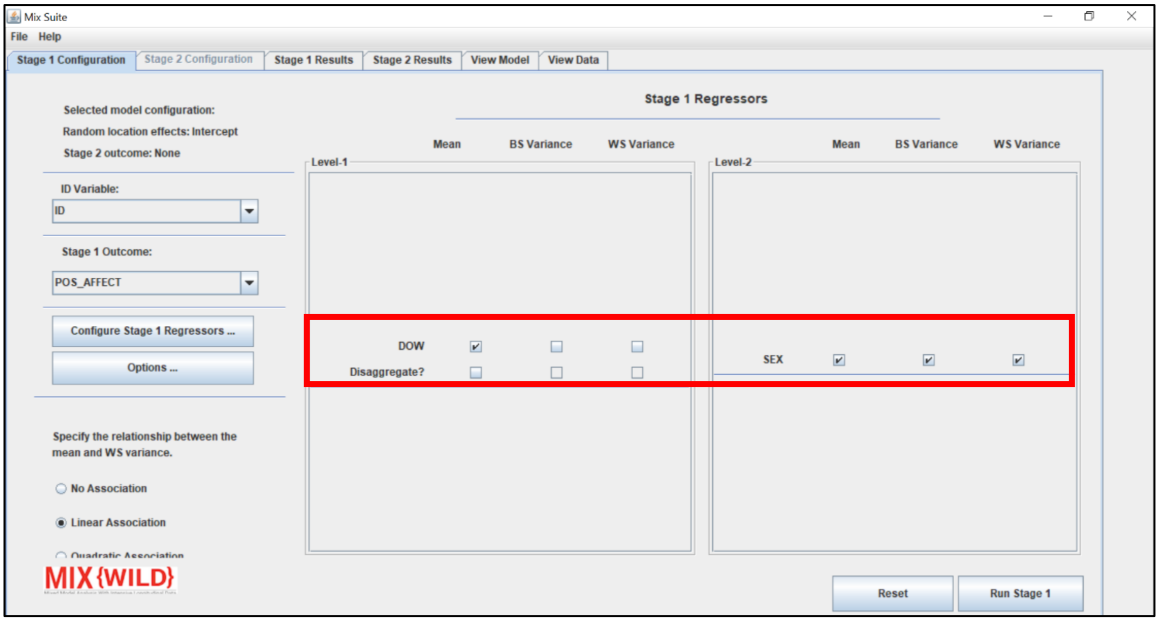

17. Click on **"Options"** to change other default settings if needed.
18. In most cases, Mean, BS and WS intercept should remain checked. Also, uncheck the **"Discard Subjects"** option to use all data for analysis.
19. By default, the estimated random effects of the Stage 1 analysis (i.e., random location and scale effects) are resampled 500 times in the Stage 2 analysis. Resampling is necessary because the random effects are estimated quantities that are entered as regressors in the Stage 2 model. (refer to the supplemental documents for further description of Options settings)

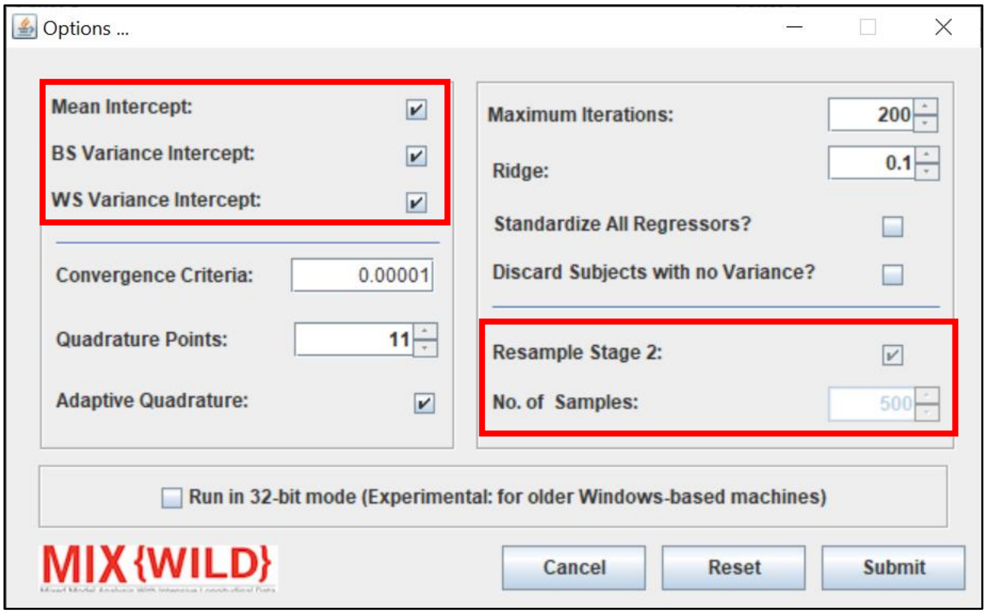

20. Click on **"Submit"** and then go to **"Configure Stage 2"**.

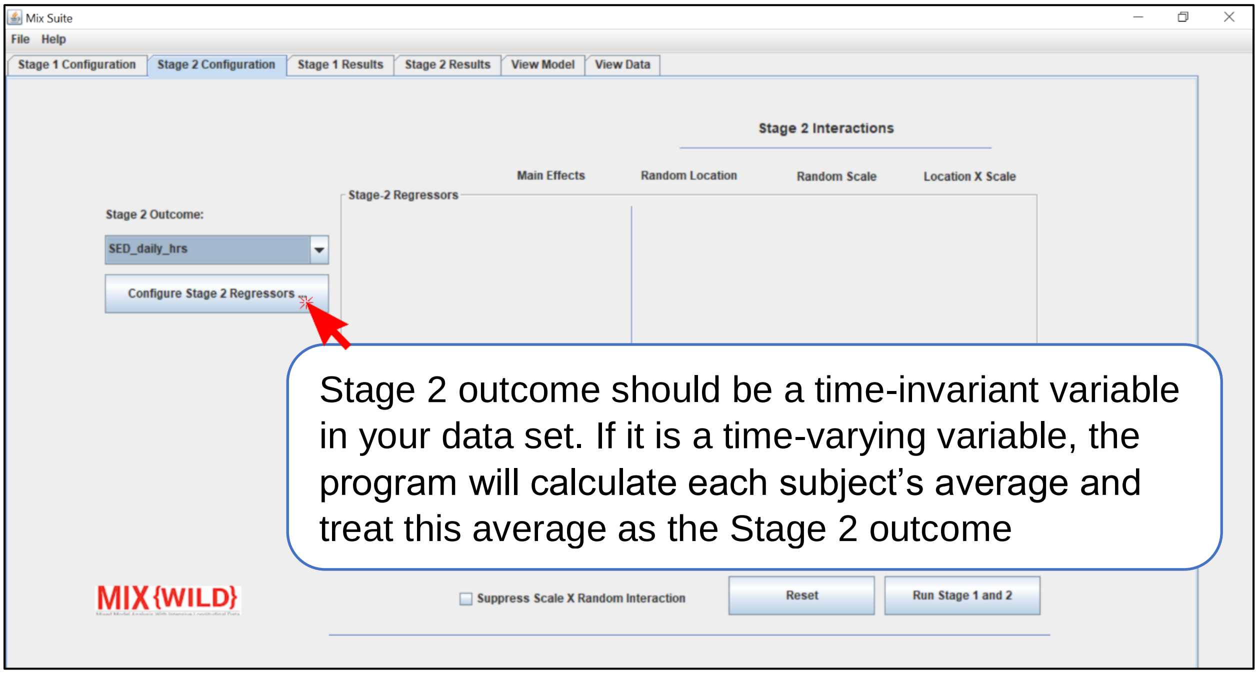

21. Select **"SED_daily_hours"** as Stage 2 outcome variable.
22. Add Stage 2 regressor(s) into the model. Stage 2 regressor(s) are also generally time-invariant variable(s), however if they are time-varying then the program will calculate subject averages of those variables. Click on **"Submit"** when finished.

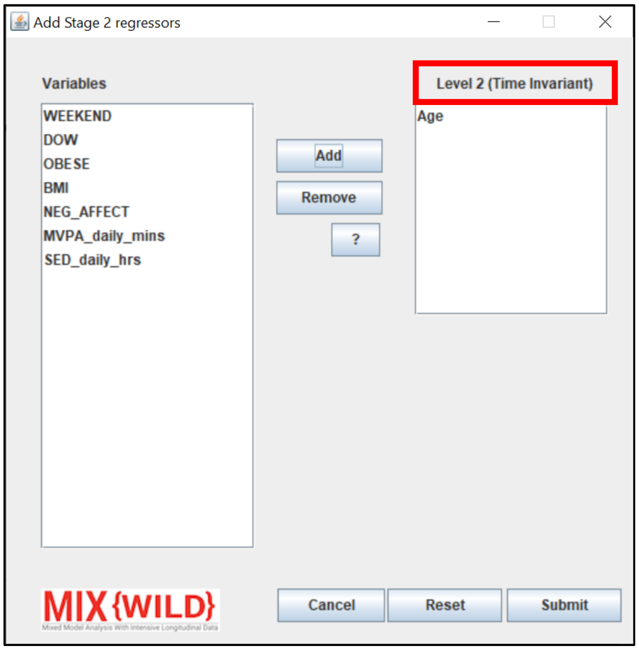

23. Select to add Stage 2 main effect(s) and interaction effect(s) with Stage 1 random effects as regressors. For example, by clicking the **"Location x Scale"** box, a 3-way interaction (AGE x Random location x Random scale) will be included to predict the Stage 2 outcome. This Location x Scale interaction option is enabled only if main effects are selected.

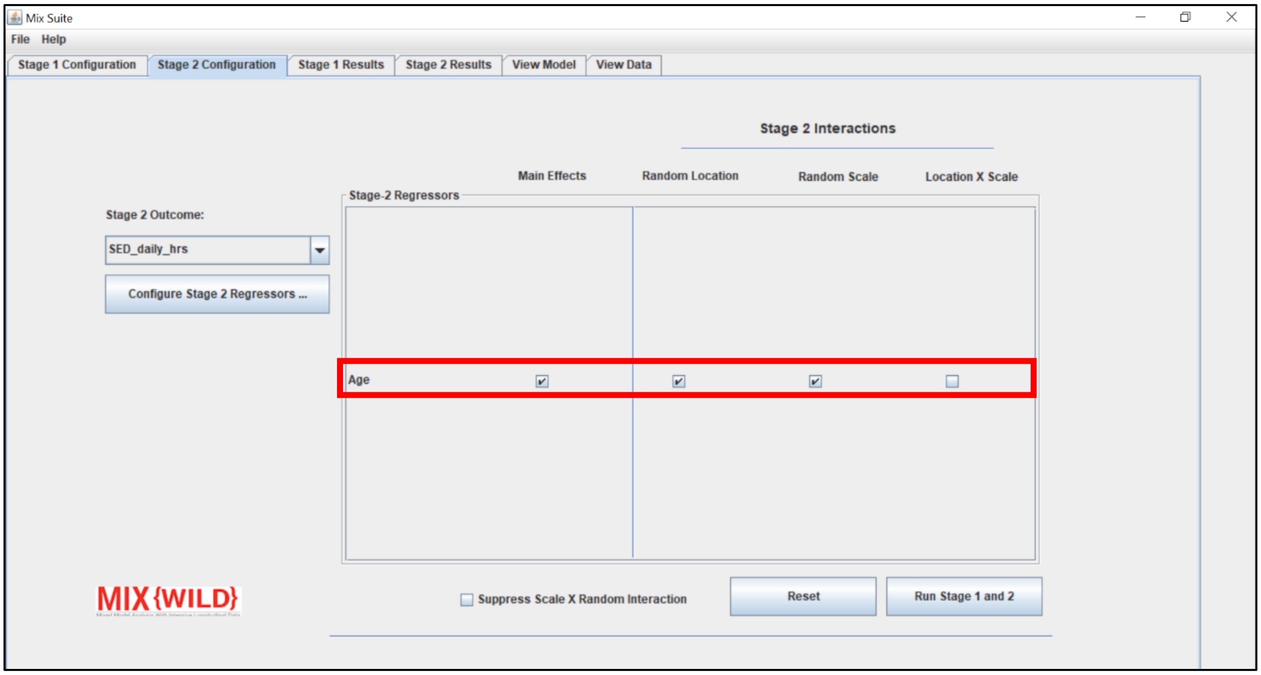

24. Click on Stage 1 and Stage 2 configurations to verify your model. Click on **"Run Stage 1 and 2"** to generate the definition file. The definition file contains the syntax that instructs the program to estimate the specified model.

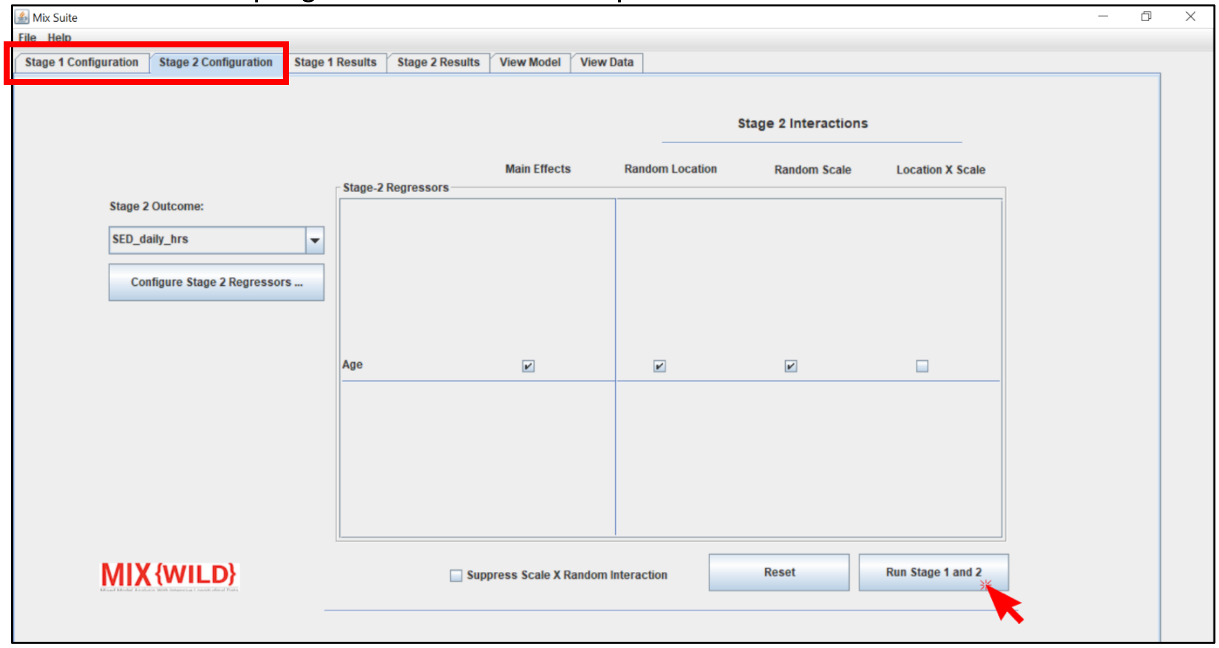

25. In the definition file, click on **"Proceed"** to run your model and generate model output files. If you see a field with the text **"null"**, it means that there is a mistake in your model specifications that should be fixed.

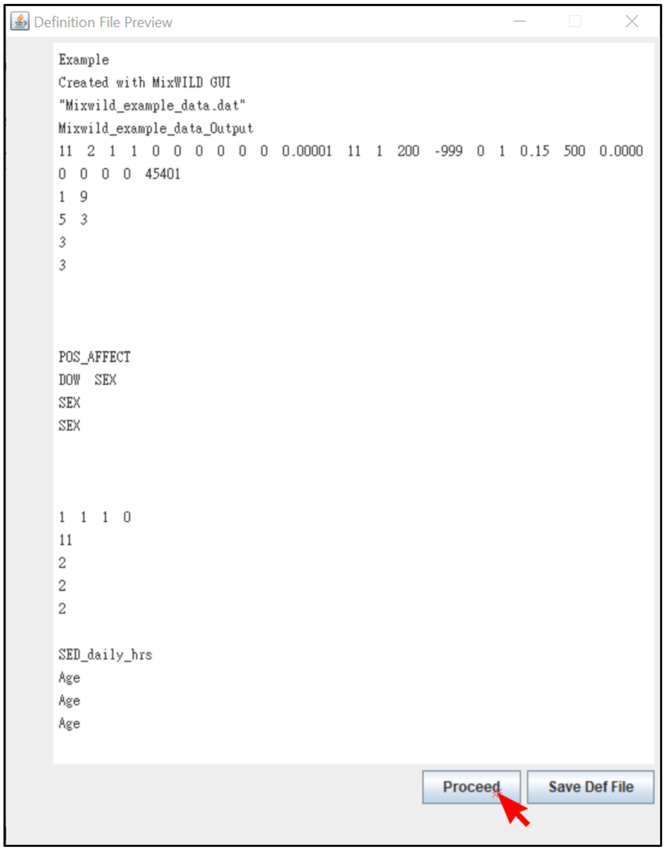

26. A window will appear while model estimation is in progress. The time for generating the final output depends on the dataset size and complication of your specified model. If your Stage 1 model has many regressors or if you select a large number for resampling, the run time could range up to 5 to 10 minutes.

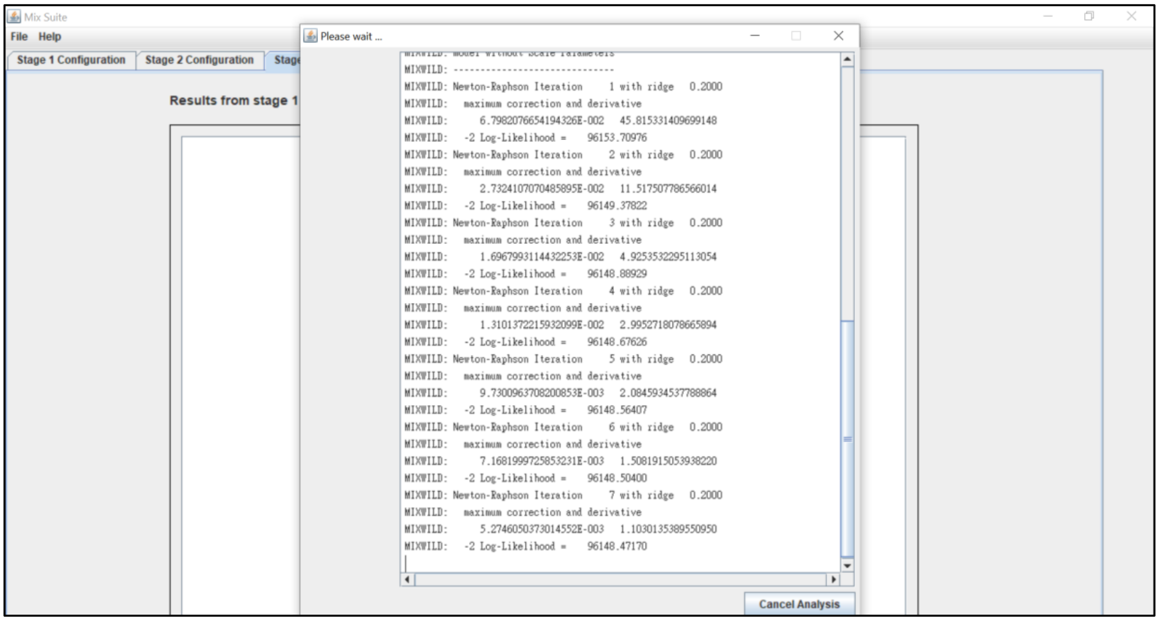

27. If the following warning message appears, it indicates that computational difficulties were encountered that prevented the model parameters from being estimated successfully. In this case, double-check the format of your dataset and your model specifications. Some suggestions for steps to take are listed in Appendix A.

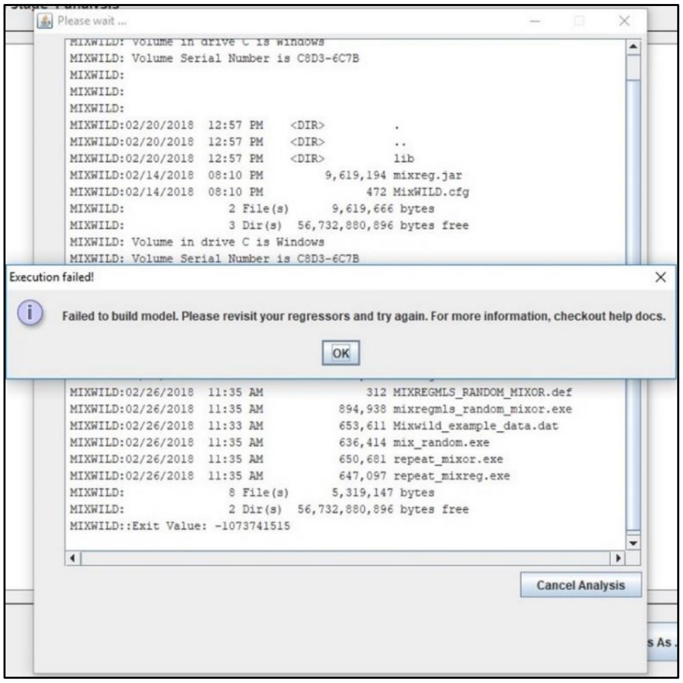

28. When estimation is completed, the Stage 1 and Stage 2 results can be seen by clicking the Stage 1 and Stage 2 Results boxes, respectively.

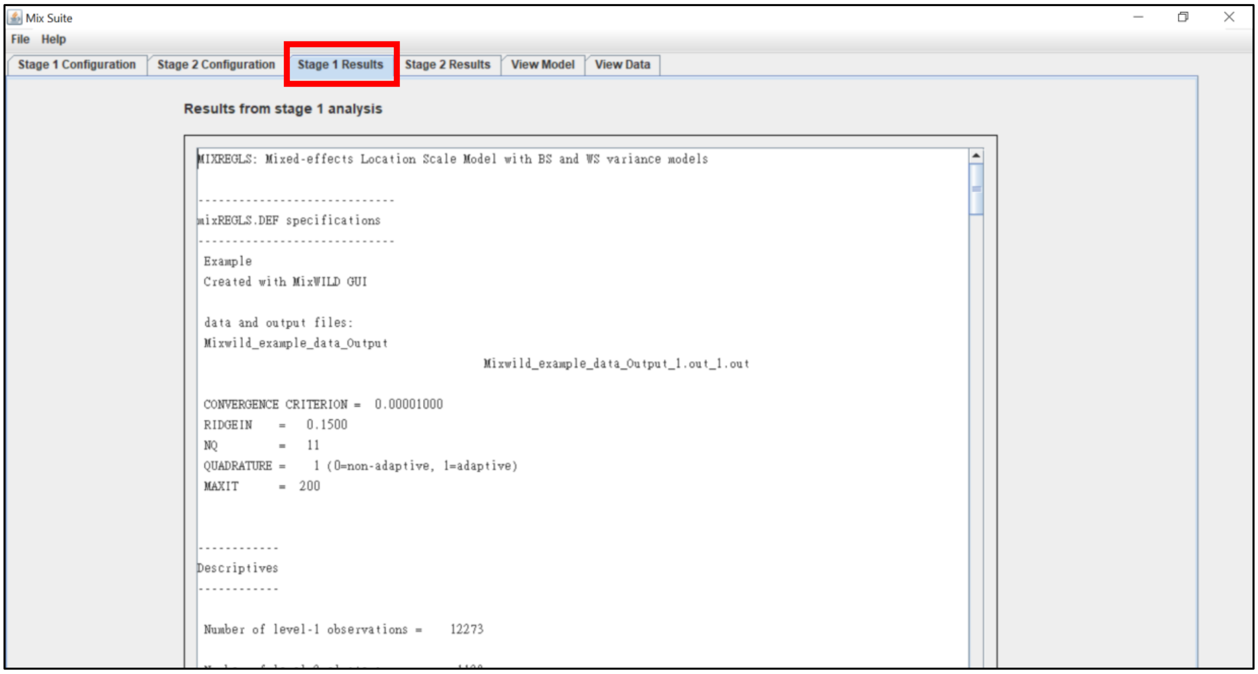

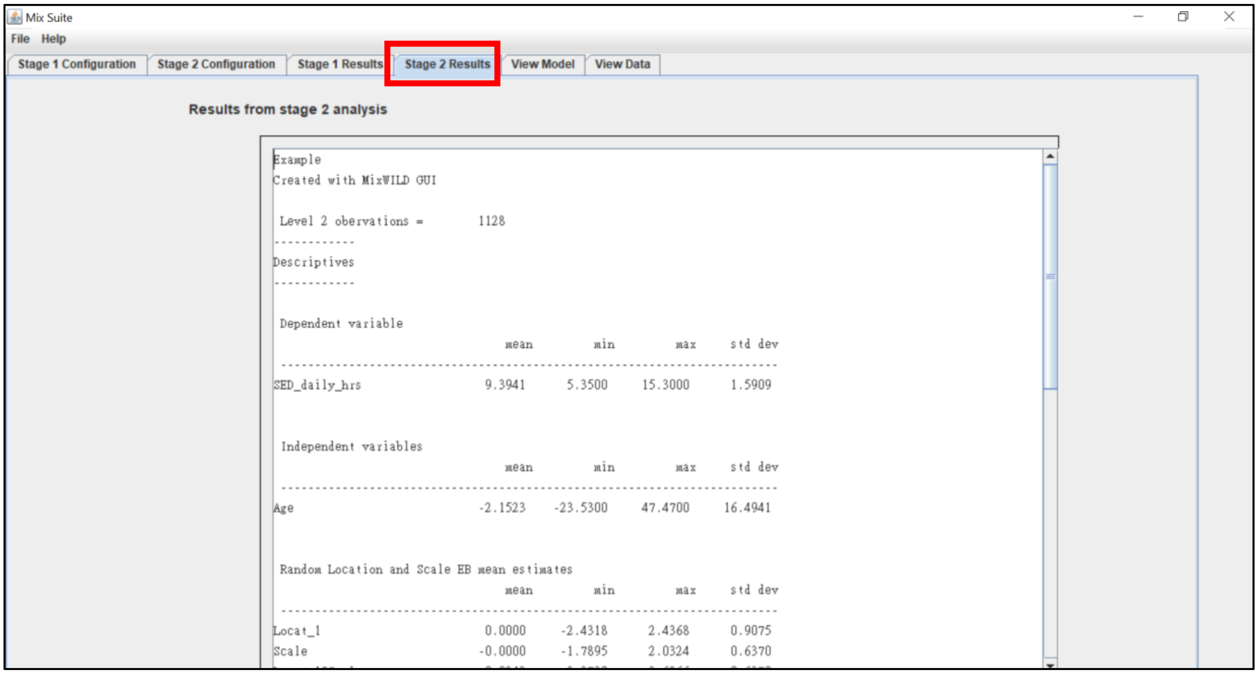

29. All files generated from the program can be found in a folder with the prefix MixWILD under the same directory of your dataset.

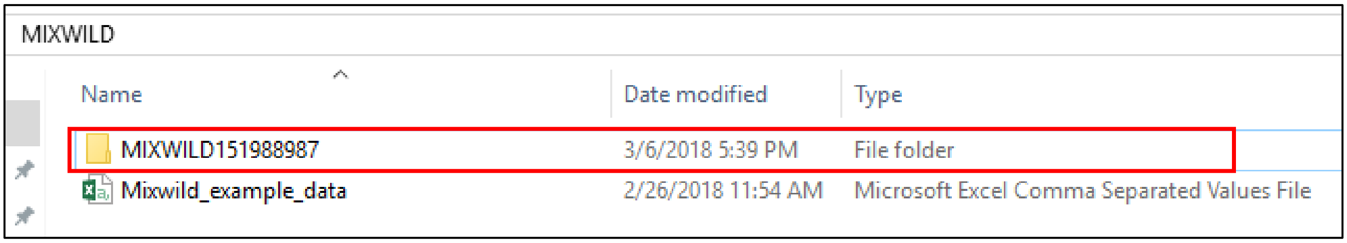

30. **The OUT files with suffix_1 and _2 are the results that are identical in the Stage 1 and Stage 2 boxes in the MixWILD program.**

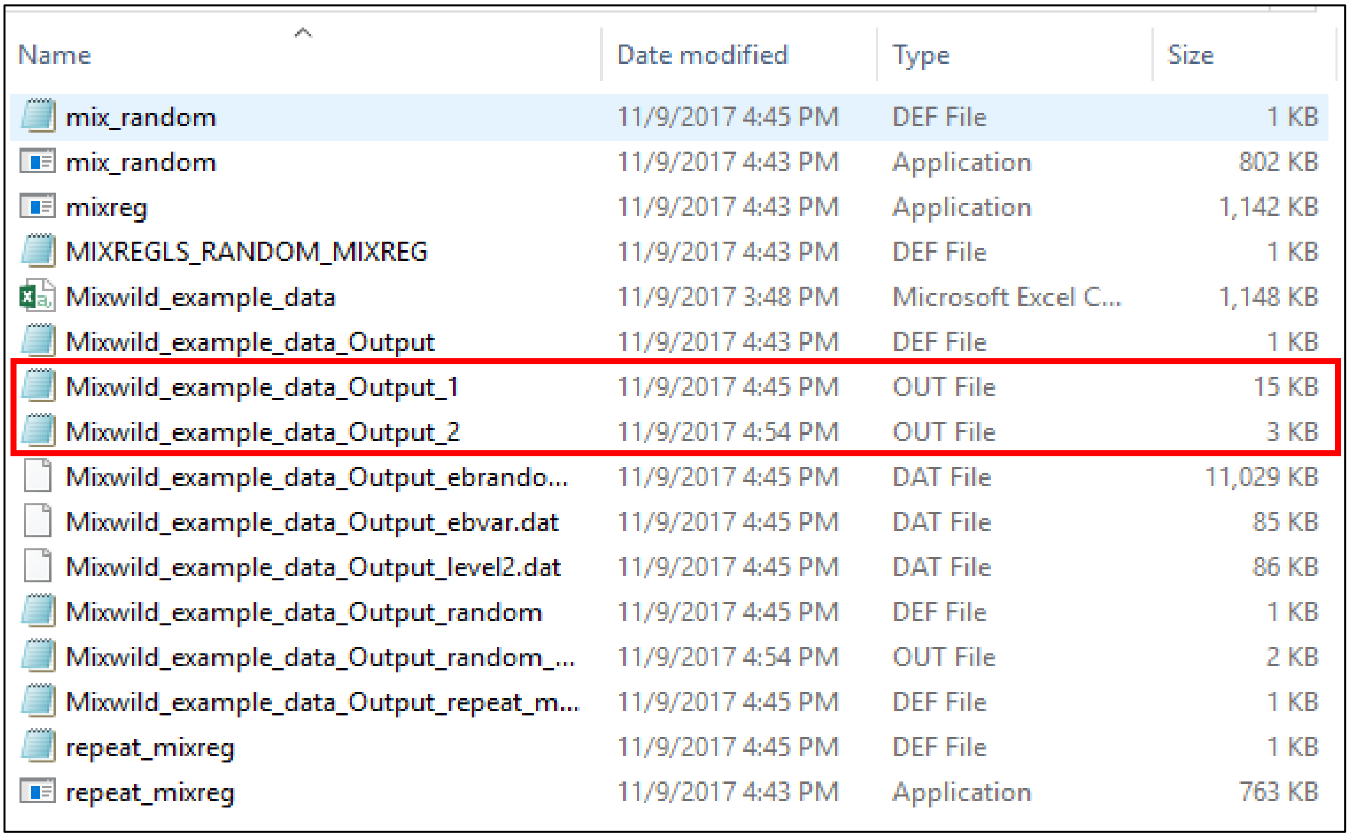

31. The OUT files with suffix_1 contains the results from Stage 1 model.
32. There are three sets of submodel results in output_1: the first submodel does not include scale parameters, the second submodel includes scale parameters but not random scale parameters, and the third submodel includes both the scale and the random scale parameters.
33. A brief description of the results from the third submodel will be provided in the following section.

```
------------------------------
Model without Scale Parameters
------------------------------
 Total  Iterations =  15
 Final Ridge value = 0.0

 Log Likelihood                 =   -48074.154
 Akaike's Information Criterion =   -48080.154
 Schwarz's Bayesian Criterion   =   -48095.238

 ==> multiplied by -2
 Log Likelihood                 =    96148.308
 Akaike's Information Criterion =    96160.308
 Schwarz's Bayesian Criterion   =    96190.477

Variable                 Estimate     AsymStdError        z-value        p-value
----------------     ------------     ------------   ------------   ------------
BETA (regression coefficients)
Intercept                42.57495          0.55102       77.26612        0.00000
DOW                       0.39721          0.04749        8.36346        0.00000
SEX                      -0.72502          0.60446       -1.19945        0.23035
ALPHA (BS variance parameters: log-linear model)
Intercept                 4.29050          0.09491       45.20633        0.00000
SEX                      -0.20952          0.11272       -1.85881        0.06305
TAU (WS variance parameters: log-linear model)
Intercept                 4.83228          0.01339      360.98308        0.00000
```

```
Model WITH Scale Parameters
---------------------------
 Total  Iterations =  19
 Final Ridge value = 0.0

 Log Likelihood                 =   -48071.771
 Akaike's Information Criterion =   -48078.771
 Schwarz's Bayesian Criterion   =   -48096.369

 ==> multiplied by -2
 Log Likelihood                 =    96143.542
 Akaike's Information Criterion =    96157.542
 Schwarz's Bayesian Criterion   =    96192.739

Variable                 Estimate     AsymStdError        z-value        p-value
----------------     ------------     ------------   ------------   ------------
BETA (regression coefficients)
Intercept                42.57119          0.55126       77.22558        0.00000
DOW                       0.40004          0.04750        8.42273        0.00000
SEX                      -0.73211          0.60471       -1.21067        0.22602
ALPHA (BS variance parameters: log-linear model)
Intercept                 4.28008          0.09600       44.58391        0.00000
SEX                      -0.19562          0.11361       -1.72189        0.08509
TAU (WS variance parameters: log-linear model)
Intercept                 4.88403          0.02752      177.50260        0.00000
SEX                      -0.06836          0.03150       -2.17029        0.02999
```

```
-----------------------
Model WITH RANDOM Scale
-----------------------
 Total  Iterations =  17
 Final Ridge value = 0.0

 Log Likelihood                 =   -47914.216
 Akaike's Information Criterion =   -47923.216
 Schwarz's Bayesian Criterion   =   -47945.843

 ==> multiplied by -2
 Log Likelihood                 =    95828.432
 Akaike's Information Criterion =    95846.432
 Schwarz's Bayesian Criterion   =    95891.686

Variable                 Estimate     AsymStdError        z-value        p-value
----------------     ------------     ------------   ------------   ------------
BETA (regression coefficients)
Intercept                42.57868          0.55031       77.37179        0.00000
DOW                       0.39158          0.04563        8.58138        0.00000
SEX                      -0.73865          0.60904       -1.21281        0.22520
ALPHA (BS variance parameters: log-linear model)
Intercept                 4.28473          0.09386       45.65031        0.00000
SEX                      -0.14654          0.11109       -1.31913        0.18713
TAU (WS variance parameters: log-linear model)
Intercept                 4.78679          0.03843      124.56564        0.00000
SEX                      -0.04782          0.04447       -1.07528        0.28225
Random scale standard deviation
Std Dev                   0.40712          0.02080       19.57251        0.00000
Random location (mean) effect on WS variance
Loc  Eff                 -0.14581          0.02253       -6.47252        0.00000
```

34. The OUT file with suffix_2 contains the results from Stage 2 model.

Mixwild_example_data_Output_2 - Notepad

File Edit Format View Help

```
Mixwild Example2
Created with MixWILD GUI

 Level 2 obervations =        1128
------------
Descriptives
------------

 Dependent variable
                           mean          min          max      std dev
 ----------------------------------------------------------------------
 SED_daily_hrs           9.3941       5.3500      15.3000       1.5909


 Independent variables
                           mean          min          max      std dev
 ----------------------------------------------------------------------
 Age                    -2.1523     -23.5300      47.4700      16.4941


 Random Location and Scale EB mean estimates
                           mean          min          max      std dev
 ----------------------------------------------------------------------
 Locat_1                 0.0000      -2.4318       2.4368       0.9075
 Scale                  -0.0000      -1.7895       2.0324       0.6370
 Locat_1*Scale          -0.0242      -3.3730       3.6866       0.6370
```

35. Location_1 (random intercept) and Scale (random scale) main effects and their interaction (Location_1*Scale) are default regressors in Stage 2 model results.

```
-------------
Final Results
-------------
 Average Log Likelihood         =     -2106.858 (sd= 0.712)
 Akaike's Information Criterion =     -2113.858
 Schwarz's Bayesian Criterion   =     -2131.457

 ==> multiplied by -2
 Log Likelihood                 =      4213.717
 Akaike's Information Criterion =      4227.717
 Schwarz's Bayesian Criterion   =      4262.914


Variable               Estimate     AsymStdError          z-value          p-value
----------------     ------------   ------------     ------------     ------------
Intercept               9.37372        0.06947        134.92350          0.00000
Age                     0.01798        0.00439          4.09480          0.00004
Locat_1                 0.07699        0.06590          1.16843          0.24263
Locat_1*Age            -0.00310        0.00360         -0.85909          0.39029
Scale                   0.03241        0.08328          0.38913          0.69718
Scale*Age              -0.00030        0.00455         -0.06596          0.94741
Locat_1*Scale          -0.00638        0.08802         -0.07246          0.94224
Resid.Variance          2.45392        0.10338         23.73801          0.00000
```

4.2. Brief interpretation of the Mixregls - Linear Regression model results

Stage 1 model with random scale parameters

```
-----------------------
Model WITH RANDOM Scale
-----------------------

Variable                 Estimate    AsymStdError         z-value         p-value
----------------     ------------    ------------    ------------    ------------
BETA (regression coefficients)
Intercept                42.56827         0.55150        77.18565         0.00000
DOW                       0.39107         0.04564         8.56896         0.00000
SEX                      -0.75529         0.61028        -1.23761         0.21586
ALPHA (BS variance parameters: log-linear model)
Intercept                 4.28374         0.09391        45.61507         0.00000
SEX                      -0.14547         0.11116        -1.30873         0.19063
TAU (WS variance parameters: log-linear model)
Intercept                 4.78671         0.03845       124.50564         0.00000
SEX                      -0.04727         0.04449        -1.06250         0.28801
Random scale standard deviation
Std Dev                   0.40728         0.02081        19.57622         0.00000
Random location (mean) effect on WS variance
Loc  Eff                 -0.14551         0.02253        -6.45824         0.00000
```

- The Stage 1 model (with random scale) shows that "Day of week (DOW)" is positively related to positive affect (mean estimate=0.3911, $p<.01$). People report higher positive affect when it is closer to weekend (0=Monday, 1=Tuesday,…, 6=Sunday. (As mentioned earlier, DOW is used as a linear regressor of positive affect for this example. Users might want to instead treat this as a factor in the regression model by creating 6 dummy codes for the 7 days).
- The Stage 1 model also shows that there is significant variability in scale across subjects, as the Std Dev for the Random scale (on the log scale) is estimated as 0.4073 ($p<.001$). Therefore, individuals differ from each other in their degree of within-subject/intraindividual variability in positive affect.
- The Stage 1 model also shows that the random scale (i.e., within-subject/intraindividual variance) is negatively associated with the random intercept (i.e., within-subject mean), as indicated by the estimate for the Loc Eff for Random location (mean) effect on WS variance (estimate = -0.14551, $p<.001$). Individuals with overall higher mean positive affect are *less erratic/more stable* in their positive affect responses. (Note that this result may be also due to a ceiling effect in the affect response scale).

Stage 2 model with continuous subject-level outcome

```
-------------
Final Results
-------------


Variable               Estimate    AsymStdError        z-value        p-value
----------------    ------------   ------------   ------------   ------------
Intercept               9.37388        0.06878      136.29122        0.00000
Age                     0.01783        0.00467        3.81784        0.00013
Locat_1                 0.08217        0.06927        1.18612        0.23557
Locat_1*Age            -0.00307        0.00364       -0.84282        0.39933
Scale                   0.04340        0.08158        0.53200        0.59473
Scale*Age              -0.00018        0.00502       -0.03537        0.97178
Locat_1*Scale          -0.01308        0.09619       -0.13600        0.89182
Resid.Variance          2.47808        0.10483       23.63836        0.00000
```

- In the Stage 2 final results table, *Locat_1* refers to the effect of the random intercept (i.e., within-subject mean) on average hours per day of sedentary behavior; *Scale* refers to the effect of random scale (i.e., within-subject variance) on average hours per day of sedentary behavior; and *Locat_1* Scale* is the interaction between random intercept and random scale predicting average hours per day of sedentary behavior.
- After controlling for other variables in the model, this linear regression model shows that "Age" is positively related to average hours per day of sedentary behavior (estimate=0.0178, p<.001), indicating that older subjects spend more time being sedentary.
- The interaction between Age and the Random Intercept and the interaction between Age and Random Scale do not predict individuals' average hours per day of sedentary behavior.
- Also, the random intercept (i.e., within-subject mean), random scale (i.e., within-subject variance), and their interaction do not predict individuals' average hour per day of sedentary behavior.

Note:

1. In these output files, the numbers of level 1 and level 2 observations correspond to the available non-missing observations of level 1 and level 2 variables included in the model(s).
2. For a given run, the program includes observations that have non-missing data for the outcomes (both Stage 1 and Stage 2), and all regressors (both Stage 1 and Stage 2) included in that particular run. Thus, the program uses all available data for a given run.
3. Refer to the supplemental documents for more detailed explanation of the Stage 1 and Stage 2 model results.

4. Example 3: Running Mixregls- Logistic Regression model in MixWILD

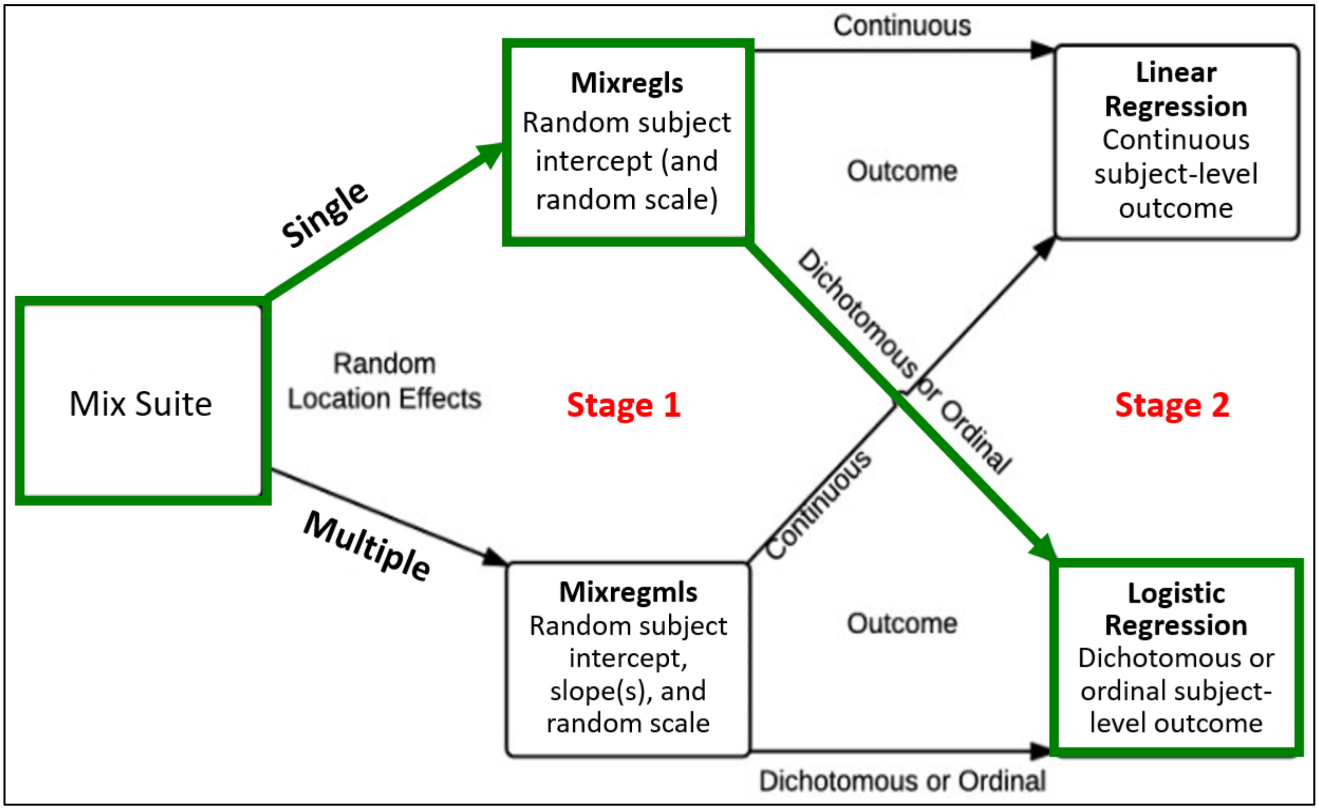

Example research question for Mixregls-Logistic Regression model

- Examine whether subject-level mean (i.e., random intercept or within-person mean) and subject level variance (i.e., random scale or degree of within-subject/intraindividual variability) of momentary positive affect (within-subject, continuous, time-varying variable) predict subject-level obesity risk (between-subject, dichotomous, time invariant variable).

Stage 1 outcome variable: Positive affect (time varying)
Stage 1 regressors: None (an empty model)
Stage 2 outcome variable: Obese vs Non-obese (time invariant)
Stage 2 regressors: Random intercept; Random scale; Random intercept x Random scale interaction (these are all default regressors in Stage 2 model results)

4.1. Step-by-step instructions on running Mixregls-Logistic regression model in MixWILD

1. Double-click on the MixWILD icon to open the main window.
2. Click on **“File”** and then select **“New Model”** (or use keyboard shortcut Ctrl + N).

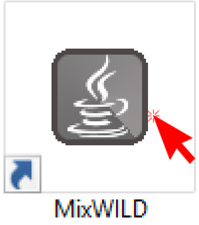

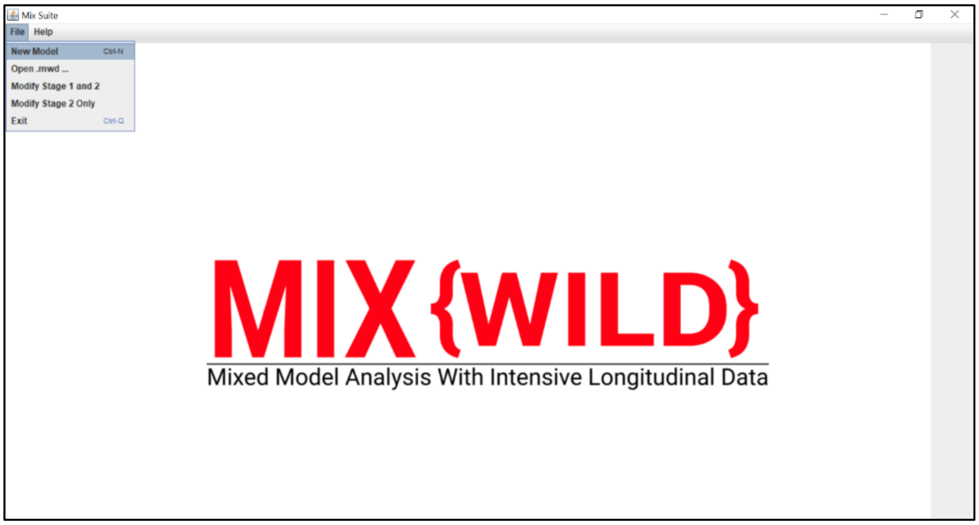

3. Click on **“Instructions”** to make sure your data are in the correct format.

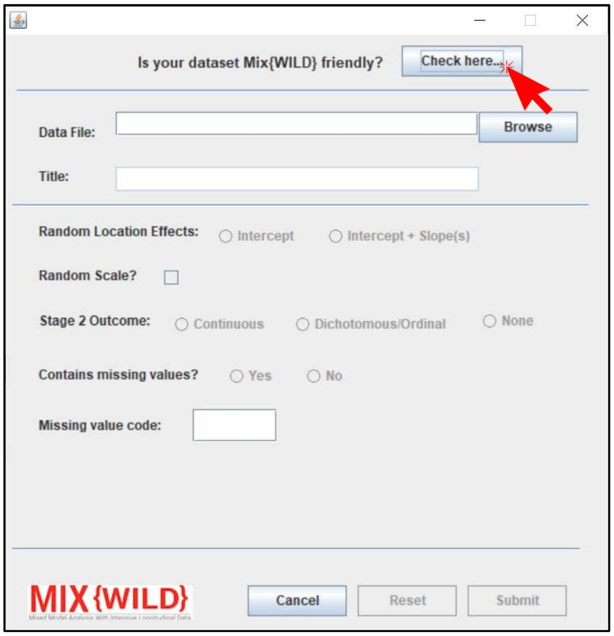

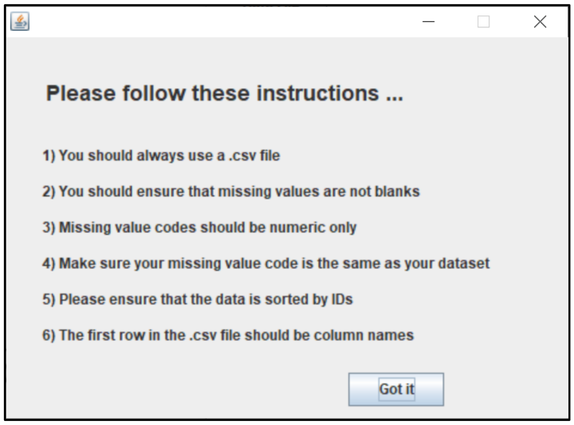

4. Click on **"Browse"** to select the location of your data file (in .csv) and then click **"Open"**.

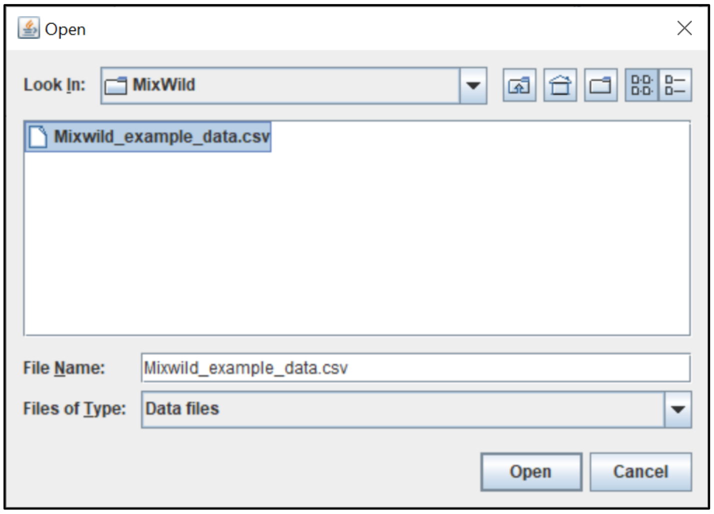

5. Click on **"View Data"** to preview your data file to verify your data and format are correct.

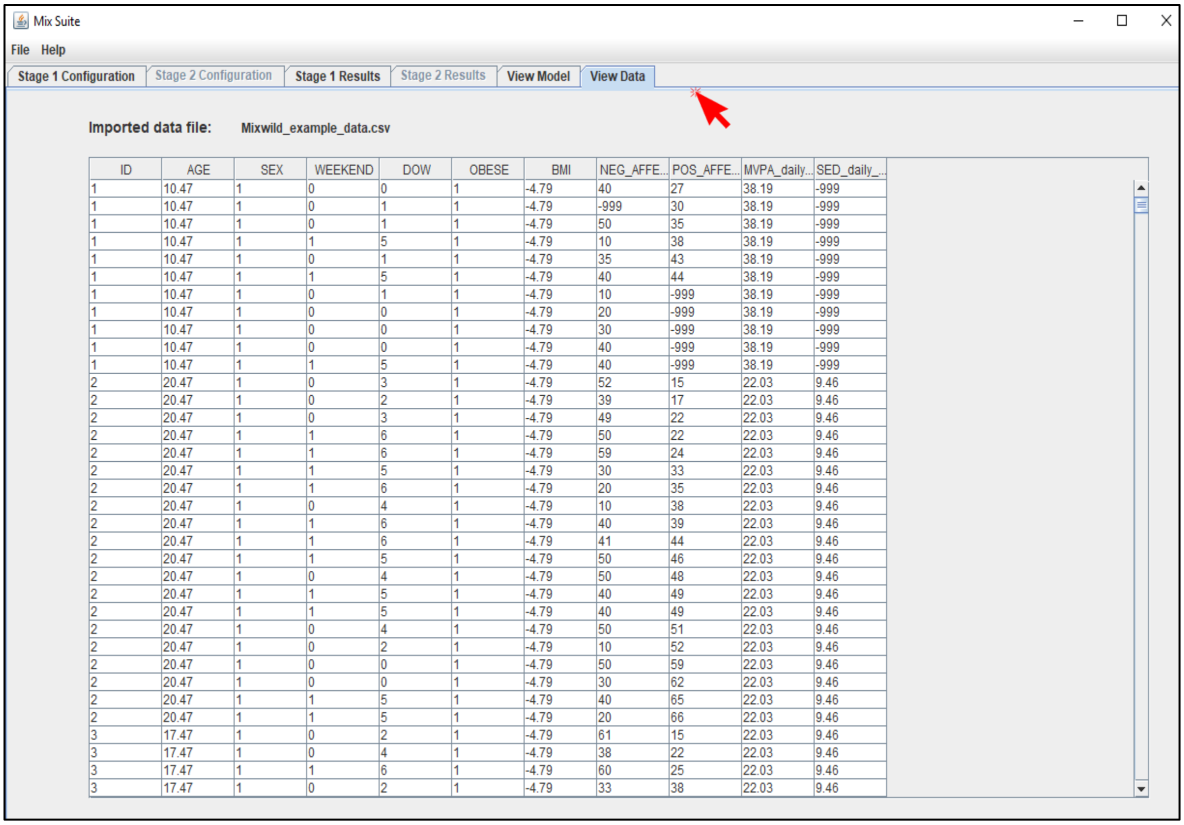

6. Add title to your output files.
7. Select **"Intercept"** from Random Location Effects specification for subject-level mean. Select **"Random Scale"** for subject-level variability.
8. Select **"Dichotomous/Ordinal"** for Stage 2 outcome.
9. Click on missing values if there are any in your dataset; specify the missing value code in the box (i.e., -999 in the example dataset).

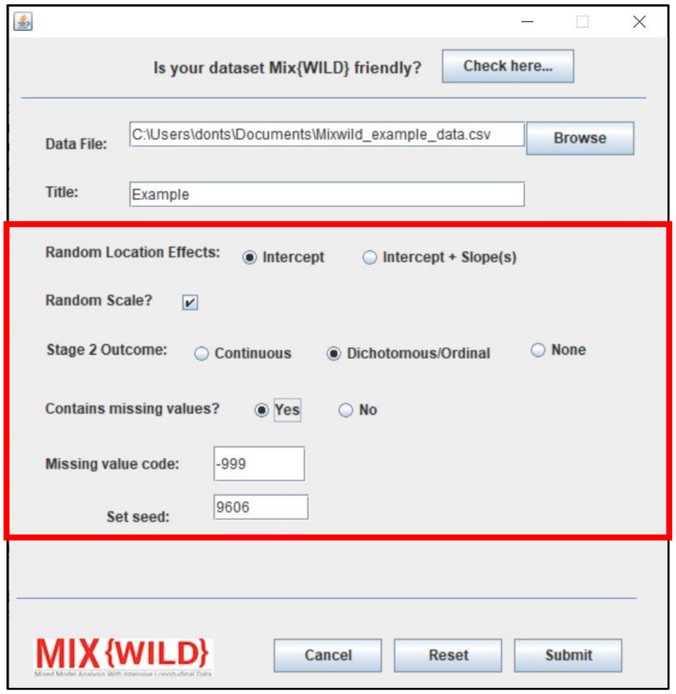

10. After you submit, the interface will take you to the page that enables you to configure your Stage 1 model.

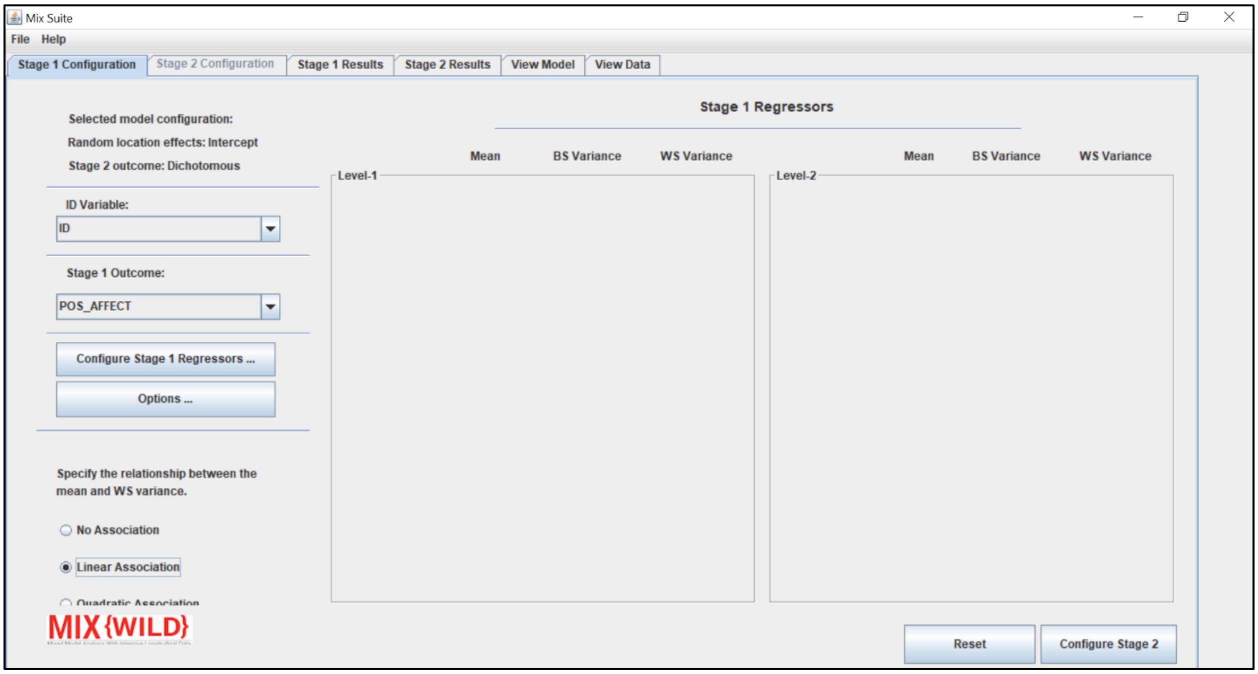

11. On the Stage 1 configuration page, select your ID variable and positive affect (a time-varying variable) as your Stage 1 outcome variable.
12. Specify the association between the mean and within- subject (WS) variance, which is the association of the random location and random scale effects. **"Linear Association"** is selected for this example. For random scale models, a quadratic association is also possible.

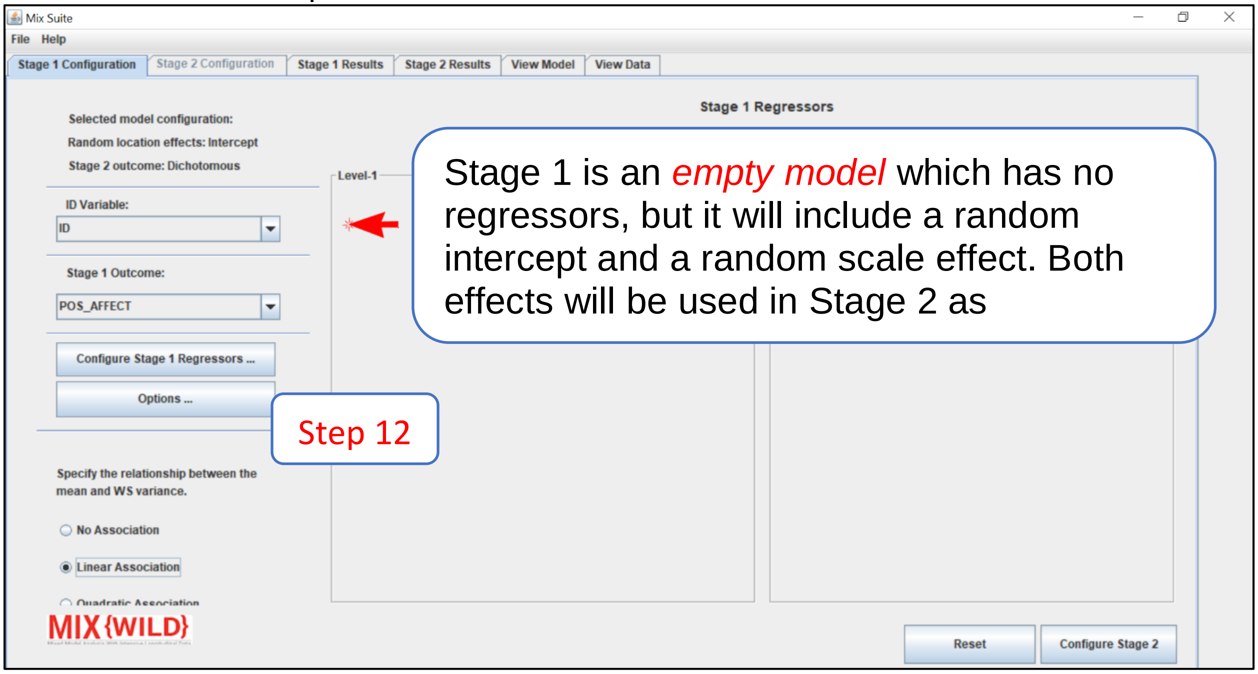

13. Click on **"Options"** to change other default settings if needed.
14. By default, the estimated random effects of the Stage 1 analysis (i.e., random location and scale effects) are resampled 500 times in the Stage 2 analysis. Resampling is necessary because the random effects are estimated quantities that are entered as regressors in the Stage 2 model.
15. Leave **"Discard Subjects"** unchecked, so we do not drop participants who have 0 variation in their Stage 1 outcome variable. Click on **"Submit"**

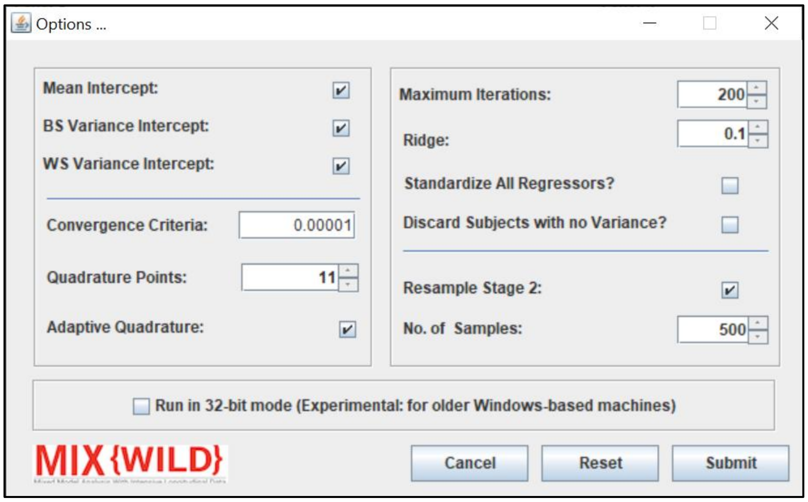

(Refer to the supplemental documents for further description of Options settings)

16. In **“Configure Stage 2”**, select Stage 2 outcome variable. It should be a dichotomous or ordinal time-invariant variable from your data set.

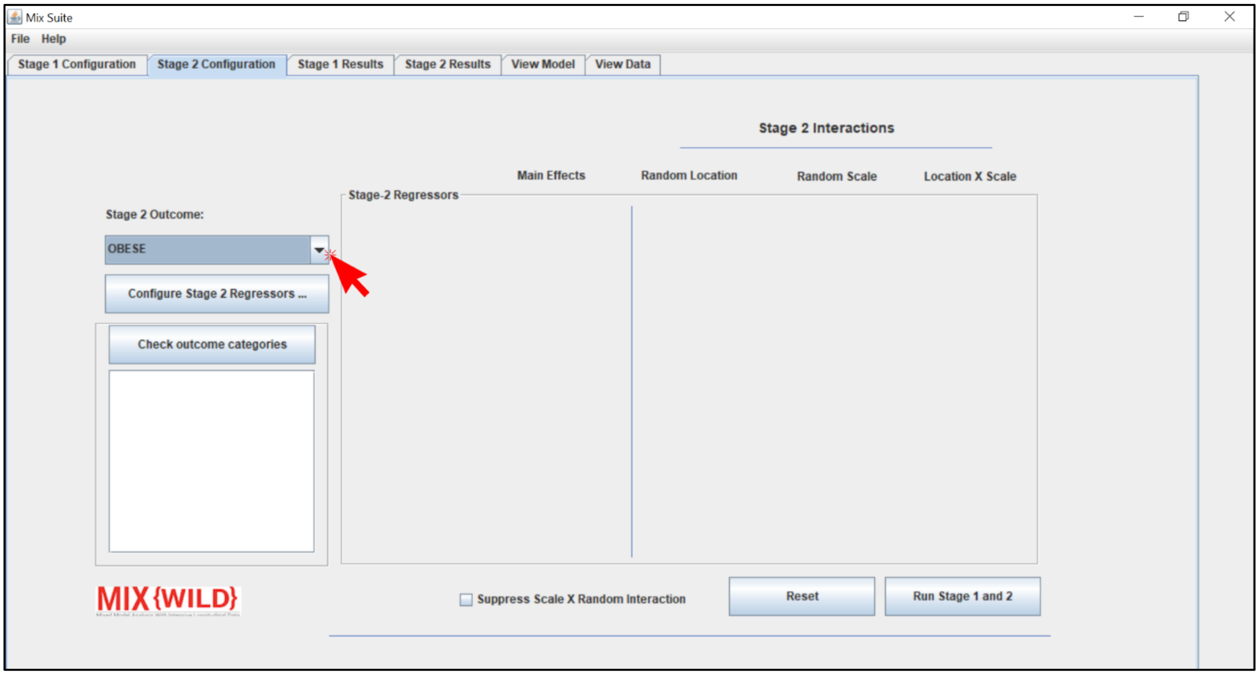

17. Select **“Obese”** as the Stage 2 outcome variable and check if the outcome categories are correct.

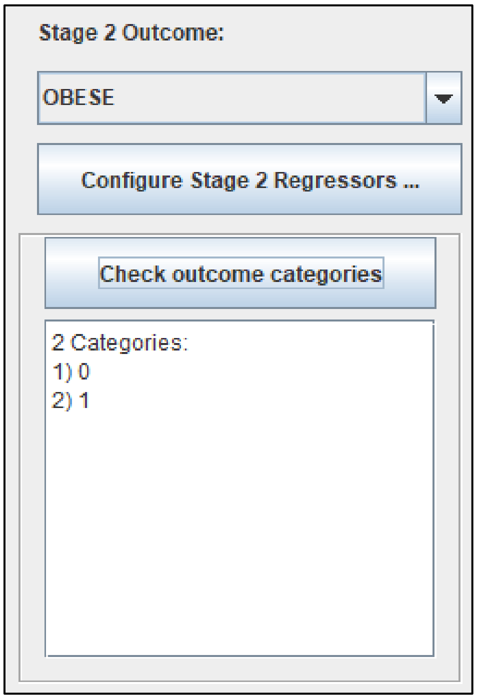

18. Stage 2 also has no regressors, so you don’t need to click on the “Configure Stage 2 Regressors” button. The random intercept, random scale, and the random intercept x random scale interaction will be included as default regressors in Stage 2.

19. Click on Stage 1 and Stage 2 configurations to double-check your model. Click on **“Run Stage 1 and 2”** to generate the definition file.

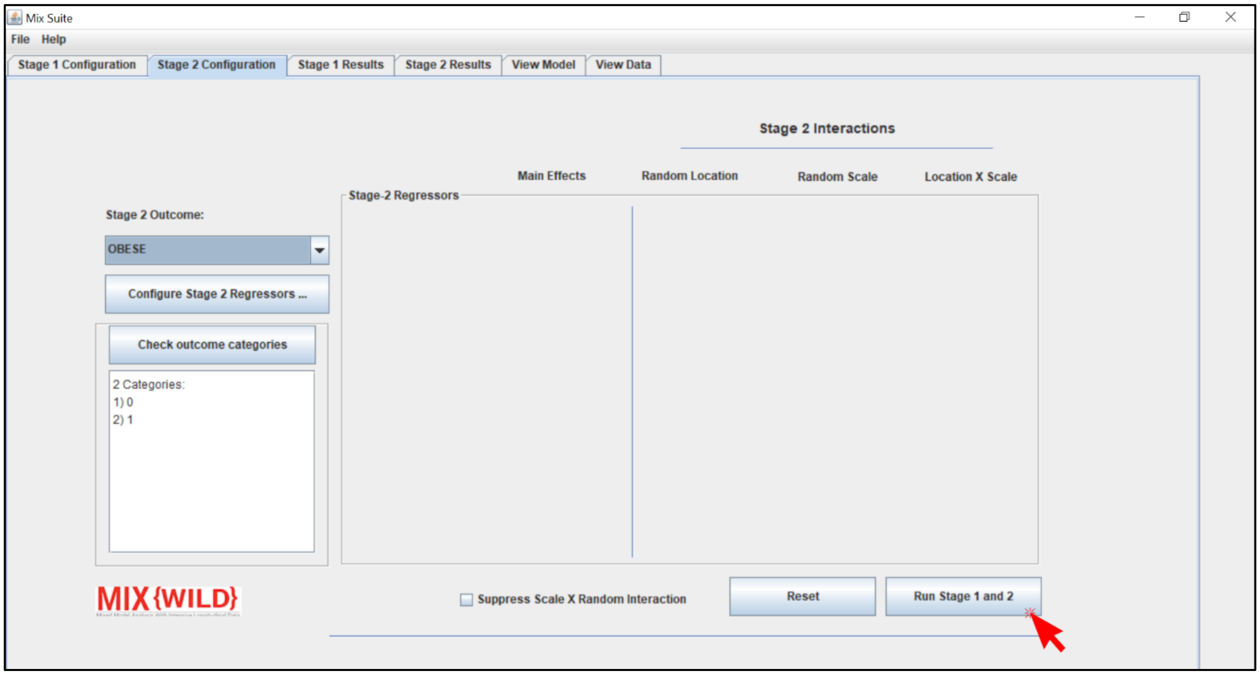

20. The definition file contains the syntax that instructs the program to estimate the specified model. In the definition file, click on **“Proceed”** to run your model and generate model output files.

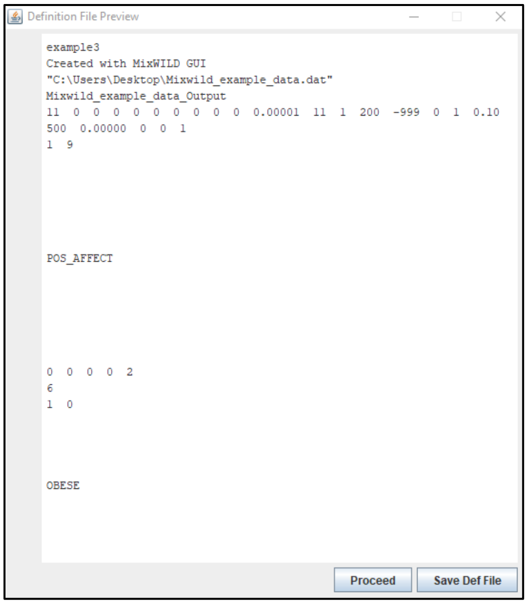

21. A window will appear while model estimation is in progress.

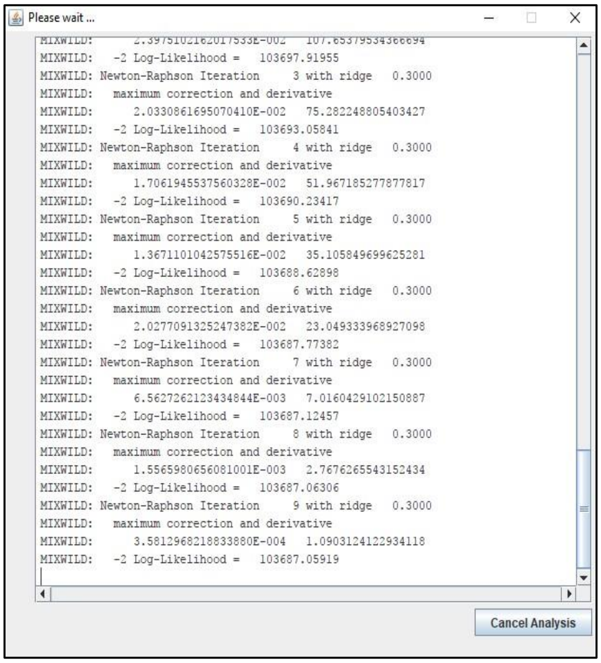

22. If the following warning message appears, it indicates that computational difficulties were encountered and prevented the model parameters from being estimated successfully. In this case, double-check the format of your dataset and your model specifications. Some suggestions for steps to take are listed in Appendix A.

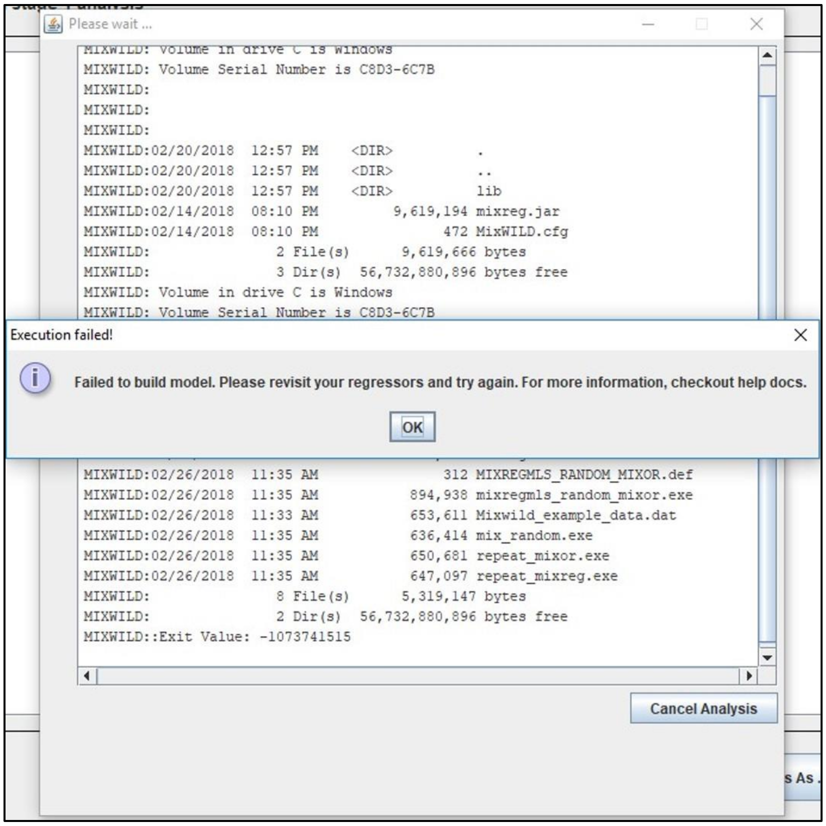

23. When estimation is completed, the Stage 1 and Stage 2 results can be seen by clicking the Stage 1 and Stage 2 Results boxes, respectively.

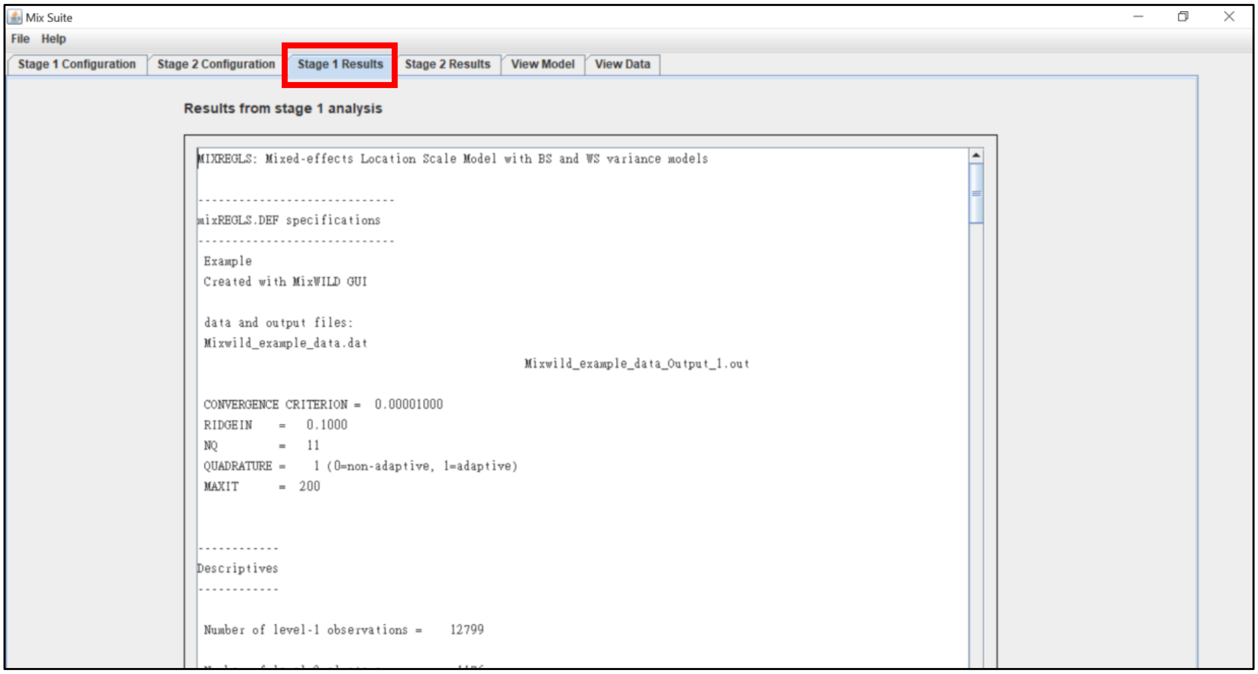

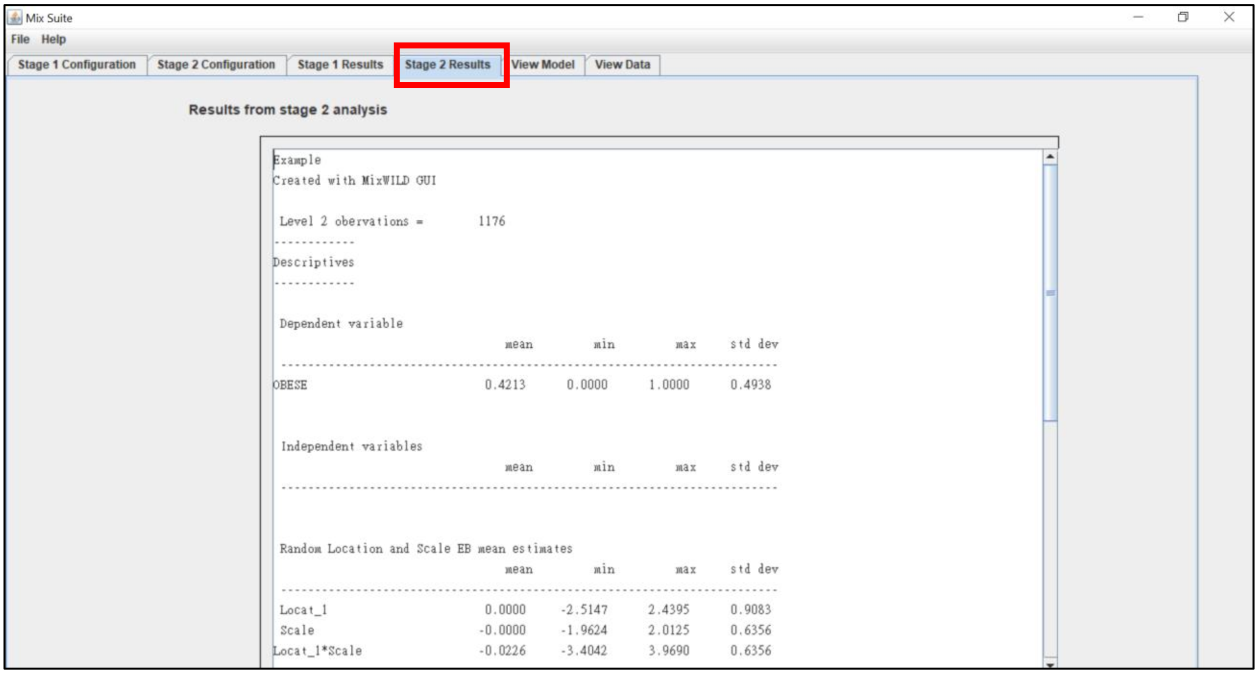

24. All files generated from the program can be found in a folder with the prefix MixWILD under the same directory of your dataset.

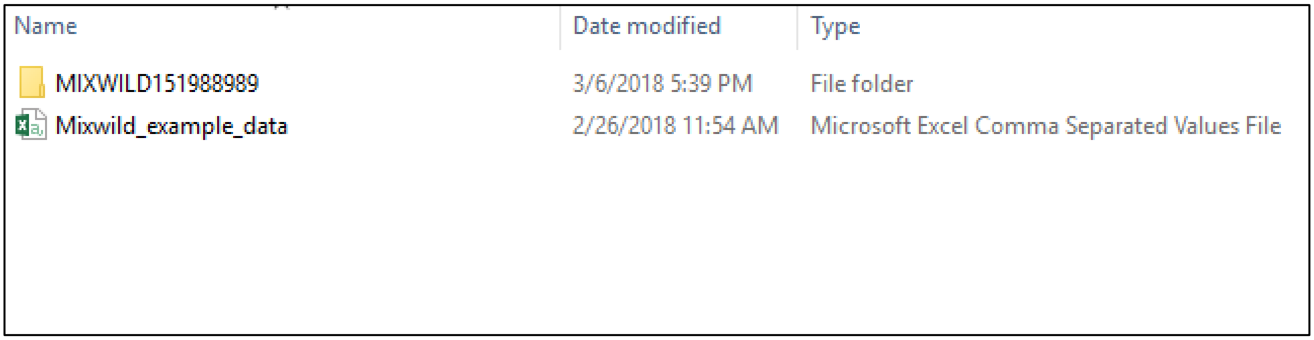

Name	Date modified	Type
MIXWILD151988989	3/6/2018 5:39 PM	File folder
Mixwild_example_data	2/26/2018 11:54 AM	Microsoft Excel Comma Separated Values File

25. The output files with suffix_1 and _2 are the results that are identical in the Stage 1 and Stage 2 boxes in the MixWILD program.

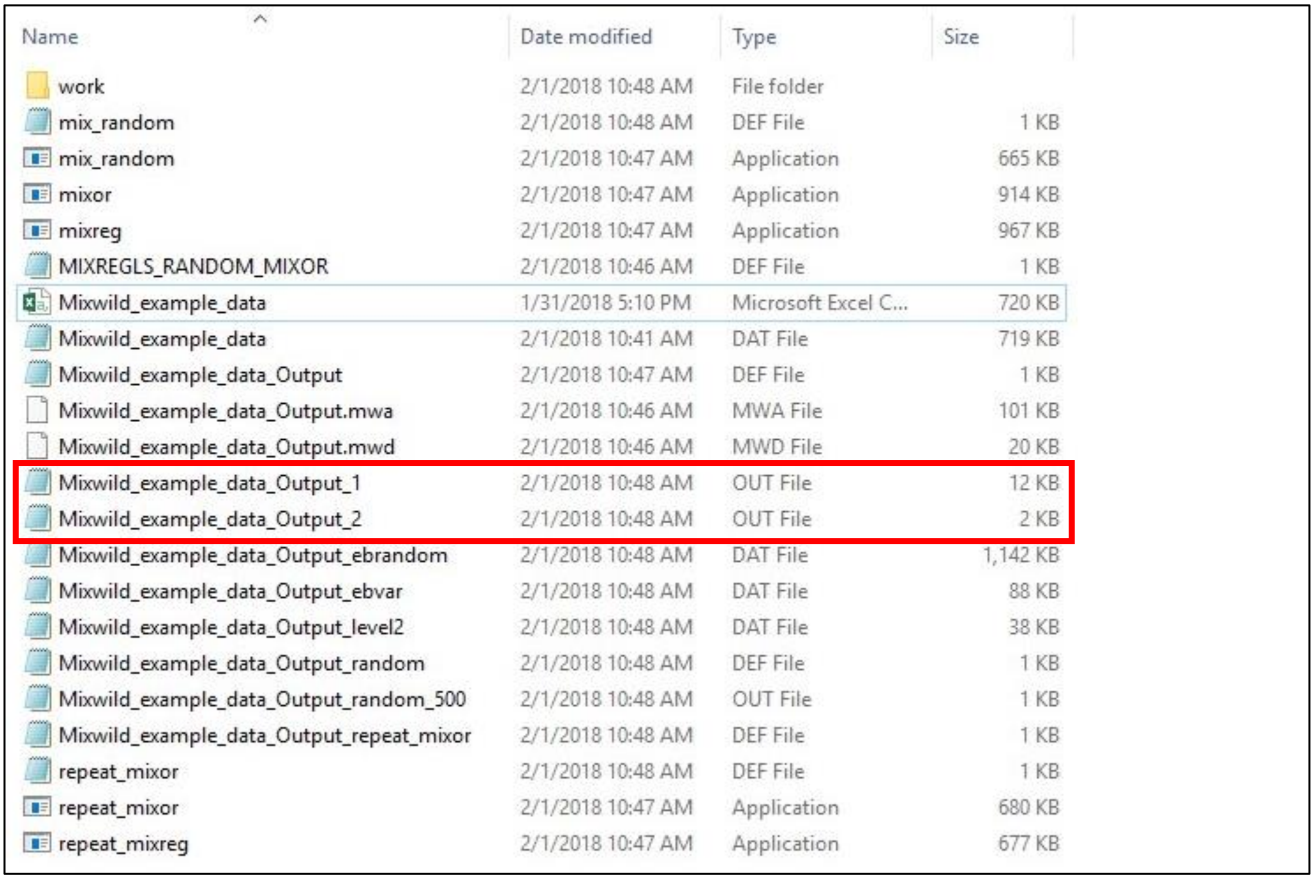

Name	Date modified	Type	Size
work	2/1/2018 10:48 AM	File folder	
mix_random	2/1/2018 10:48 AM	DEF File	1 KB
mix_random	2/1/2018 10:47 AM	Application	665 KB
mixor	2/1/2018 10:47 AM	Application	914 KB
mixreg	2/1/2018 10:47 AM	Application	967 KB
MIXREGLS_RANDOM_MIXOR	2/1/2018 10:46 AM	DEF File	1 KB
Mixwild_example_data	1/31/2018 5:10 PM	Microsoft Excel C...	720 KB
Mixwild_example_data	2/1/2018 10:41 AM	DAT File	719 KB
Mixwild_example_data_Output	2/1/2018 10:47 AM	DEF File	1 KB
Mixwild_example_data_Output.mwa	2/1/2018 10:46 AM	MWA File	101 KB
Mixwild_example_data_Output.mwd	2/1/2018 10:46 AM	MWD File	20 KB
Mixwild_example_data_Output_1	2/1/2018 10:48 AM	OUT File	12 KB
Mixwild_example_data_Output_2	2/1/2018 10:48 AM	OUT File	2 KB
Mixwild_example_data_Output_ebrandom	2/1/2018 10:48 AM	DAT File	1,142 KB
Mixwild_example_data_Output_ebvar	2/1/2018 10:48 AM	DAT File	88 KB
Mixwild_example_data_Output_level2	2/1/2018 10:48 AM	DAT File	38 KB
Mixwild_example_data_Output_random	2/1/2018 10:48 AM	DEF File	1 KB
Mixwild_example_data_Output_random_500	2/1/2018 10:48 AM	OUT File	1 KB
Mixwild_example_data_Output_repeat_mixor	2/1/2018 10:48 AM	DEF File	1 KB
repeat_mixor	2/1/2018 10:48 AM	DEF File	1 KB
repeat_mixor	2/1/2018 10:47 AM	Application	680 KB
repeat_mixreg	2/1/2018 10:47 AM	Application	677 KB

26. There are two submodel results in Output_1. The first submodel does not include scale parameters; the second submodel includes the random scale estimates.
27. A brief description of the results from the second submodel will be provided in the following section.

4.2. Brief interpretation of Mixregls-Logistic regression model results

Stage 1: model with random scale parameters

```
-----------------------
Model WITH RANDOM Scale
-----------------------
 Total  Iterations =  15
 Final Ridge value = 0.0

 Log Likelihood                 =   -49992.010
 Akaike's Information Criterion =   -49997.010
 Schwarz's Bayesian Criterion   =   -50009.685

 ==> multiplied by -2
 Log Likelihood                 =    99984.021
 Akaike's Information Criterion =    99994.021
 Schwarz's Bayesian Criterion   =   100019.370


Variable                 Estimate    AsymStdError        z-value        p-value
----------------     ------------    ------------   ------------   ------------
BETA (regression coefficients)
Intercept                43.31917         0.26098      165.98699        0.00000
ALPHA (BS variance parameters: log-linear model)
Intercept                 4.18557         0.04906       85.31084        0.00000
TAU (WS variance parameters: log-linear model)
Intercept                 4.75294         0.01914      248.26182        0.00000
Random scale standard deviation
Std Dev                   0.40459         0.02046       19.77955        0.00000
Random location (mean) effect on WS variance
Loc  Eff                 -0.13583         0.02190       -6.20293        0.00000
```

- Since there are no regressors in the Stage 1 model, only the intercept estimates are presented in the mean (Beta), between-subject (Alpha), and the within-subject (Tau) sections in the Stage 1 results.
- The Stage 1 model shows that there is significant variability in scale across subjects, as the Std Dev for the Random scale (on the log scale) is estimated as 0.4046 (p<.001). Therefore, individuals differ from each other in their degree of within-subject/intraindividual variability in positive affect.
- The Stage 1 model also shows that the random scale (i.e., within-subject/intraindividual variance) is negatively associated with the random intercept (i.e., within-subject mean), as indicated by the estimate for the Loc Eff for Random location (mean) effect on WS variance (estimate = -0.1358, p<.001). Individuals with overall higher mean positive affect are less erratic/more stable in their positive affect responses. (Note that this result may be also due to a ceiling effect in the affect response scale).

Stage 2: model with a dichotomous subject-level outcome

Mixwild_example_data_Output_2 - Notepad

File Edit Format View Help

```
Mixwild example3
Created with MixWILD GUI

 Level 2 obervations =        1176

-------------
Final Results
-------------


Variable               Estimate     AsymStdError         z-value         p-value
----------------    ------------    ------------    ------------    ------------
Intercept               0.11711         0.08991         1.30248         0.19275
Locat_1                 0.41851         0.08533         4.90443         0.00000
Scale                   0.12689         0.10840         1.17059         0.24176
Locat_1*Scale          -0.09245         0.11596        -0.79726         0.42530
```

- In the Stage 2 final results table, *Locat_1* refers to the effect of the random intercept (i.e., within-subject mean) on obesity risk; *Scale* refers to the effect of random scale (i.e., within-subject variance) on obesity risk; and *Locat_1* Scale* is the interaction between random intercept and random scale of positive affect in predicting obesity risk (a subject-level binary variable).
- The Stage 2 model does not include regressors, however, the random intercept (*Locat_1*), the random scale (*Scale*), and the interaction effect (*Locat_1*Scale*) are default regressors in the Stage 2 final results table.
- This logistic regression model shows that the random intercept (*Locat_1*) is positively associated with obesity risk (estimate OR = -0.4185, p<.001). Subjects who have higher means levels of positive affect than others are less likely to be obese.
- The interaction between the random intercept and random slope of positive affect does not predict individual's obesity risk.

Note:

1. The numbers of level 1 and level 2 observations correspond to the available non-missing observations of level 1 and level 2 variables included in both Stage 1 and Stage 2 models. Thus, since there are no regressors, observations with missing positive affect (level-1) or missing obesity (level-2) are not included in either Stage 1 or Stage 2 analyses.
2. Please refer to the supplemental documents for more detailed explanation of the Stage 1 and Stage 2 model results.

5. Example 4: Running Mixregmls- Linear regression model in MixWILD

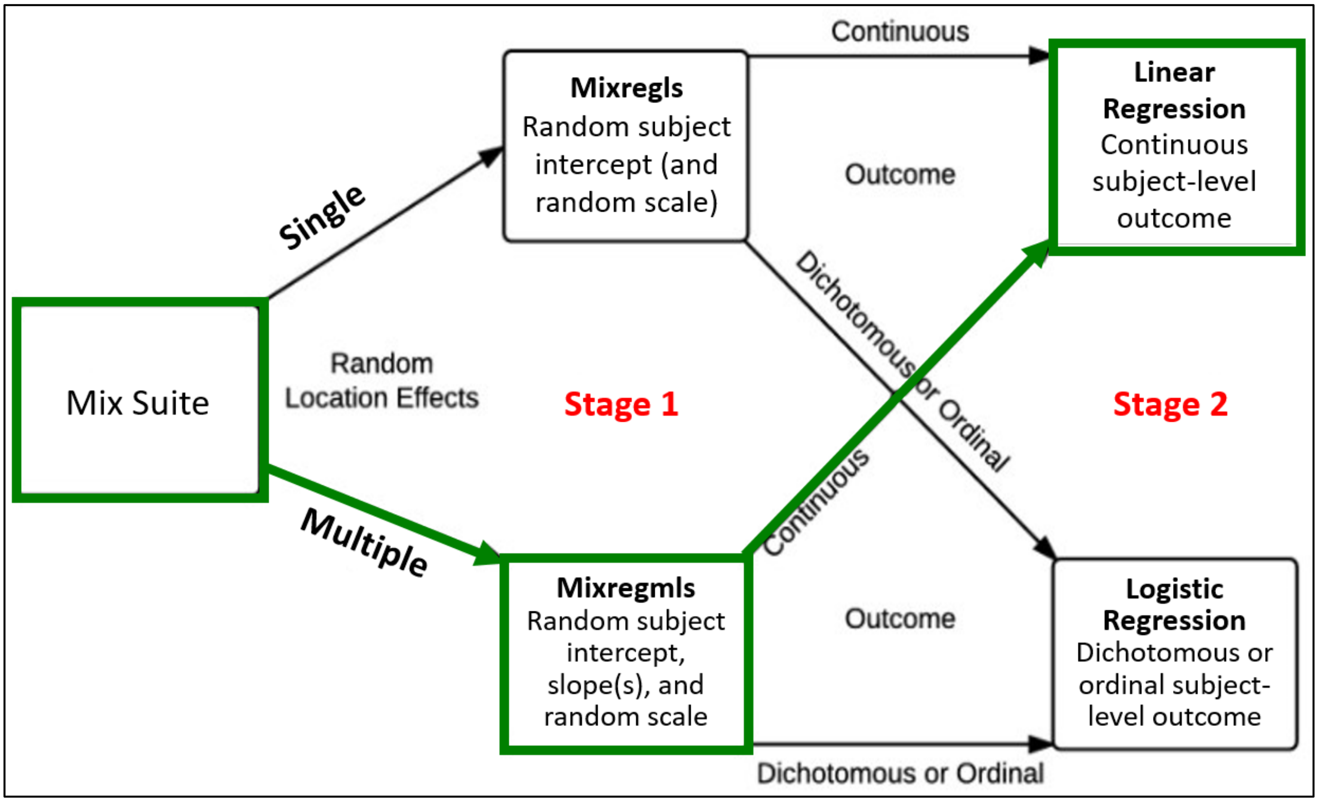

Example research question for Mixregmls-Linear Regression model

- Examine whether subject-level mean (i.e., random intercept or within-person mean) and subject-level variance (i.e., random scale or degree of within-person / intraindividual variability) of momentary positive affect (within-subject, continuous, time-varying variable) predicts subject-level average minutes per day of moderate to vigorous physical activity (MVPA; between-subject, continuous, time-invariant variable), in addition to their positive affect change between weekdays and weekends (within-subject, dichotomous, time-varying variable), and BMI (between-subject, continuous, time-invariant variable).
- Examine whether there is significant variability between individuals in the ***association*** (i.e., random slope) between weekday/weekend and momentary positive affect (i.e., whether individuals differ from each other in the extent to which positive affect increases towards the end of the week), after controlling for subject-level mean and subject-level variance.
- Examining whether the variability between individuals in the ***association*** (i.e., random slope) between weekday/weekend and momentary positive affect predicts average minutes per day of MVPA, after controlling for BMI.

Stage 1 outcome variable: Positive affect (time-varying)
Stage 1 regressor (with random slope): Weekday/Weekend (time-varying)
Stage 2 outcome variable: Average minutes per day of MVPA (time-invariant)
Stage 2 regressor: BMI (time-invariant)

5.1. Step-by-step instructions on running Mixregmls - Linear regression model in MixWILD

1. Double-click on the the MixWILD icon to open the main window.
2. Click on **“File”** and then select **“New Model”** (or use keyboard shortcut Ctrl + N).

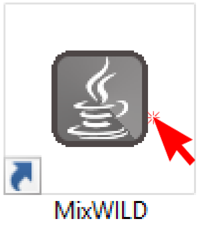

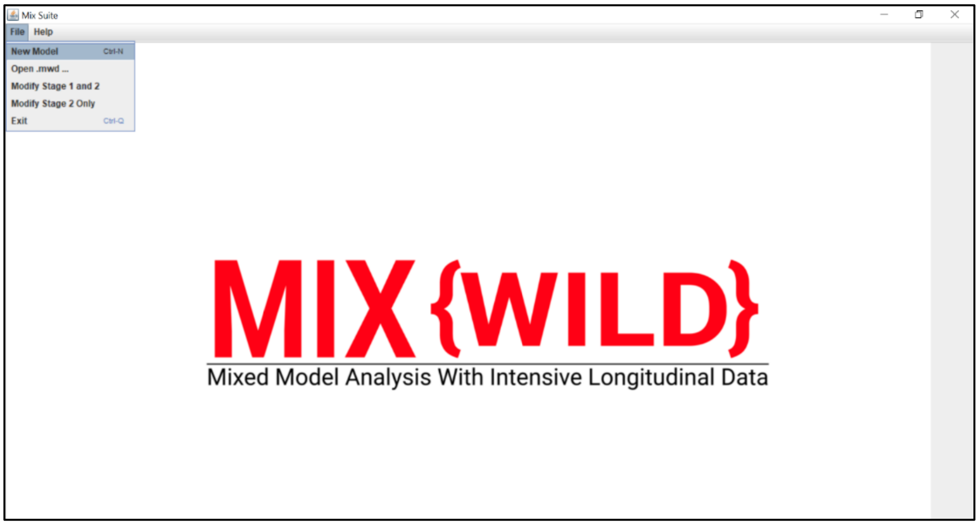

3. Click on **“Instructions”** to make sure your data are in the correct format.

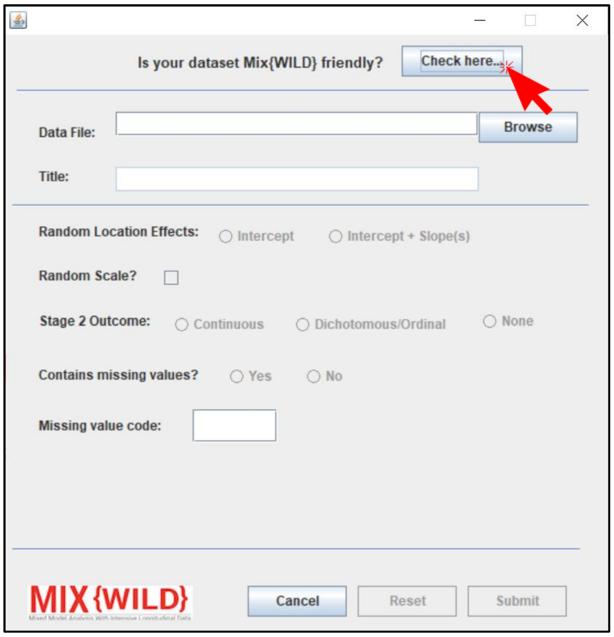

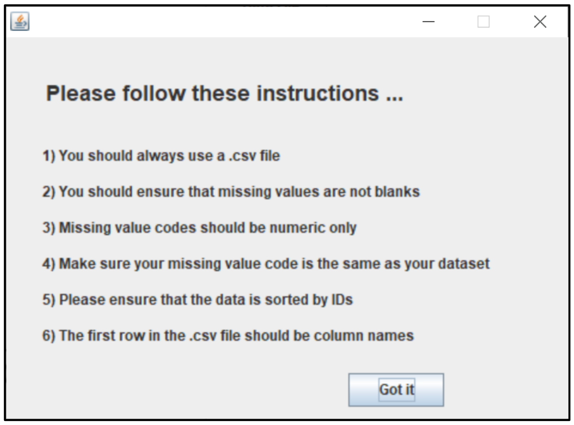

4. Click on **"Browse"** to select the location of your data file (in .csv) and then click **"Open"**.

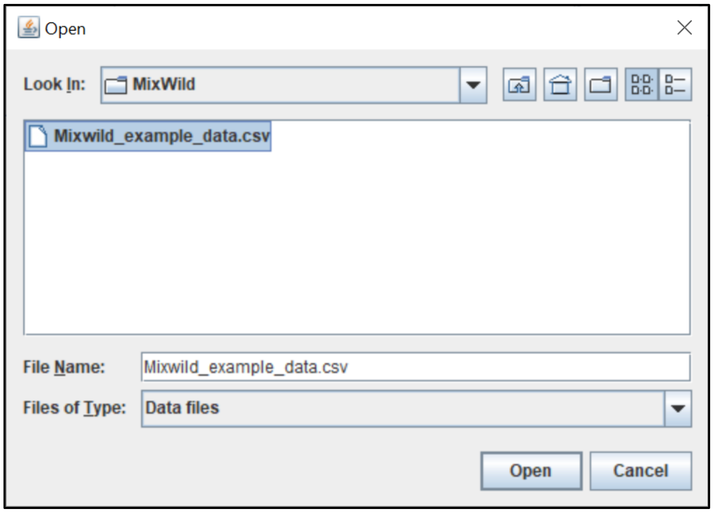

5. Click on **"View Data"** to preview your data file to verify your data and format are correct.

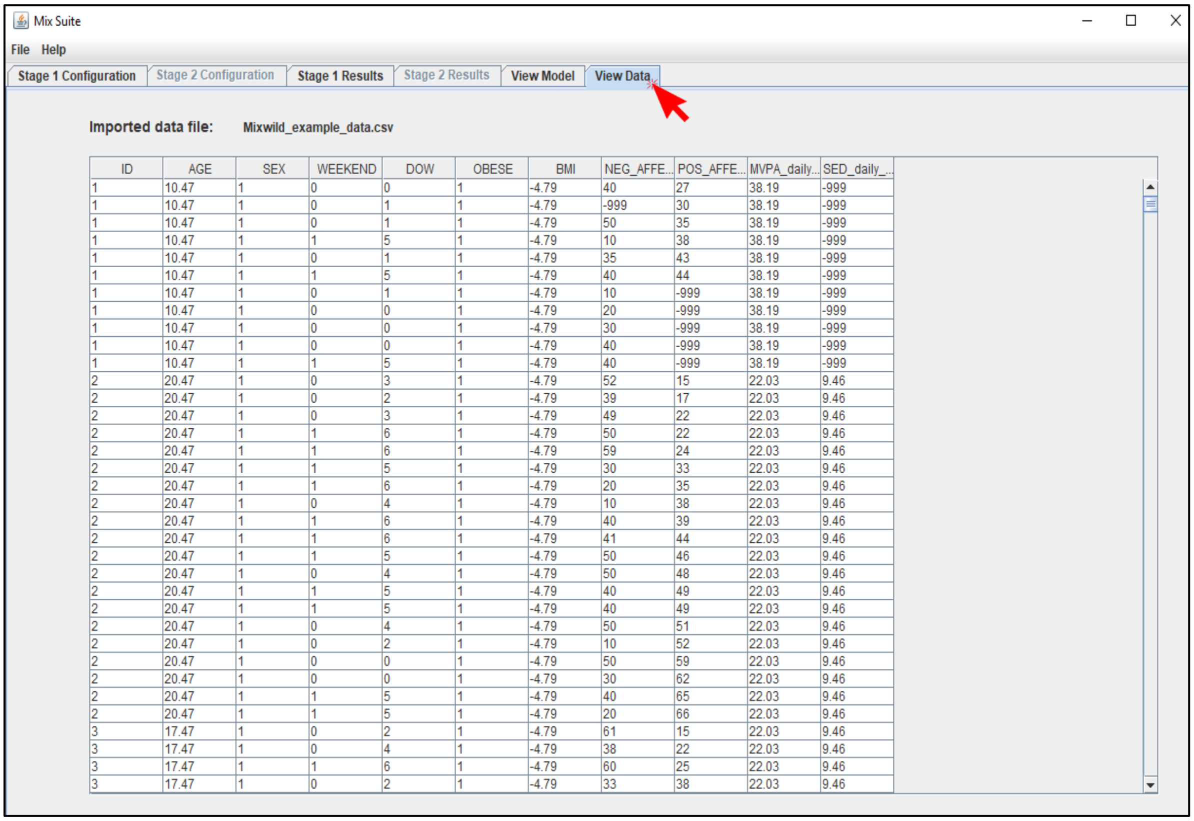

ID	AGE	SEX	WEEKEND	DOW	OBESE	BMI	NEG_AFFE...	POS_AFFE...	MVPA_daily...	SED_daily_...
1	10.47	1	0	0	1	-4.79	40	27	38.19	-999
1	10.47	1	0	1	1	-4.79	-999	30	38.19	-999
1	10.47	1	0	1	1	-4.79	50	35	38.19	-999
1	10.47	1	1	5	1	-4.79	10	38	38.19	-999
1	10.47	1	0	1	1	-4.79	35	43	38.19	-999
1	10.47	1	1	5	1	-4.79	40	44	38.19	-999
1	10.47	1	0	1	1	-4.79	10	-999	38.19	-999
1	10.47	1	0	0	1	-4.79	20	-999	38.19	-999
1	10.47	1	0	0	1	-4.79	30	-999	38.19	-999
1	10.47	1	0	0	1	-4.79	40	-999	38.19	-999
1	10.47	1	1	5	1	-4.79	40	-999	38.19	-999
2	20.47	1	0	3	1	-4.79	52	15	22.03	9.46
2	20.47	1	0	2	1	-4.79	39	17	22.03	9.46
2	20.47	1	0	3	1	-4.79	49	22	22.03	9.46
2	20.47	1	1	6	1	-4.79	50	22	22.03	9.46
2	20.47	1	1	6	1	-4.79	59	24	22.03	9.46
2	20.47	1	1	5	1	-4.79	30	33	22.03	9.46
2	20.47	1	1	6	1	-4.79	20	35	22.03	9.46
2	20.47	1	0	4	1	-4.79	10	38	22.03	9.46
2	20.47	1	1	6	1	-4.79	40	39	22.03	9.46
2	20.47	1	1	6	1	-4.79	41	44	22.03	9.46
2	20.47	1	1	5	1	-4.79	50	46	22.03	9.46
2	20.47	1	0	4	1	-4.79	50	48	22.03	9.46
2	20.47	1	1	5	1	-4.79	40	49	22.03	9.46
2	20.47	1	1	5	1	-4.79	40	49	22.03	9.46
2	20.47	1	0	4	1	-4.79	50	51	22.03	9.46
2	20.47	1	0	2	1	-4.79	10	52	22.03	9.46
2	20.47	1	0	0	1	-4.79	50	59	22.03	9.46
2	20.47	1	0	0	1	-4.79	30	62	22.03	9.46
2	20.47	1	1	5	1	-4.79	40	65	22.03	9.46
2	20.47	1	1	5	1	-4.79	20	66	22.03	9.46
3	17.47	1	0	2	1	-4.79	61	15	22.03	9.46
3	17.47	1	0	4	1	-4.79	38	22	22.03	9.46
3	17.47	1	1	6	1	-4.79	60	25	22.03	9.46
3	17.47	1	0	2	1	-4.79	33	38	22.03	9.46

6. Add title to your output files.
7. Select **“Intercept + Slope”** from Random Location Effects specification for subject-level mean and subject-level slope. Check **“Random Scale”** for subject-level variability.
8. Select **“Continuous”** for Stage 2 outcome.
9. Click on missing values if there are any in your dataset; specify your missing value code in the box (i.e., -999 in the example dataset).

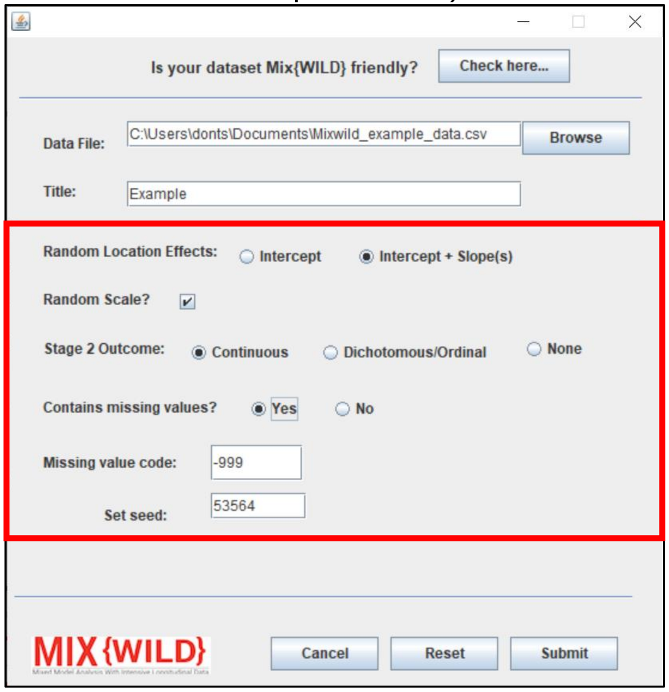

10. After you submit, the interface will take you to the page that enables you to configure your Stage 1 model.

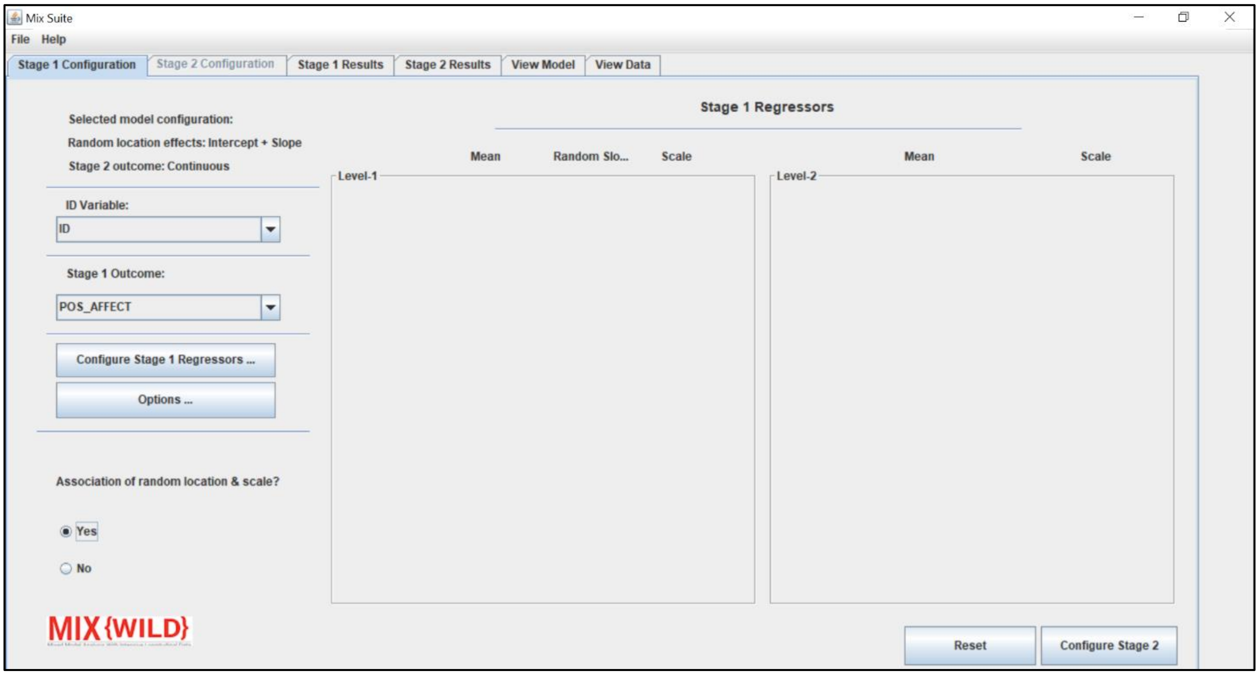

11. On the Stage 1 configuration page, select your ID variable and positive affect as your Stage 1 outcome variable.
12. Specify the association between mean and within- subject (WS) variance, which is the association between the random location and random scale effects. The default is no association, but **"Yes"** is selected for the following example.

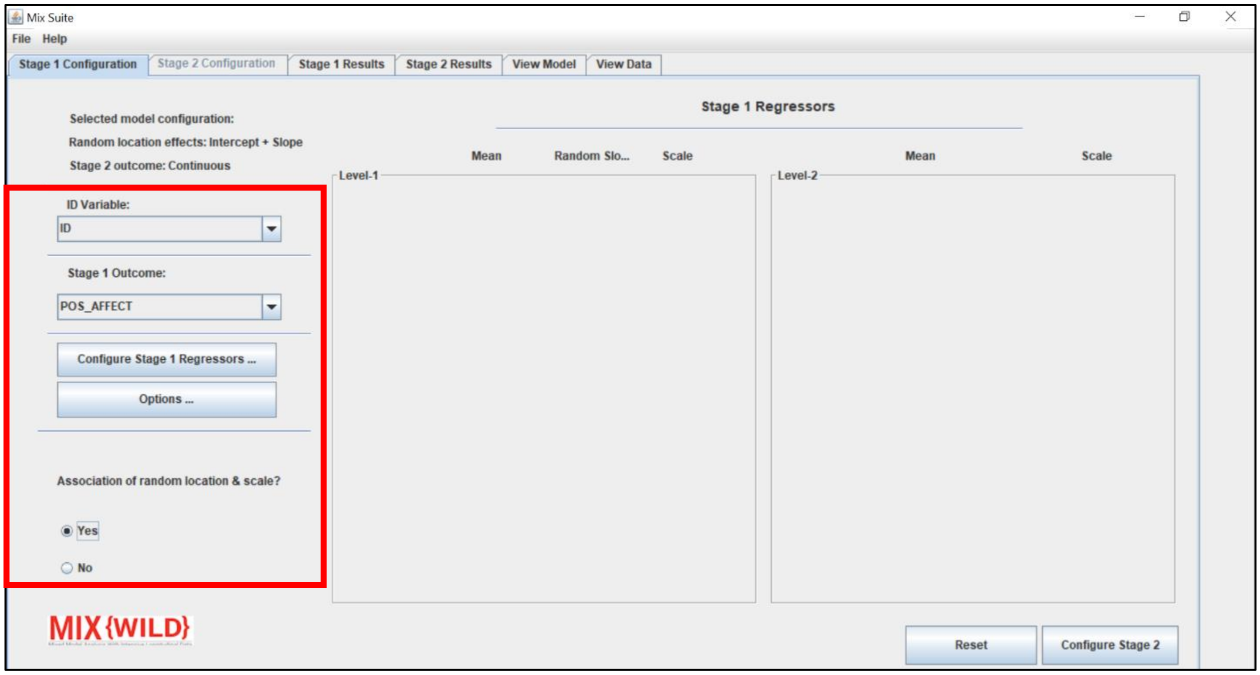

13. Click on **"Modify Stage 1 regressors"** and select time-varying regressor **"WEEKEND"** as the regressor in the Stage 1 model.

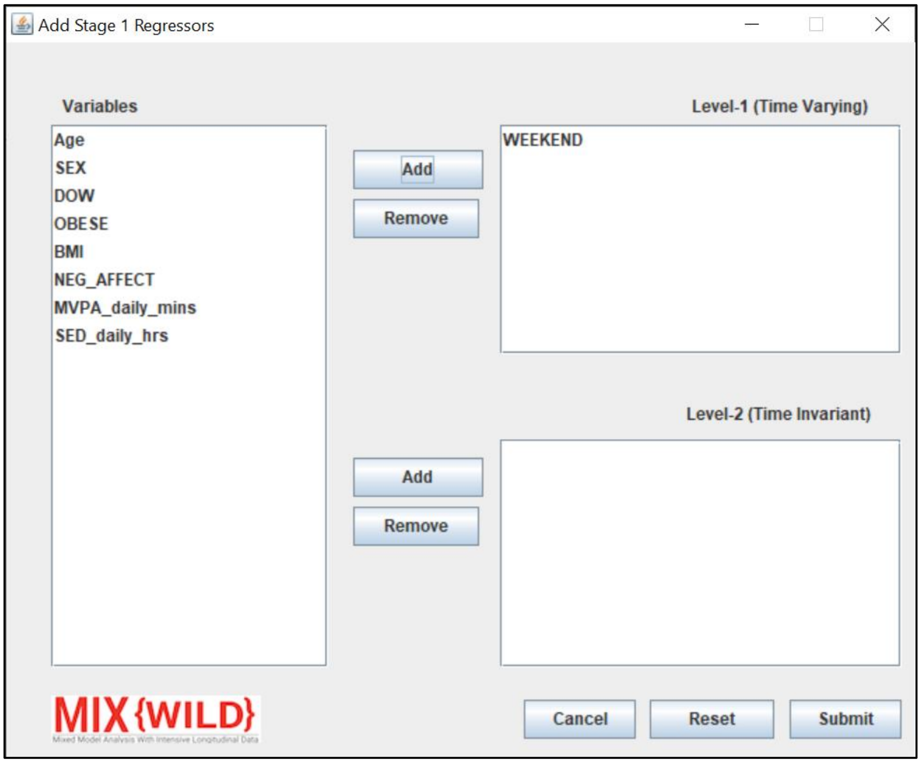

14. Select the boxes in the mean column, BS Variance column, and WS Variance column to allow Stage 1 regressors to predict mean, between-subject variance, and/or within-subject variance of Stage 1 outcome, respectively.
15. In this example, weekend is selected to predict the mean and is allowed to have a random slope on positive affect (i.e., the weekend effect on positive affect will vary across subjects).
16. Select **"Disaggregate"** for each of the time-varying variable(s) for which decomposition of the within-subject and between-subject effects in predicting Stage 1 outcome is desired. Weekend is not disaggregated in this example.

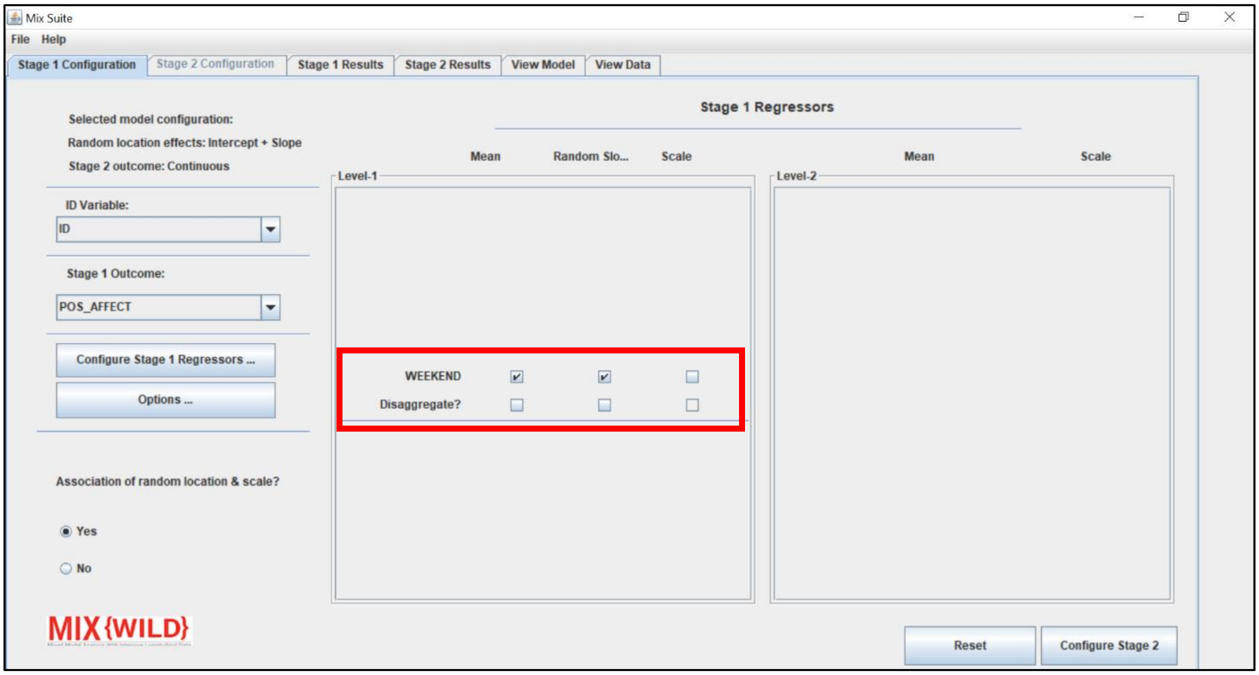

17. Click on **"Options"** to change other default settings if needed.

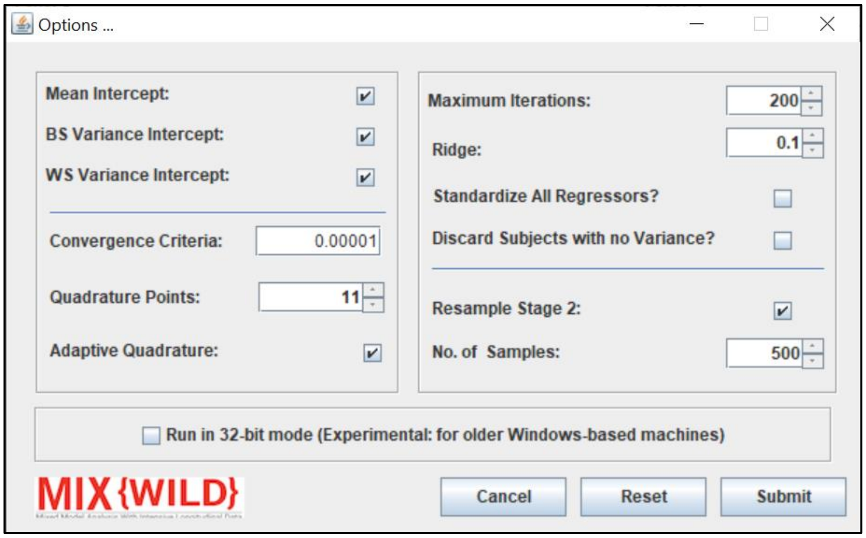

18. In most cases, we will keep Mean, BS and WS intercept checked.
19. By default, the estimated random effects of the Stage 1 analysis (i.e., random location and scale effects) are resampled 500 times in the Stage 2 analysis. Resampling is necessary because the random effects are estimated quantities that are entered as regressors in the Stage 2 model.
20. Leave **"Discard Subjects"** unchecked, to retain all participants in the Stage 1 analysis. Click on **"Submit"**. (refer to the supplemental documents for further description of Options settings)

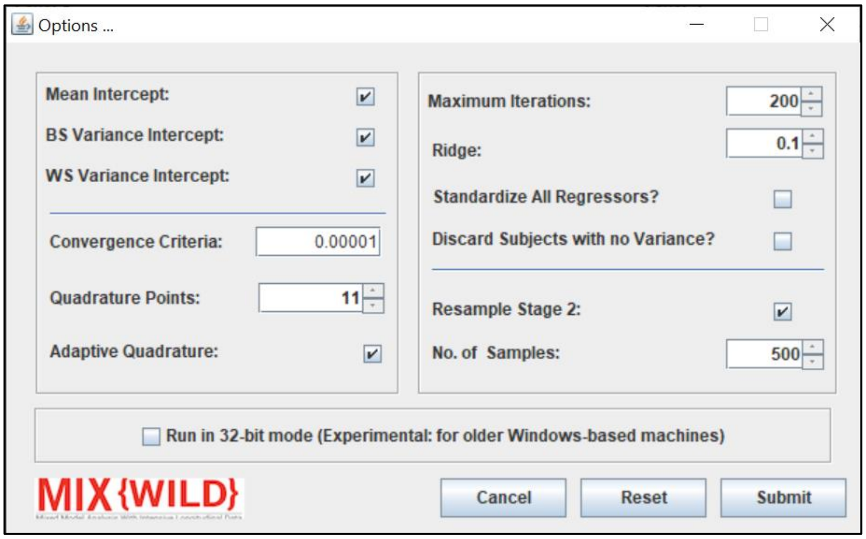

21. Select "**MVPA_daily_minutes"** as Stage 2 outcome variable.

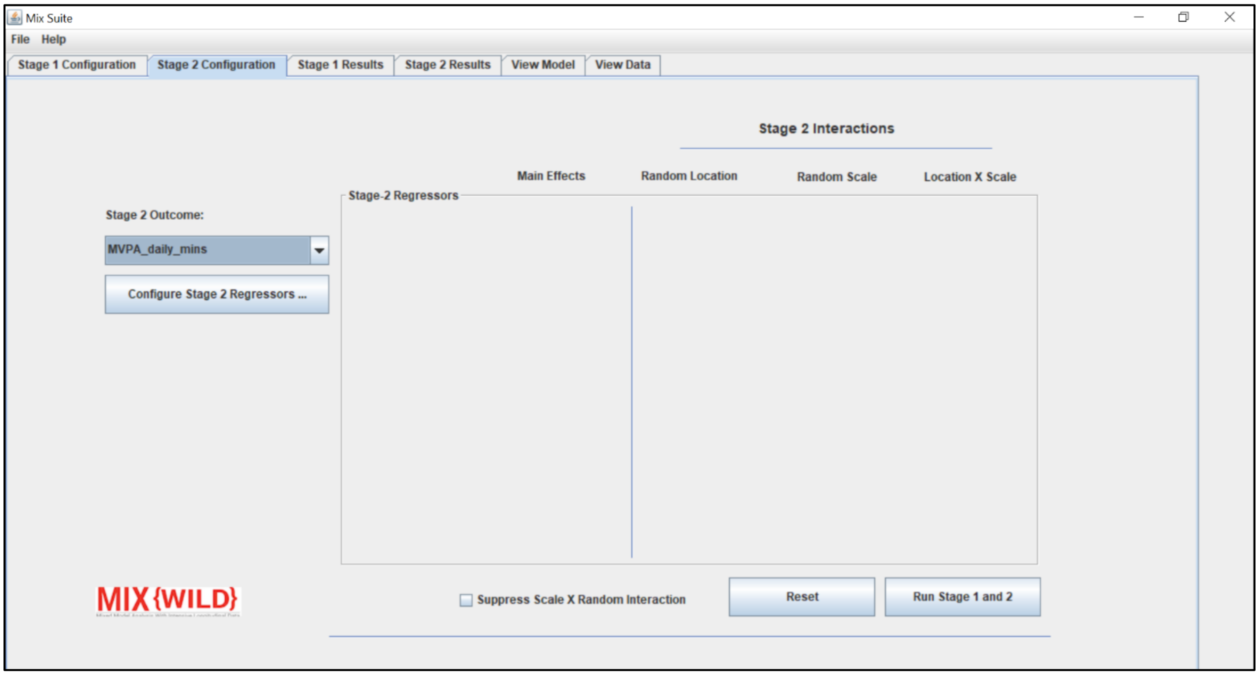

22. Add Stage 2 regressor **"BMI"** into the model. Stage 2 regressor(s) are generally time-invariant variable(s). However, if they are time-varying variables, the program will calculate subject averages of the variable. Click on **"Submit"** when finished.

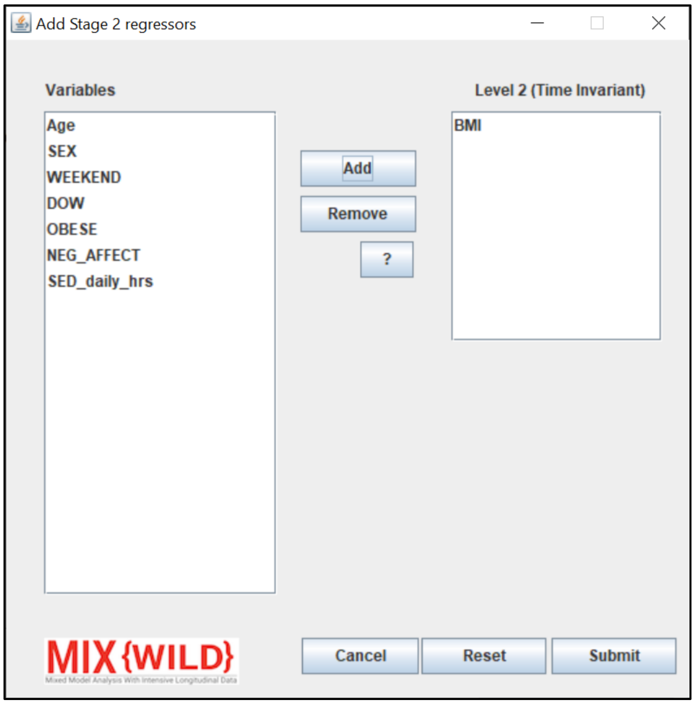

23. Select to add main effect of BMI in predicting daily MVPA minutes. In this example, the interactions between BMI and random location or random slope were not tested, so the remaining boxes are unchecked.

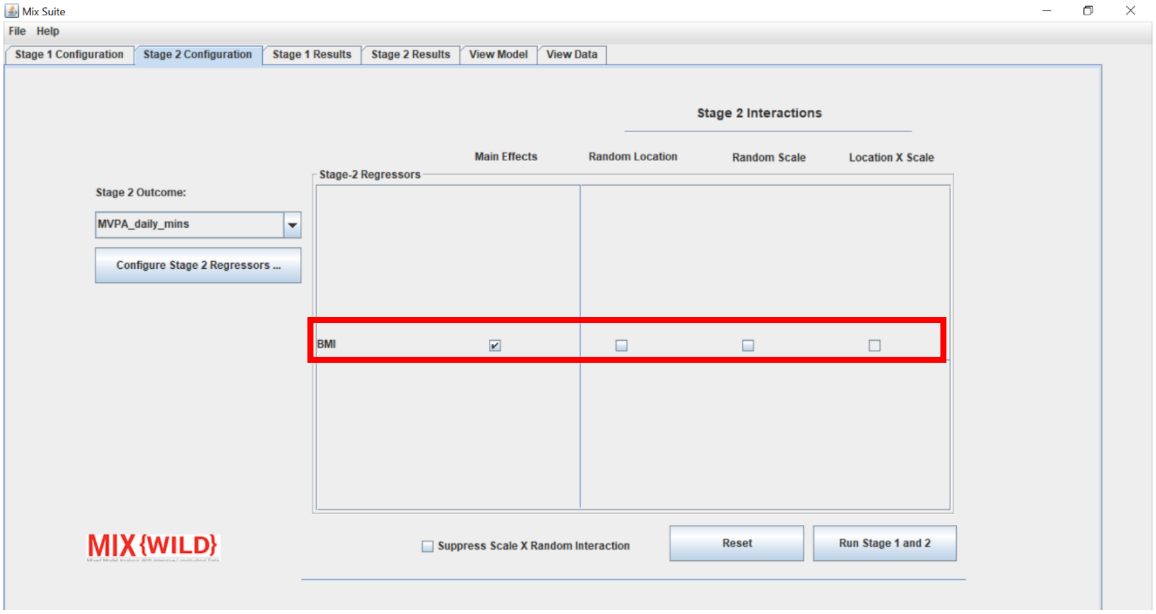

24. Click on Stage 1 and Stage 2 configurations to double-check your model Click on **“Run Stage 1 and 2”** to generate the definition file. The definition file contains the syntax that instructs the program to estimate the specified model.
25. In the definition file, click on **“Proceed**” to run your model and generate model output files. If you see a field with the text **“null”**, it means that there is a mistake in your model that should be fixed.

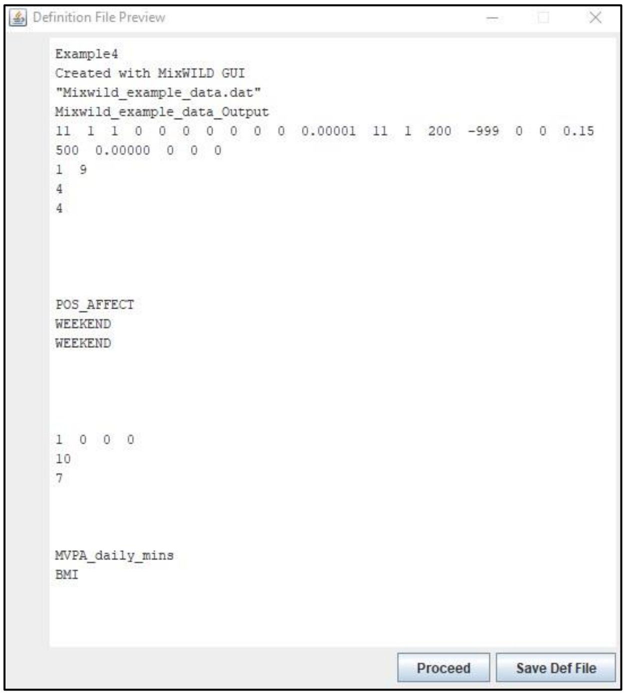

26. A window will appear while model estimation is in progress. The time for generating the final output depends on the data size and complication of your specified model, the run time to estimate a model including random slope could range up to 20 minutes or longer.

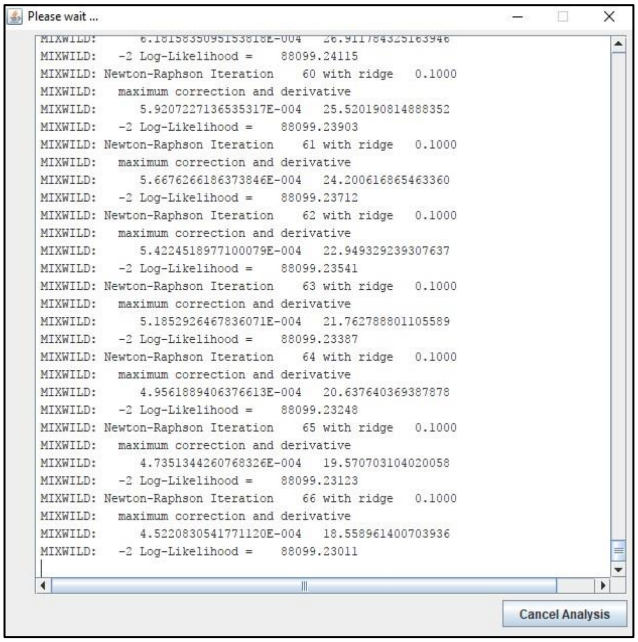

27. If the following warning message appears, it indicates that computational difficulties were encountered that prevented the model parameters from being estimated successfully. In this case, double-check the format of your dataset and your model specifications. Some suggestions for steps to take are listed in Appendix A.

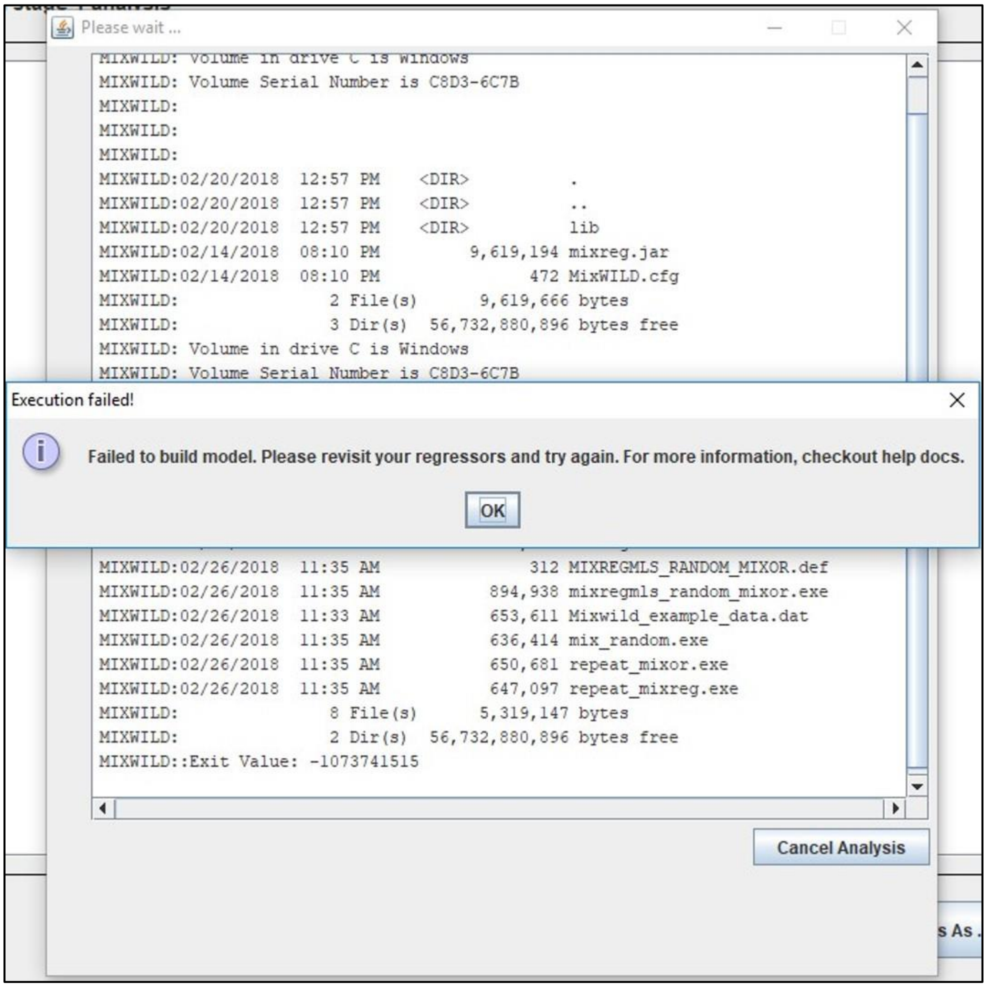

28. When estimation is completed, the Stage 1 and Stage 2 results can be seen by clicking the Stage 1 and Stage 2 Results boxes, respectively.

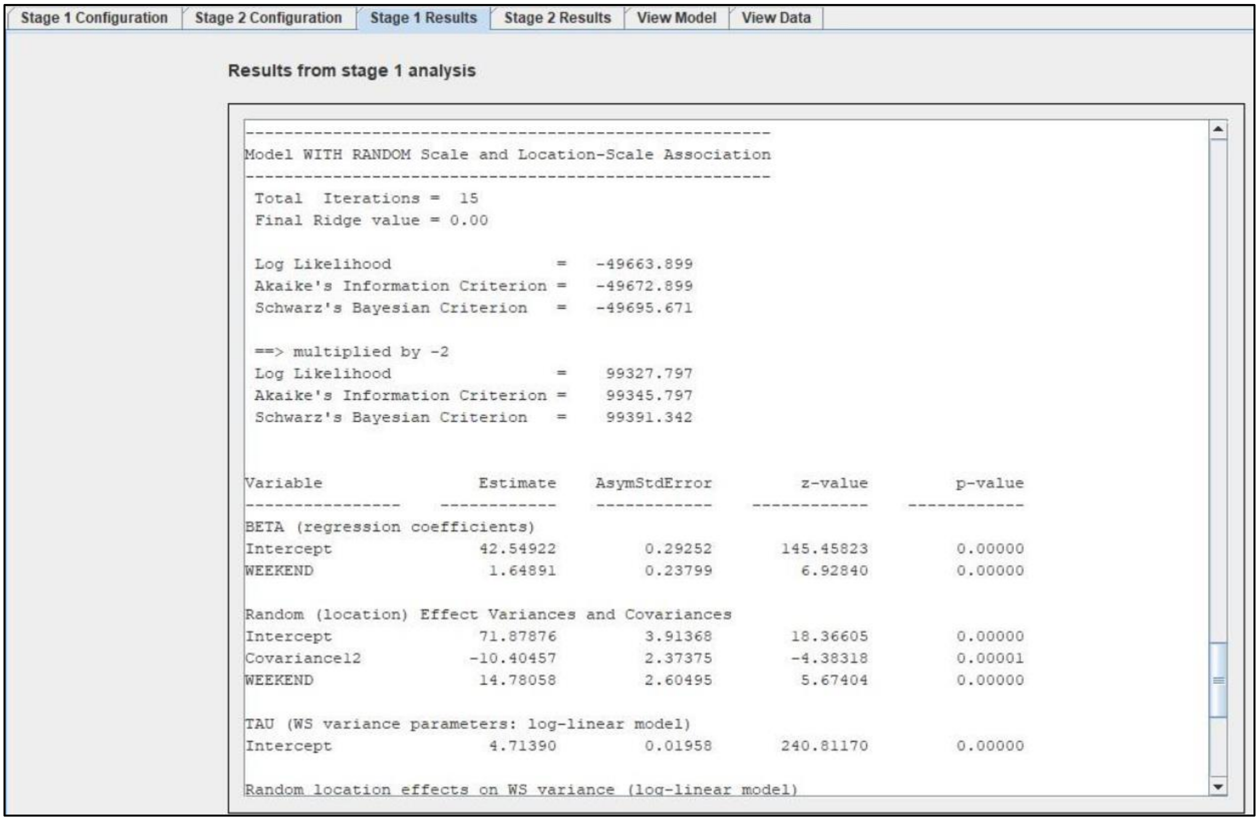


```
--------------------------------------------------------
Model WITH RANDOM Scale and Location-Scale Association
--------------------------------------------------------
 Total  Iterations =  15
 Final Ridge value = 0.00

 Log Likelihood                 =   -49663.899
 Akaike's Information Criterion =   -49672.899
 Schwarz's Bayesian Criterion   =   -49695.671

 ==> multiplied by -2
 Log Likelihood                 =    99327.797
 Akaike's Information Criterion =    99345.797
 Schwarz's Bayesian Criterion   =    99391.342


Variable                Estimate    AsymStdError        z-value         p-value
----------------    ------------    ------------    ------------    ------------
BETA (regression coefficients)
Intercept               42.54922         0.29252       145.45823         0.00000
WEEKEND                  1.64891         0.23799         6.92840         0.00000

Random (location) Effect Variances and Covariances
Intercept               71.87876         3.91368        18.36605         0.00000
Covariance12           -10.40457         2.37375        -4.38318         0.00001
WEEKEND                 14.78058         2.60495         5.67404         0.00000

TAU (WS variance parameters: log-linear model)
Intercept                4.71390         0.01958       240.81170         0.00000

Random location effects on WS variance (log-linear model)
```

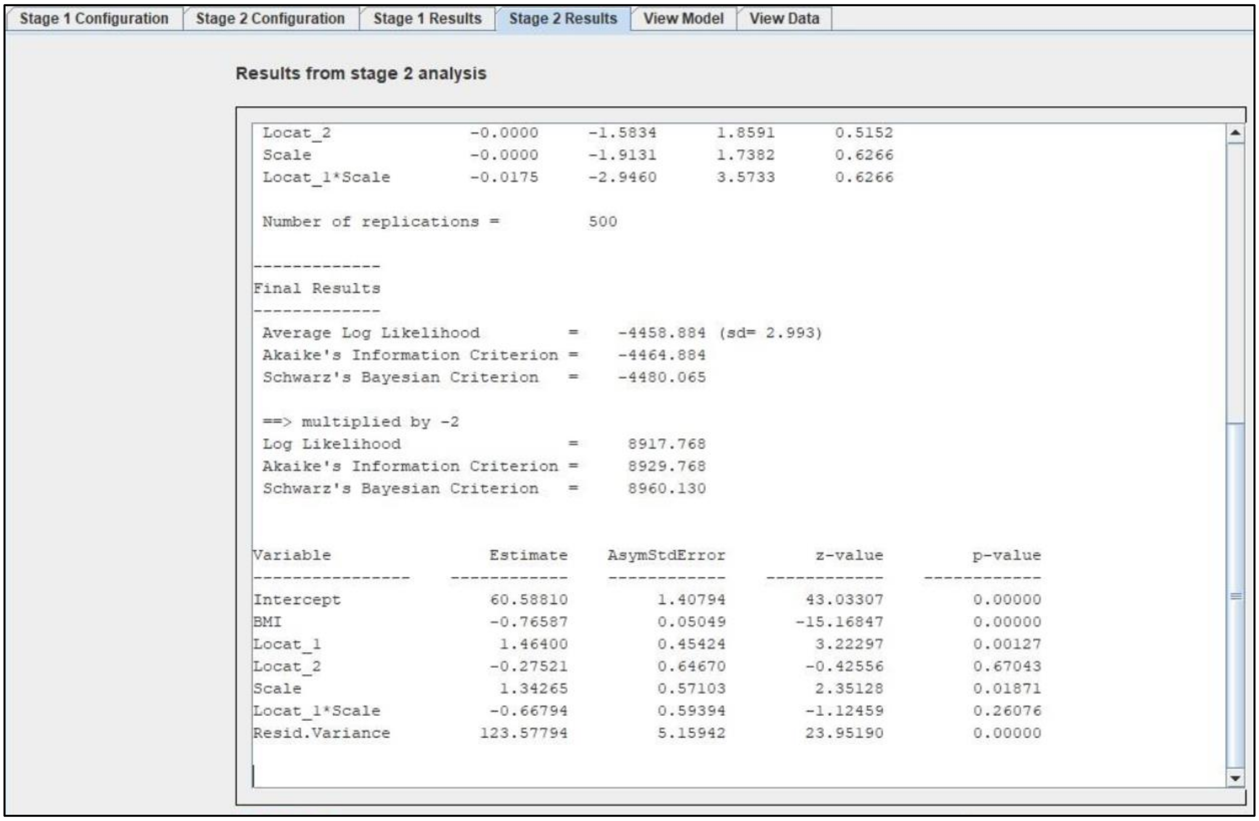


```
 Locat_2          -0.0000     -1.5834     1.8591     0.5152
 Scale            -0.0000     -1.9131     1.7382     0.6266
 Locat_1*Scale    -0.0175     -2.9460     3.5733     0.6266

 Number of replications =         500

-------------
Final Results
-------------
 Average Log Likelihood         =    -4458.884 (sd= 2.993)
 Akaike's Information Criterion =    -4464.884
 Schwarz's Bayesian Criterion   =    -4480.065

 ==> multiplied by -2
 Log Likelihood                 =     8917.768
 Akaike's Information Criterion =     8929.768
 Schwarz's Bayesian Criterion   =     8960.130


Variable                Estimate    AsymStdError        z-value         p-value
----------------    ------------    ------------    ------------    ------------
Intercept               60.58810         1.40794        43.03307         0.00000
BMI                     -0.76587         0.05049       -15.16847         0.00000
Locat_1                  1.46400         0.45424         3.22297         0.00127
Locat_2                 -0.27521         0.64670        -0.42556         0.67043
Scale                    1.34265         0.57103         2.35128         0.01871
Locat_1*Scale           -0.66794         0.59394        -1.12459         0.26076
Resid.Variance         123.57794         5.15942        23.95190         0.00000
```

29. All files generated from the program can be found in a folder with the prefix MixWILD under the same directory of your dataset.

MIXWILD

Name	Date modified	Type
MIXWILD151988992	3/6/2018 5:39 PM	File folder
Mixwild_example_data	2/26/2018 11:54 AM	Microsoft Excel Comma Separated Values File

30. The OUT files with suffix_1 and _2 are the results that are identical to those in the Stage 1 and Stage 2 boxes.

XWILD > MIXWILD151988992

Name	Date modified	Type	Size
work	4/1/2018 5:06 PM	File folder	
mix_random	2/26/2018 1:12 PM	DEF File	1 KB
mix_random	2/26/2018 12:49 PM	Application	665 KB
mixor	2/26/2018 12:49 PM	Application	914 KB
mixreg	2/26/2018 12:49 PM	Application	967 KB
MIXREGMLS_RANDOM_MIXREG	2/26/2018 12:48 PM	DEF File	1 KB
Mixwild_example_data	3/8/2018 11:42 AM	Microsoft Excel C...	635 KB
Mixwild_example_data	2/26/2018 12:47 PM	DAT File	635 KB
Mixwild_example_data_Output	2/26/2018 12:49 PM	DEF File	1 KB
Mixwild_example_data_Output.mwa	2/26/2018 12:48 PM	MWA File	94 KB
Mixwild_example_data_Output.mwd	2/26/2018 12:48 PM	MWD File	20 KB
Mixwild_example_data_Output_1	2/26/2018 1:12 PM	OUT File	14 KB
Mixwild_example_data_Output_2	2/26/2018 1:13 PM	OUT File	3 KB
Mixwild_example_data_Output_ebrandom	2/26/2018 1:12 PM	DAT File	1,707 KB
Mixwild_example_data_Output_ebvar	2/26/2018 1:12 PM	DAT File	156 KB
Mixwild_example_data_Output_level2	2/26/2018 1:12 PM	DAT File	55 KB
Mixwild_example_data_Output_random	2/26/2018 1:12 PM	DEF File	1 KB
Mixwild_example_data_Output_random_500	2/26/2018 1:13 PM	OUT File	2 KB
Mixwild_example_data_Output_repeat_mixreg	2/26/2018 1:12 PM	DEF File	1 KB
repeat_mixor	2/26/2018 12:49 PM	Application	680 KB
repeat_mixreg	2/26/2018 1:12 PM	DEF File	1 KB
repeat_mixreg	2/26/2018 12:49 PM	Application	677 KB

31. There are 3 submodel results in Output_1. The first submodel does not include scale parameters; the second submodel includes the random scale estimates, and the third submodel includes the random scale estimates and the random location-scale association as follows. (A brief description of results from the following third submodel will be provided)

```
Model WITH RANDOM Scale and Location-Scale Association

 Total  Iterations =  15
 Final Ridge value = 0.00

 Log Likelihood                 =    -49663.899
 Akaike's Information Criterion =    -49672.899
 Schwarz's Bayesian Criterion   =    -49695.671
```

5.1. Brief interpretation of Mixregmls - Linear Regression model results

Stage 1 model with random scale and location-scale association

```
---------------------------------------------------
Model WITH RANDOM Scale and Location-Scale Association
---------------------------------------------------
 Total  Iterations =  15
 Final Ridge value = 0.00


Variable                Estimate     AsymStdError          z-value          p-value
----------------    ------------    ------------    ------------    ------------
BETA (regression coefficients)
Intercept               42.54922         0.29252       145.45823         0.00000
WEEKEND                  1.64891         0.23799         6.92840         0.00000

Random (location) Effect Variances and Covariances
Intercept               71.87876         3.91368        18.36605         0.00000
Covariance12           -10.40457         2.37375        -4.38318         0.00001
WEEKEND                 14.78058         2.60495         5.67404         0.00000

TAU (WS variance parameters: log-linear model)
Intercept                4.71390         0.01958       240.81170         0.00000

Random location effects on WS variance (log-linear model)
Intercept               -0.12200         0.02285        -5.33863         0.00000
WEEKEND                 -0.03975         0.03458        -1.14954         0.25033

Random scale standard deviation
Std Dev                  0.40098         0.02108        19.01857         0.00000
```

- The Stage 1 model shows that weekend is positively associated with positive affect (estimate=1.6489, *p*<.001). Individuals overall have higher mean positive affect on weekend days relative to weekdays.
- There is significant variability in both intercept (i.e., random intercept) and slope (i.e., random slope) across subjects. The random intercept is estimated as 71.8788 on the log scale (*p*<.001) and the random slope is estimated as 14.7808 on the log scale (*p*<.001). Therefore, individuals differ from each other in their mean levels of positive affect and in their associations between weekday/weekend and momentary positive affect.
- The random intercept and random slope are negatively associated with each other (estimate= -10.4045, *p*<.001). This negative covariance indicates that subjects with higher weekday mean levels of positive affect (i.e., higher levels of the intercept) do not increase in positive affect as much on weekends, relative to subjects with lower weekday positive affect.
- For the relationships between the random location and scale effects, the random intercept (i.e., weekday positive affect) is negatively associated with the WS variance (estimated as -0.1220 on the log scale (*p*<.001). This indicates that subjects with higher weekday positive affect are more consistent/less erratic in their mood reports. A subject's random slope (positive affect change on weekend days relative to weekdays) is not significantly related to a subject's WS variance.
- There is significant variability in scale across subjects, as the Std Dev for the Random scale is estimated as 0.4010 on the log scale (*p*<.001). Thus, individuals differ from each other in their degree of within-subject/intraindividual variability in positive affect.

Stage 2 model with continuous subject-level outcome

```
-------------
Final Results
-------------
 Average Log Likelihood          =    -4458.884 (sd= 2.993)
 Akaike's Information Criterion =    -4464.884
 Schwarz's Bayesian Criterion   =    -4480.065

 ==> multiplied by -2
 Log Likelihood                  =     8917.768
 Akaike's Information Criterion =     8929.768
 Schwarz's Bayesian Criterion   =     8960.130
```

Variable	Estimate	AsymStdError	z-value	p-value
Intercept	60.58810	1.40794	43.03307	0.00000
BMI	-0.76587	0.05049	-15.16847	0.00000
Locat_1	1.46400	0.45424	3.22297	0.00127
Locat_2	-0.27521	0.64670	-0.42556	0.67043
Scale	1.34265	0.57103	2.35128	0.01871
Locat_1*Scale	-0.66794	0.59394	-1.12459	0.26076
Resid.Variance	123.57794	5.15942	23.95190	0.00000

- In the Stage 2 final results table, *Locat_1* refers to the effect of the random intercept (i.e., within-subject mean) on MVPA minutes; *Locat_2* refers to the effect of the random slope (i.e., within-subject association between weekday/weekend and positive affect) on MVPA minutes*; Scale* refers to the effect of random scale (i.e., within-subject variance) on MVPA minutes; and *Locat_1* Scale* is the interaction between random intercept and random scale predicting MVPA minutes.
- Results show that after controlling for other variables, subjects' BMI is negatively associated with mean MVPA minutes (estimate= -0.7659, p<.001).
- The random intercept (*Locat_1*) positively predicts MVPA minutes when the random scale is zero (estimate= 1.1460, p<.001). Since the random effects are centered around zero, a random scale of zero represents the average scale. For subjects with average scale of positive affect, higher levels of weekday positive affect are associated with higher daily MVPA minutes.
- The random scale (*Scale*) is significant in predicting MVPA minutes when the random intercept (*Locat_1*) is zero (estimate=1.3427, p<.05). Since the random effects are centered around zero, a random intercept of zero represents the average positive affect level on weekdays. For subjects with average weekday levels of positive affect, higher levels of mood variability are associated with higher averaged daily MVPA minutes.

Note:

1. The numbers of level 1 and level 2 observations correspond to the available non-missing observations of level 1 and level 2 variables included in both Stage 1 and Stage 2 models. Thus, these analyses are carried out using observations that are non-missing in terms of both outcomes (positive affect and averaged daily MVPA) and regressors (weekend and BMI). Refer to the supplemental documents for more detailed explanation of the Stage 1 and Stage 2 model results.

6. Example 5: Running Mixregmls - Logistic regression model in MixWILD

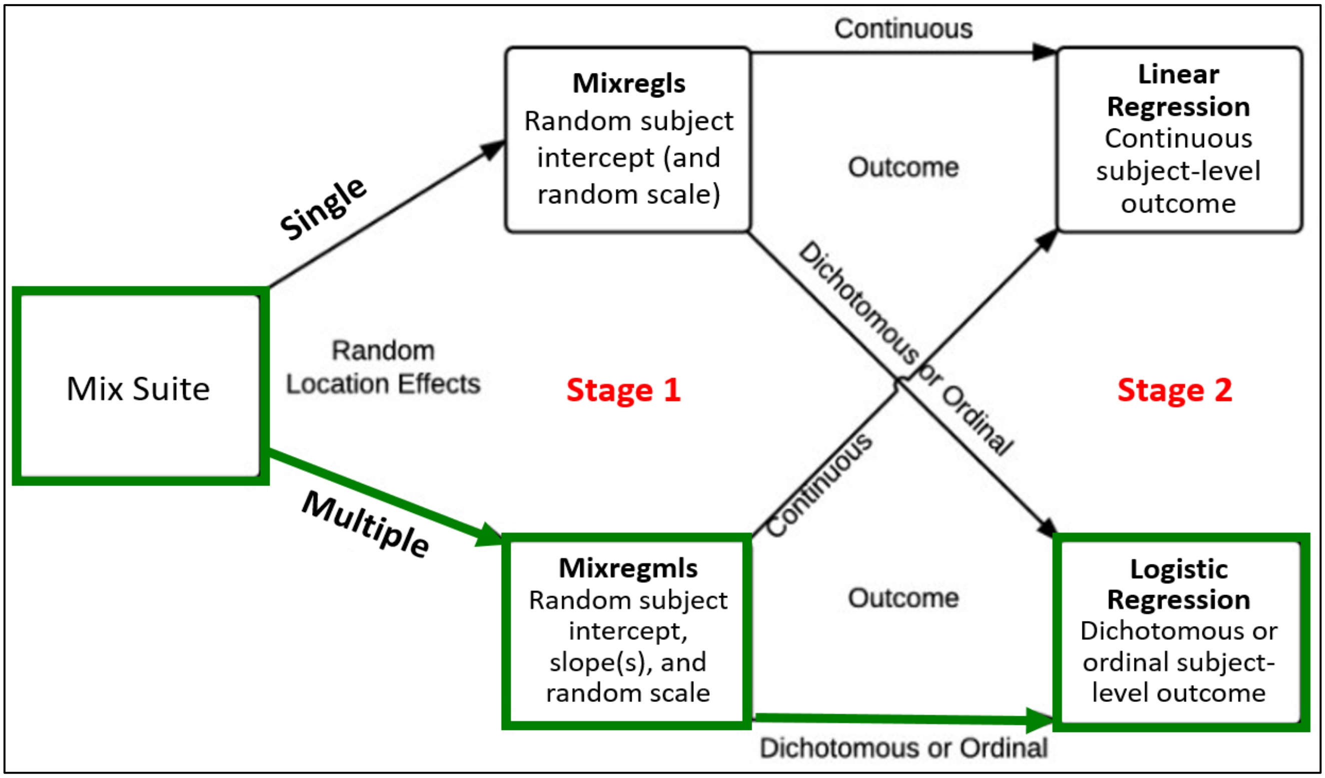

Example research questions for Mixregmls-Logistic Regression model

- Examine whether subject-level mean (i.e., random intercept or within-person mean) and subject-level variance (i.e., random scale or degree of within-person / intraindividual variability) of momentary positive affect (within-subject, continuous, time-varying variable) predicts subject-level obesity risk (between-subject, dichotomous, time-invariant variable), in addition to their positive affect change between weekdays and weekends (within-subject, dichotomous, time-varying variable), Sex, and Age (between-subject, continuous, time-invariant variable).
- Examine whether there is significant variability between individuals in the association (i.e., random slope) between weekday/weekend and momentary positive affect (i.e., whether individuals differ from each other in the extent to which positive affect increases towards the end of the week), after controlling for subject-level mean and subject-level variance.
- Examining whether the variability between individuals in the association (i.e., random slope) between weekday/weekend and momentary positive affect predicts subject-level obesity risk, after controlling for Age.
- Examining whether participants' Age could moderate the associations between mean levels (i.e., random intercept) and variances (i.e., random scale) in positive affect in predicting obesity risk, and whether participants' Age could moderate weekend-positive affect association (i.e., random slope) in predicting obesity risk.

Stage 1 outcome variable: Positive affect (time varying)
Stage 1 regressor (with random slope): Weekend vs. Weekday (time varying); Sex (time invariant)
Stage 2 outcome variable: Obese or Non-obese (time invariant)
Stage 2 regressors: Age (time invariant); Age x random intercept interaction; Age x random scale interaction; Age x random intercept x random scale interaction

6.1. Step-by-step instructions for running a Mixregmls- Logistic regression model in MixWILD

1. Double-click on the MixWILD icon to open the main window.
2. Click on **"File"** and then select **"New Model"** (or use keyboard shortcut Ctrl + N).

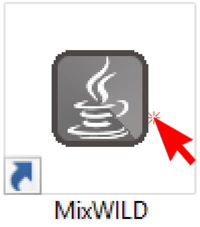

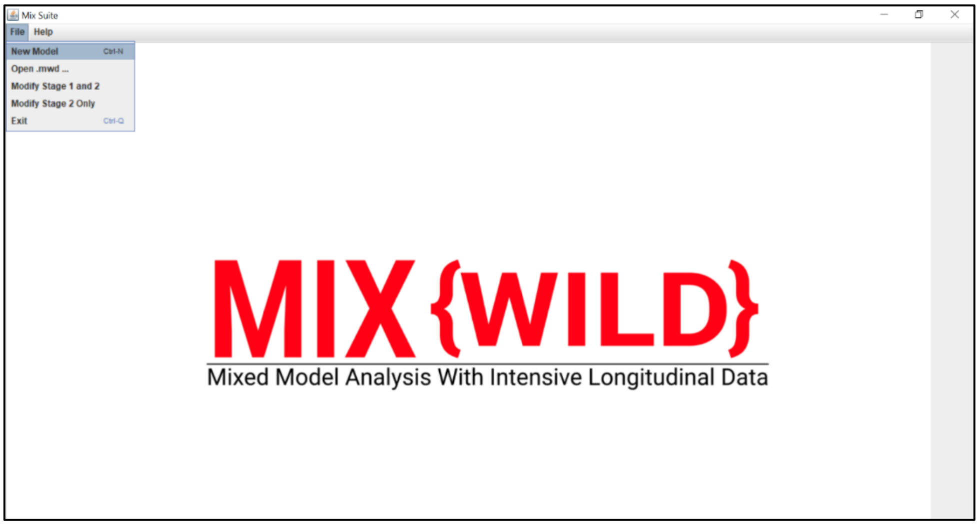

3. Click on **"Instructions"** to make sure your data are in the correct format.

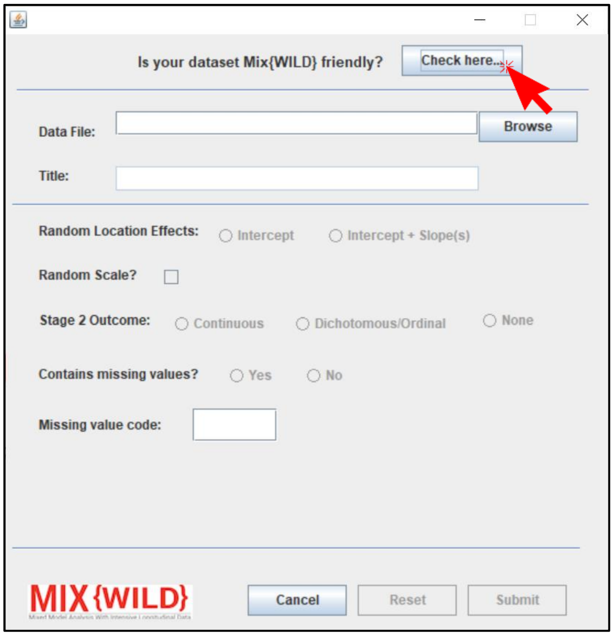

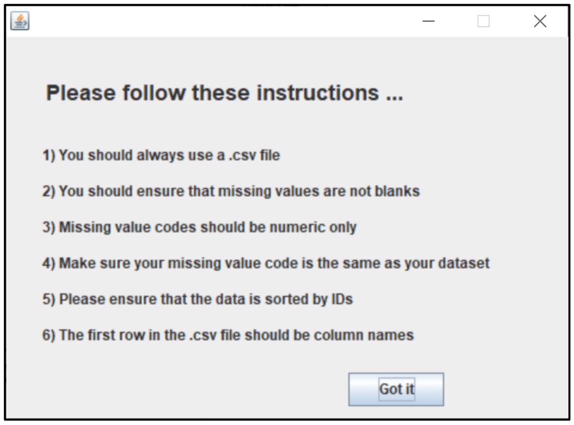

4. Click on **“Browse”** to select the location of your data file (in .csv) and then click **“Open”**.

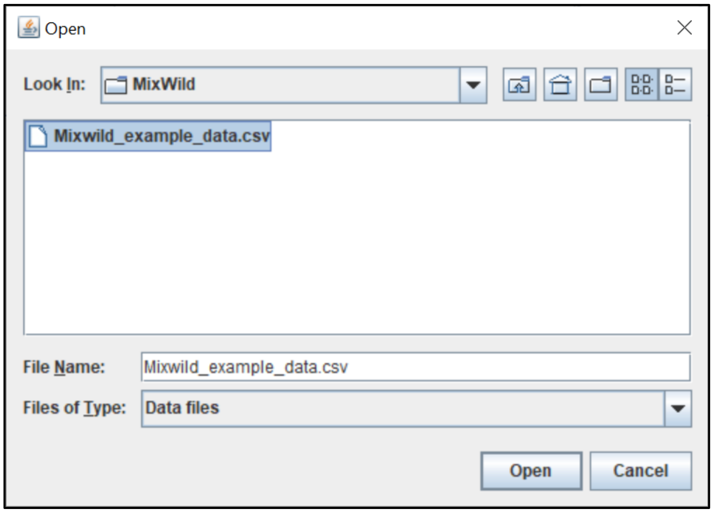

5. Click on **“View Data”** to preview your data file to verify your data and format are correct.

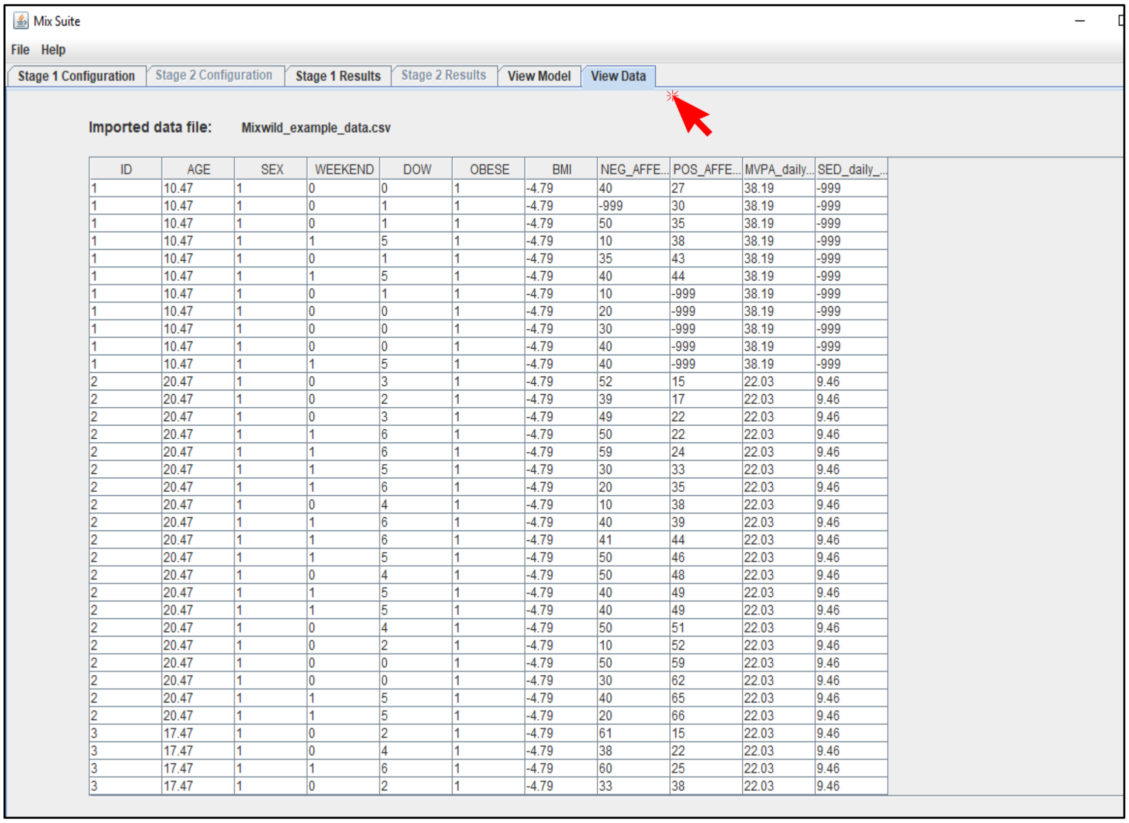

ID	AGE	SEX	WEEKEND	DOW	OBESE	BMI	NEG_AFFE...	POS_AFFE...	MVPA_daily...	SED_daily_...
1	10.47	1	0	0	1	-4.79	40	27	38.19	-999
1	10.47	1	0	1	1	-4.79	-999	30	38.19	-999
1	10.47	1	0	1	1	-4.79	50	35	38.19	-999
1	10.47	1	1	5	1	-4.79	10	38	38.19	-999
1	10.47	1	0	1	1	-4.79	35	43	38.19	-999
1	10.47	1	1	5	1	-4.79	40	44	38.19	-999
1	10.47	1	0	1	1	-4.79	10	-999	38.19	-999
1	10.47	1	0	0	1	-4.79	20	-999	38.19	-999
1	10.47	1	0	0	1	-4.79	30	-999	38.19	-999
1	10.47	1	0	0	1	-4.79	40	-999	38.19	-999
1	10.47	1	1	5	1	-4.79	40	-999	38.19	-999
2	20.47	1	0	3	1	-4.79	52	15	22.03	9.46
2	20.47	1	0	2	1	-4.79	39	17	22.03	9.46
2	20.47	1	0	3	1	-4.79	49	22	22.03	9.46
2	20.47	1	1	6	1	-4.79	50	22	22.03	9.46
2	20.47	1	1	6	1	-4.79	59	24	22.03	9.46
2	20.47	1	1	5	1	-4.79	30	33	22.03	9.46
2	20.47	1	1	6	1	-4.79	20	35	22.03	9.46
2	20.47	1	0	4	1	-4.79	10	38	22.03	9.46
2	20.47	1	1	6	1	-4.79	40	39	22.03	9.46
2	20.47	1	1	6	1	-4.79	41	44	22.03	9.46
2	20.47	1	1	5	1	-4.79	50	46	22.03	9.46
2	20.47	1	0	4	1	-4.79	50	48	22.03	9.46
2	20.47	1	1	5	1	-4.79	40	49	22.03	9.46
2	20.47	1	1	5	1	-4.79	40	49	22.03	9.46
2	20.47	1	0	4	1	-4.79	50	51	22.03	9.46
2	20.47	1	0	2	1	-4.79	10	52	22.03	9.46
2	20.47	1	0	0	1	-4.79	50	59	22.03	9.46
2	20.47	1	0	0	1	-4.79	30	62	22.03	9.46
2	20.47	1	1	5	1	-4.79	40	65	22.03	9.46
2	20.47	1	1	5	1	-4.79	20	66	22.03	9.46
3	17.47	1	0	2	1	-4.79	61	15	22.03	9.46
3	17.47	1	0	4	1	-4.79	38	22	22.03	9.46
3	17.47	1	1	6	1	-4.79	60	25	22.03	9.46
3	17.47	1	0	2	1	-4.79	33	38	22.03	9.46

6. Add title to your output files. Select **"Intercept + Slope"** from Random Location Effects specification for subject-level mean and subject-level slope. Check **"Random Scale"** for subject-level variability.
7. Select **"Dichotomous/Ordinal"** for the Stage 2 outcome.
8. Click on missing values if there are any in your dataset; specify your missing value code in the box (i.e., -999 in the example dataset).

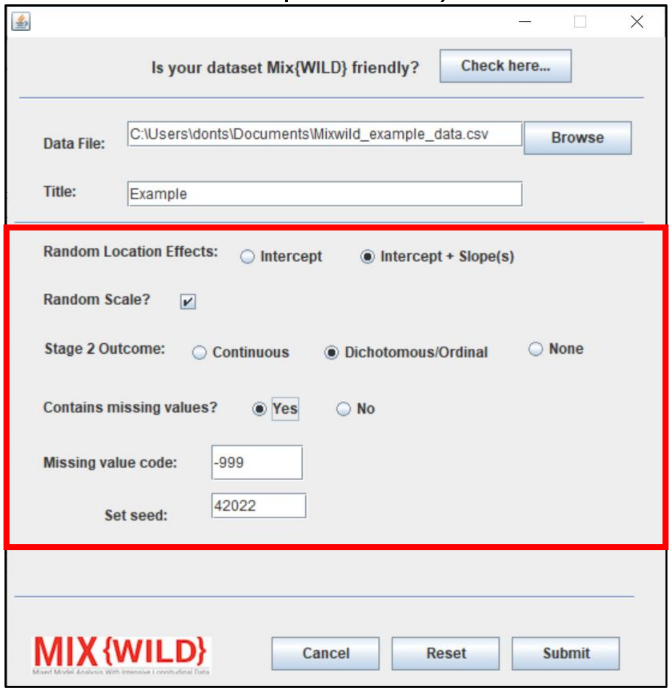

9. After you submit, the interface will take you to the page that enables you to configure your Stage 1 model.
10. Select ID variable and positive affect as your Stage 1 outcome variable.

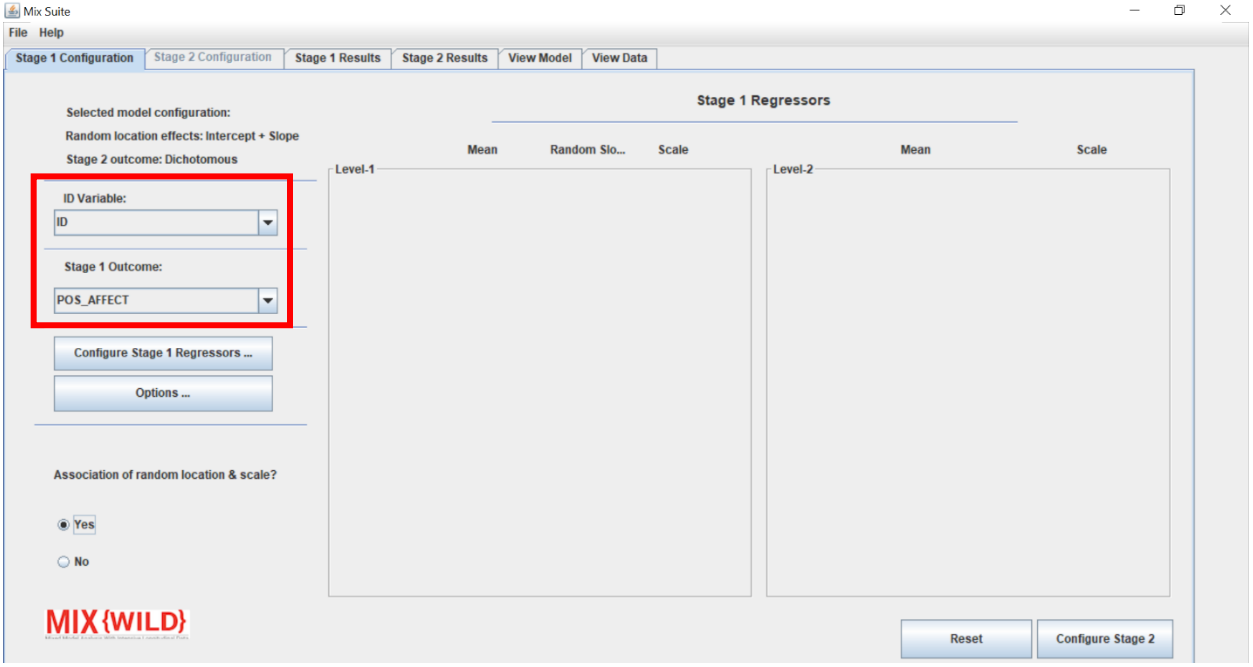

11. Specify the association between the mean and within- subject (WS) variance, which is the association of the random location and random scale effects. The default is no association, but **"Yes"** is selected for the following example.
12. Click on **"Modify Stage 1 regressors"**. Select **"Weekend"** for time-varying regressor and select **"Sex"** as a time-invariant regressor and click on **"Submit"**.

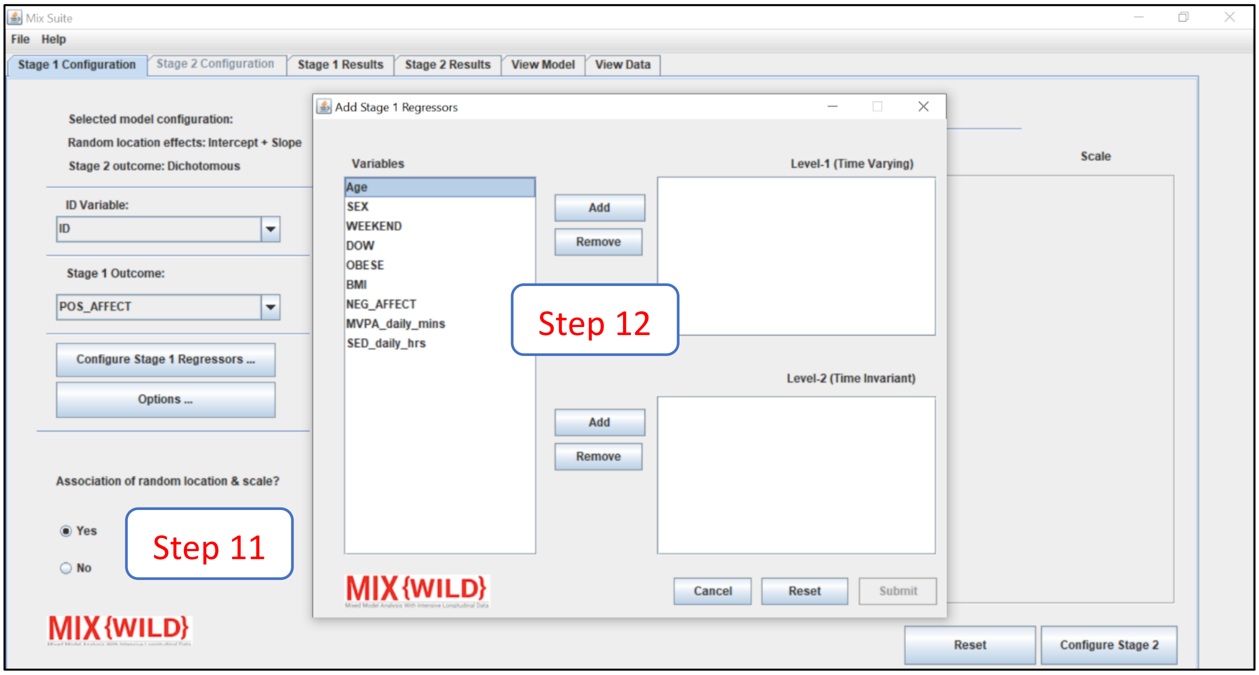

13. Select the boxes in the mean column, random slope, and scale column to allow Stage 1 regressors to predict the mean, estimate random slope, and/or random scale of Stage 1 outcome, respectively. In this example, weekend will be included in the mean model and allow for it to have a random slope (i.e., the positive affect change between weekdays and weekends will be allowed to vary across subjects).

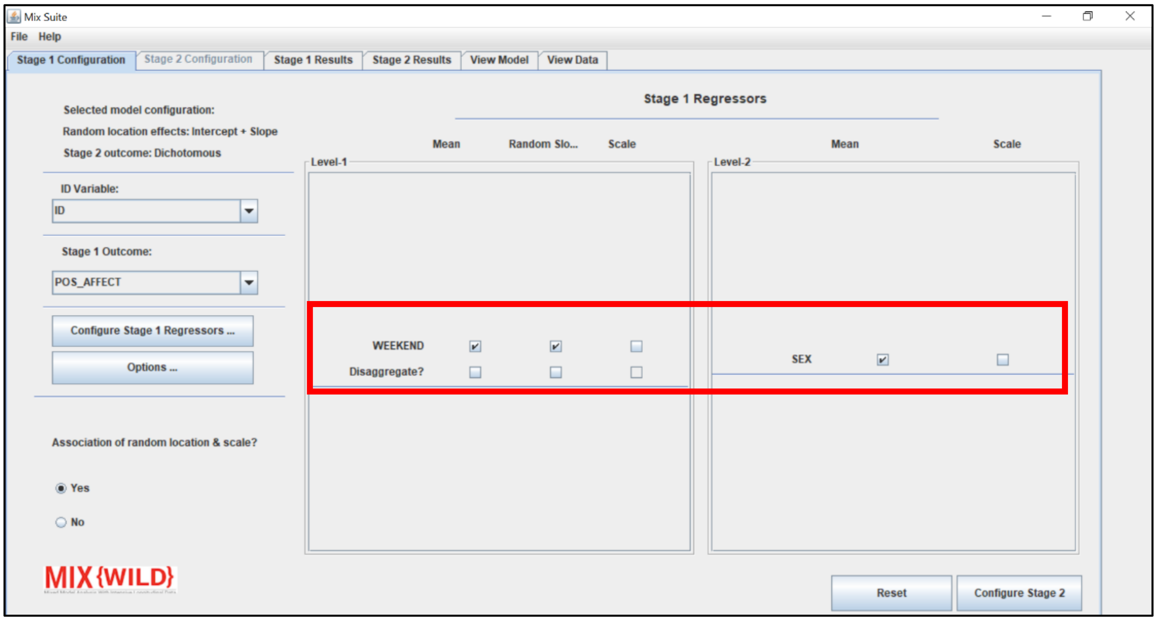

14. Select **"Disaggregate"** for each of the time-varying variable(s) for which decomposition of the within-subject and between-subject effects in predicting Stage 1 outcome is desired. In this case, we will not disaggregate the Weekend variable.

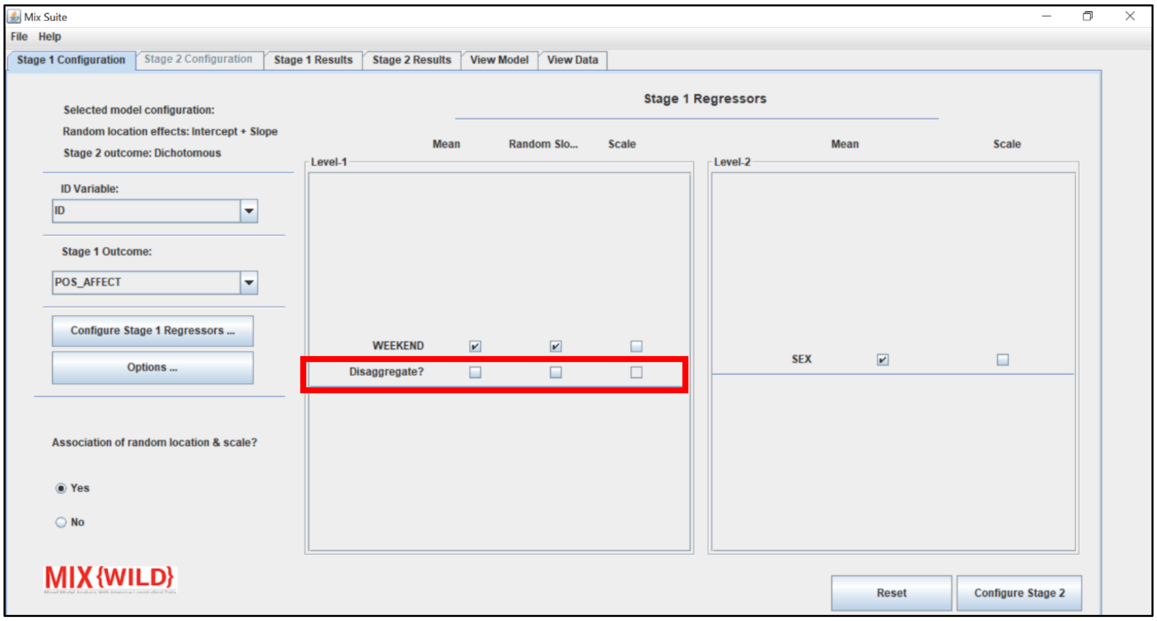

15. Click on **"Options"** to change other default settings if needed. In most cases, we will keep Mean, BS and WS intercept checked.

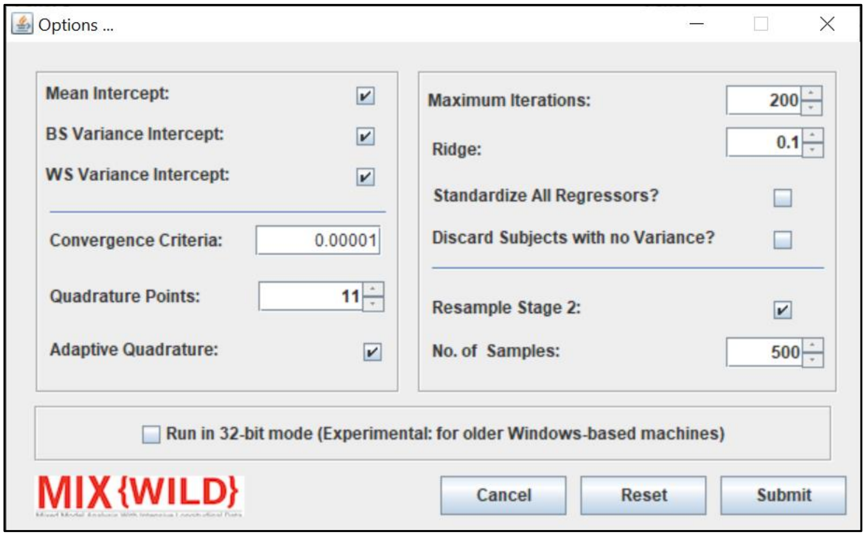

16. By default, the estimated random effects of the Stage 1 analysis (i.e., random location and scale effects) are resampled 500 times in the Stage 2 analysis. Resampling is necessary because the random effects are estimated quantities that are entered as regressors in the Stage 2 model.
17. Leave **"Discard Subjects"** unchecked, so we do not drop participants who have 0 variation in their Stage 1 outcome variable. Click on **"Submit"**

(Refer to the supplemental documents for further description of Options settings)

18. Select **“Obese”** as Stage 2 dichotomous outcome variable in Stage 2 configuration page. Check if the outcome categories are correct.
19. Click on **“Configure Stage 2 Regressors”** to add regressor(s).

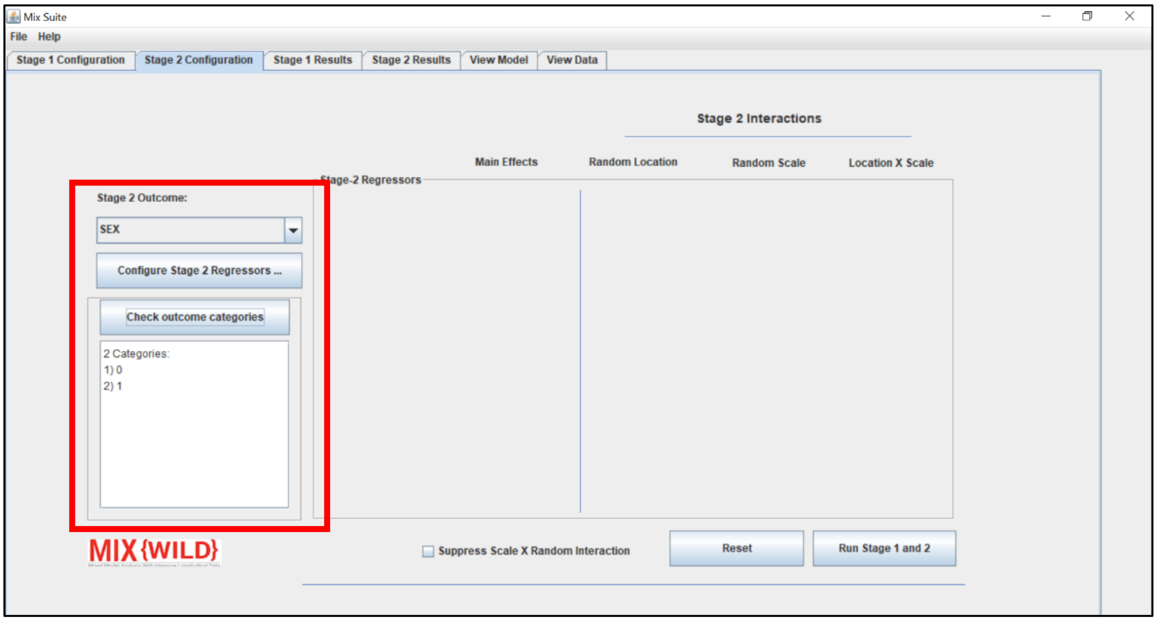

20. Select **“Age”** as a regressor in the Stage 2 model. Stage 2 regressor(s) are generally time-invariant variable(s). However, if they are time-varying variables, the program will calculate subject averages of the variable. Click on **“Submit”** when finished.

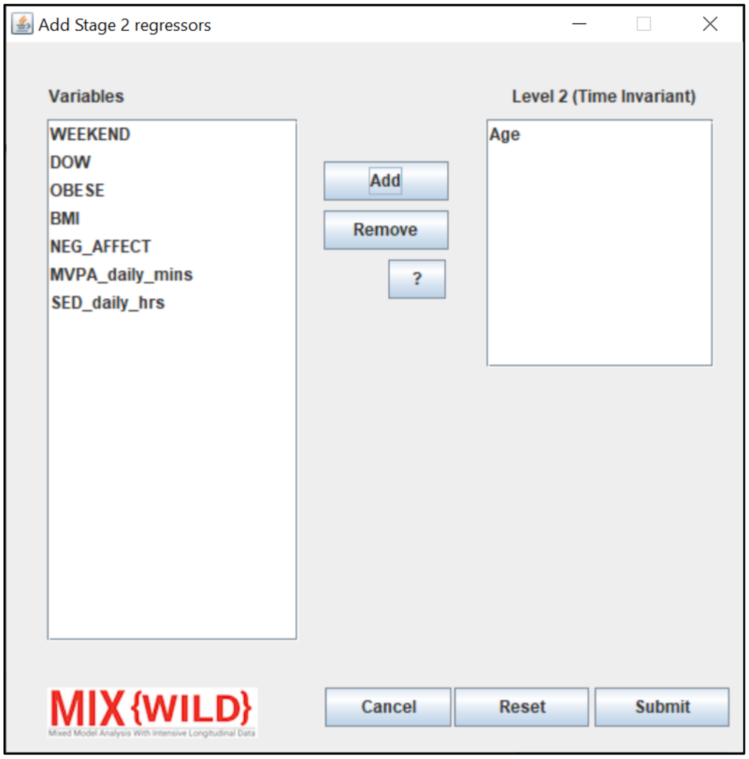

21. Select to add Stage 2 main effect(s) and interaction effect(s) with Stage 1 random effects as regressors.

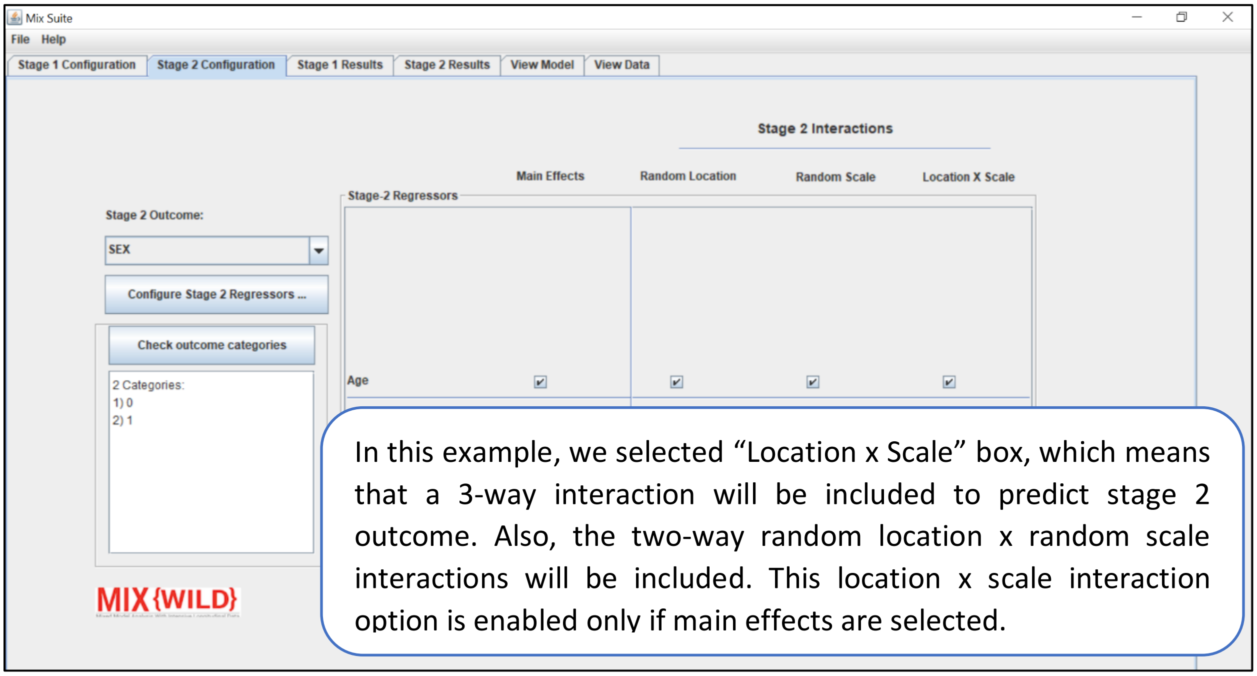

22. Click on Stage 1 and Stage 2 configurations to double-check your model. Click on "Run Stage 1 and 2" to generate the definition file.

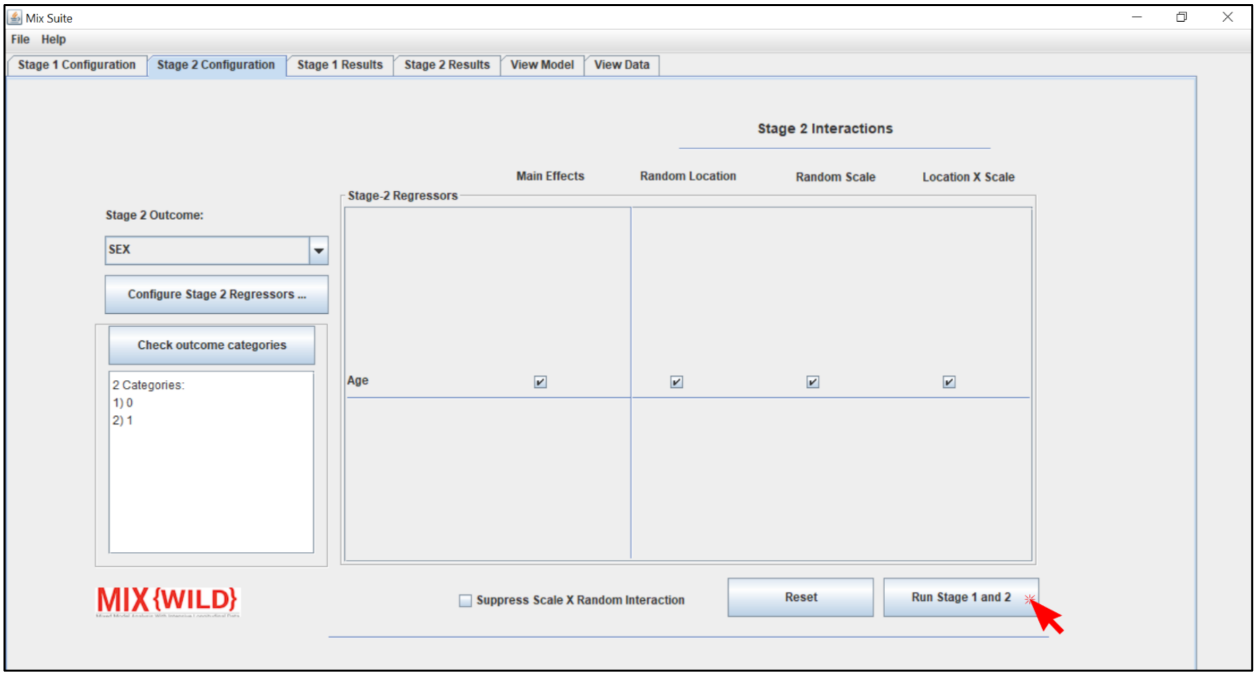

23. The definition file contains the syntax that instructs the program to estimate the specified model. In the definition file, click on **"Proceed"** to run your model and generate model output files.

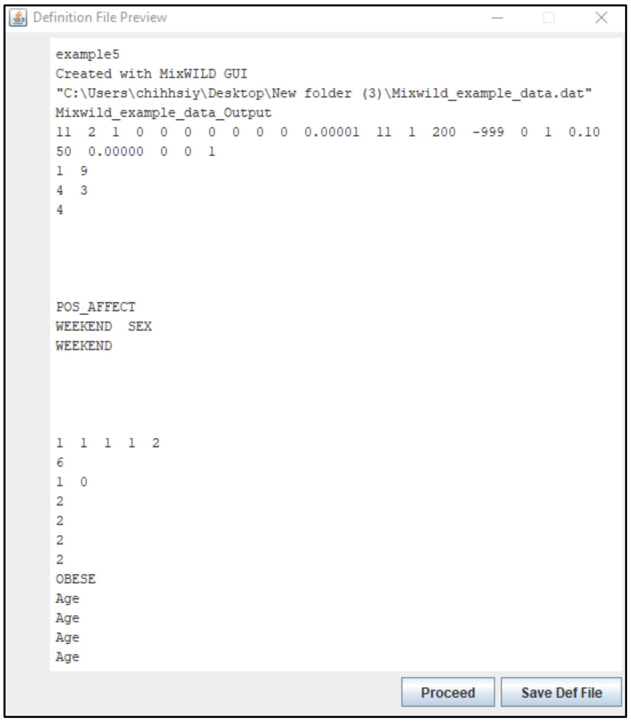

24. A window will appear while model estimation is in progress. The time for generating the final output depends on the size of your dataset and the complication of your specified model, especially in the Stage 1 model. The run time to estimate a model including random slope could range up to 20 minutes or longer.

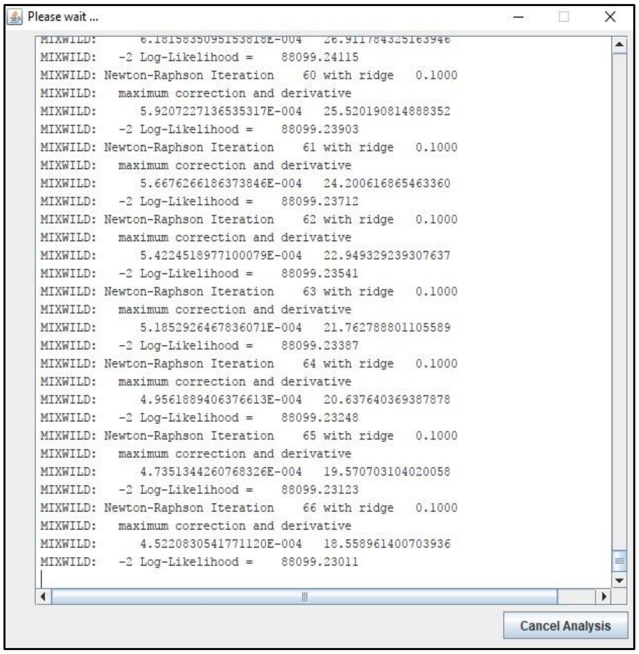

25. If the following warning message appears, it indicates that computational difficulties were encountered and prevented the model parameters from being estimated successfully. In this case, double-check the format of your dataset and your model specifications. Some suggestions for steps to take are listed in Appendix A.

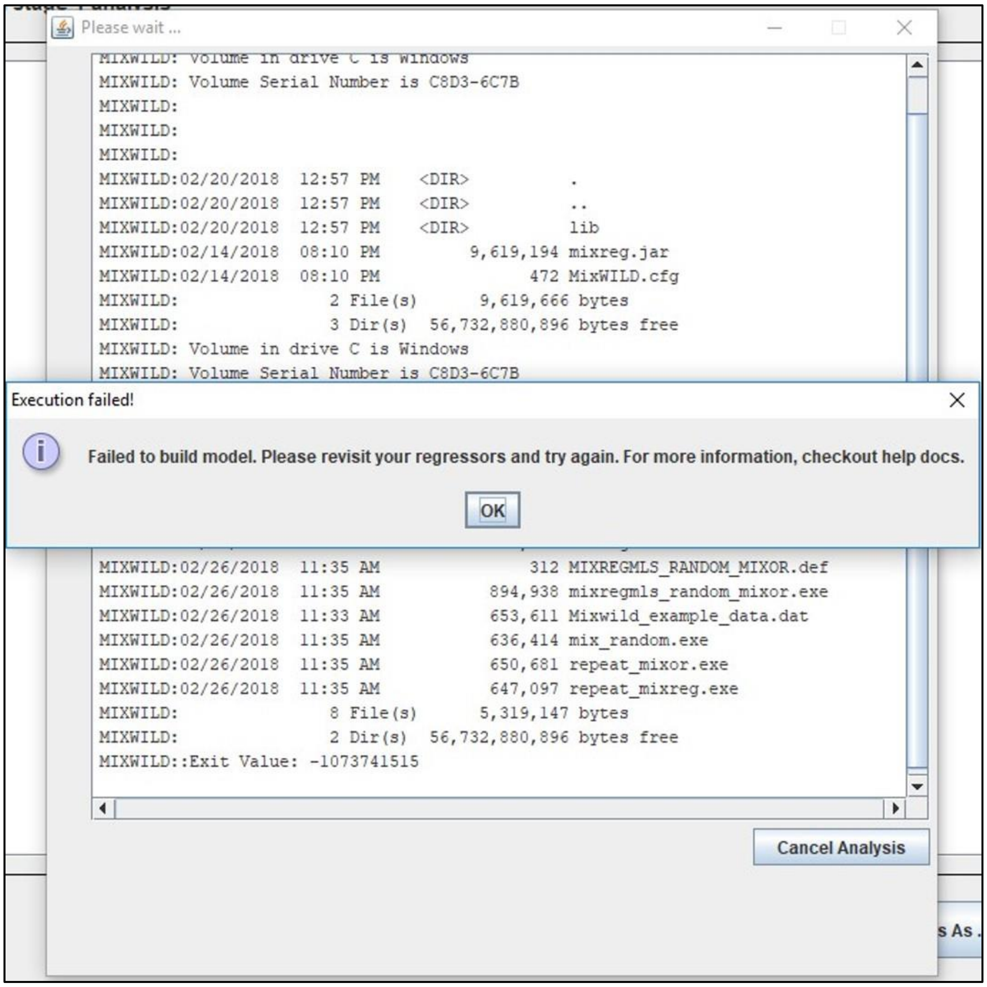

26. When estimation is completed, the Stage 1 and Stage 2 results can be seen by clicking the Stage 1 and Stage 2 Results boxes, respectively.

Stage 1 Configuration | Stage 2 Configuration | Stage 1 Results | Stage 2 Results | View Model | View Data

Results from stage 1 analysis

```
------------------------------------------------------
Model WITH RANDOM Scale and Location-Scale Association
------------------------------------------------------

 ==> multiplied by -2
 Log Likelihood                =    99495.733
 Akaike's Information Criterion =   99515.733
 Schwarz's Bayesian Criterion   =   99566.380


Variable                Estimate    AsymStdError       z-value       p-value
----------------      ------------  ------------  ------------  ------------
BETA (regression coefficients)
Intercept               42.96071       0.50136      85.68797       0.00000
WEEKEND                  1.67701       0.23744       7.06279       0.00000
SEX                     -0.65533       0.56931      -1.15108       0.24970

Random (location) Effect Variances and Covariances
Intercept               71.74435       3.91378      18.33122       0.00000
Covariance12            -9.81601       2.35683      -4.16493       0.00003
WEEKEND                 14.26338       2.57335       5.54273       0.00000

TAU (WS variance parameters: log-linear model)
Intercept                4.71828       0.01968     239.78505       0.00000

Random location effects on WS variance (log-linear model)
Intercept               -0.12884       0.02304      -5.59199       0.00000
WEEKEND                 -0.03716       0.03530      -1.05270       0.29248

Random scale standard deviation
Std Dev                  0.40514       0.02103      19.26143       0.00000
```

Stage 1 Configuration | Stage 2 Configuration | Stage 1 Results | Stage 2 Results | View Model | View Data

Results from stage 2 analysis

```
 Number of successful replications =          500

-------------
Final Results
-------------
 Average Log Likelihood         =    -633.691 (sd= 4.049)
 Akaike's Information Criterion =    -643.691
 Schwarz's Bayesian Criterion   =    -669.015

 ==> multiplied by -2
 Log Likelihood                 =    1267.382
 Akaike's Information Criterion =    1287.382
 Schwarz's Bayesian Criterion   =    1338.030


Variable                Estimate    AsymStdError       z-value       p-value
----------------      ------------  ------------  ------------  ------------
Intercept               -0.23204       0.12897      -1.79908       0.07201
Age                      0.05102       0.00865       5.89725       0.00000
Locat_1                 -0.26222       0.10074      -2.60300       0.00924
Locat_1*Age              0.00489       0.00611       0.80068       0.42332
Locat_2                 -0.03530       0.13734      -0.25703       0.79716
Locat_2*Age              0.00409       0.01000       0.40871       0.68275
Scale                    0.15985       0.12141       1.31670       0.18794
Scale*Age                0.02447       0.00788       3.10482       0.00190
Locat_1*Scale            0.16109       0.14936       1.07849       0.28082
L*S*Age                 -0.00006       0.00943      -0.00608       0.99515
```

27. All files generated from the program can be found in a folder with the prefix MixWILD under the same directory of your dataset.

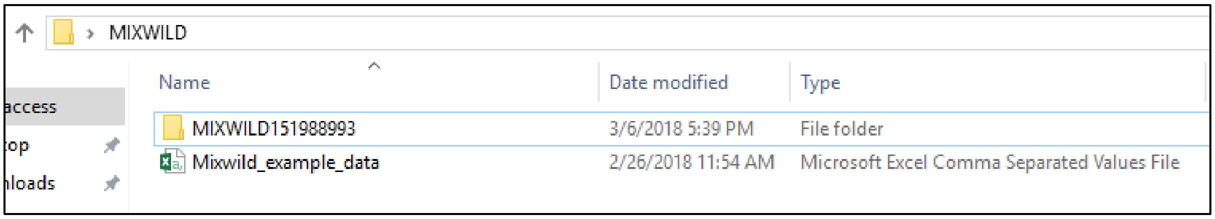

28. The OUT files with suffix_1 and _2 are the results that are identical to those in the Stage 1 and Stage 2 boxes.

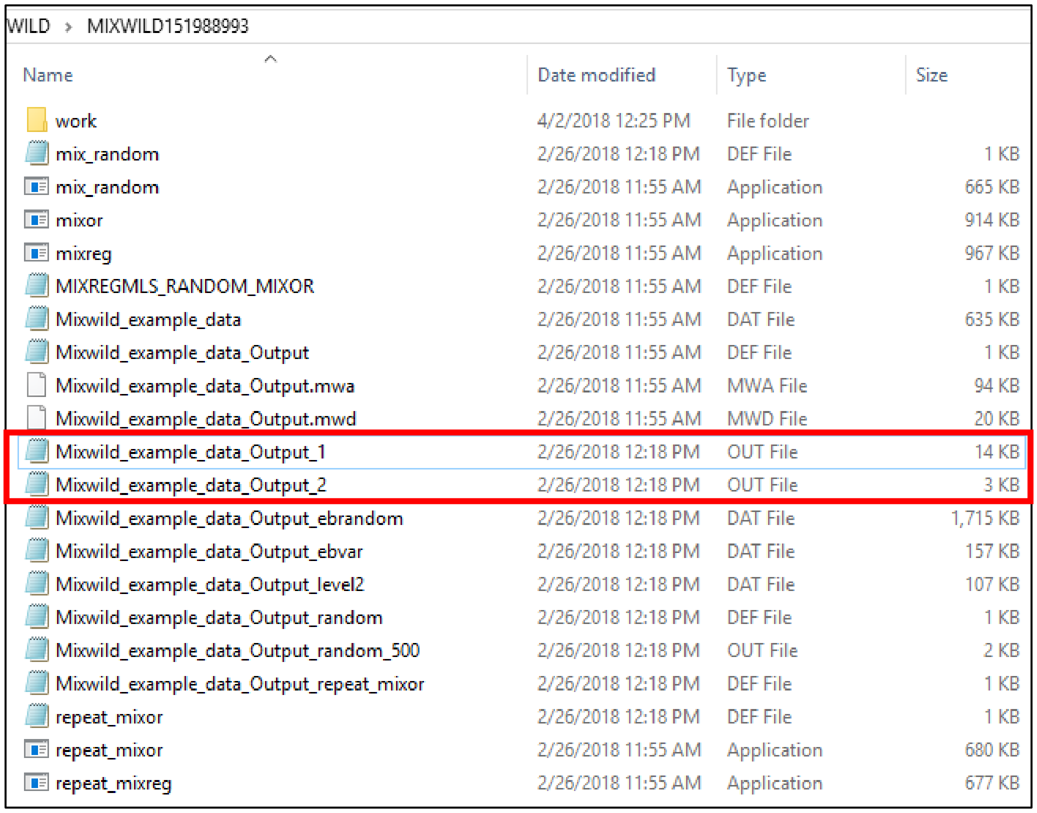

29. There are 3 submodel results in Output_1. The first two submodels present the results with and without random scale estimates, and the third submodel include the estimation of random location effects as well as the random location-slope associations as follows. (A brief description of the results from the following third submodel will be provided)

```
Model WITH RANDOM Scale and Location-Scale Association

 Total  Iterations =  16
 Final Ridge value = 0.00

 Log Likelihood                 =    -49747.866
 Akaike's Information Criterion =    -49757.866
 Schwarz's Bayesian Criterion   =    -49783.190
```

6.3. Brief interpretation of Mixregmls-Logistic regression model result

Stage 1 model with random scale and location-scale association

```
----------------------------------------------------------
Model WITH RANDOM Scale and Location-Scale Association
----------------------------------------------------------

Variable                 Estimate    AsymStdError       z-value       p-value
----------------       ------------  ------------  ------------  ------------
BETA (regression coefficients)
Intercept                42.96071       0.50136      85.68797       0.00000
WEEKEND                   1.67701       0.23744       7.06279       0.00000
SEX                      -0.65533       0.56931      -1.15108       0.24970

Random (location) Effect Variances and Covariances
Intercept                71.74435       3.91378      18.33122       0.00000
Covariance12             -9.81601       2.35683      -4.16493       0.00003
WEEKEND                  14.26338       2.57335       5.54273       0.00000

TAU (WS variance parameters: log-linear model)
Intercept                 4.71828       0.01968     239.78505       0.00000

Random location effects on WS variance (log-linear model)
Intercept                -0.12884       0.02304      -5.59199       0.00000
WEEKEND                  -0.03716       0.03530      -1.05270       0.29248

Random scale standard deviation
Std Dev                   0.40514       0.02103      19.26143       0.00000
```

- The Stage 1 model shows that weekend is positively associated with positive affect (estimate=1.6489, *p*<.001). Individuals overall have higher mean positive affect on weekend days relative to weekdays.
- There is significant variability in both intercept (i.e., random intercept) and slope (i.e., random slope) across subjects. The random intercept is estimated as 71.7444 on the log scale (*p*<.001) and the random slope is estimated as 14.2634 on the log scale (*p*<.001). Therefore, individuals differ from each other in their mean levels of positive affect and in their associations between weekday/weekend and momentary positive affect.
- The random intercept and random slope are negatively associated with each other (estimate= -9.8160, *p*<.001). This negative covariance indicates that subjects with higher weekday mean levels of positive affect (i.e., higher levels of the intercept) do not increase in positive affect as much on weekends, relative to subjects with lower weekday positive affect.
- For the relationships between the random location and scale effects, the random intercept (i.e., weekday positive affect) is negatively associated with the WS variance

(estimated as -0.1288 on the log scale (p<.001). This indicates that subjects with higher weekday positive affect are more consistent/less erratic in their mood reports. A subject's random slope (positive affect change on weekend days relative to weekdays) is not significantly related to a subject's WS variance.

- There is significant variability in scale across subjects, as the Std Dev for the Random scale is estimated as 0.4051 on the log scale (p<.001). Thus, individuals differ from each other in their degree of within-subject/intraindividual variability in positive affect.

Stage 2 model with dichotomous subject-level outcome

```
-------------
Final Results
-------------
 Average Log Likelihood         =     -633.691 (sd= 4.049)
 Akaike's Information Criterion =     -643.691
 Schwarz's Bayesian Criterion   =     -669.015

 ==> multiplied by -2
 Log Likelihood                 =     1267.382
 Akaike's Information Criterion =     1287.382
 Schwarz's Bayesian Criterion   =     1338.030
```

Variable	Estimate	AsymStdError	z-value	p-value
Intercept	-0.23204	0.12897	-1.79908	0.07201
Age	0.05102	0.00865	5.89725	0.00000
Locat_1	-0.26222	0.10074	-2.60300	0.00924
Locat_1*Age	0.00489	0.00611	0.80068	0.42332
Locat_2	-0.03530	0.13734	-0.25703	0.79716
Locat_2*Age	0.00409	0.01000	0.40871	0.68275
Scale	0.15985	0.12141	1.31670	0.18794
Scale*Age	0.02447	0.00788	3.10482	0.00190
Locat_1*Scale	0.16109	0.14936	1.07849	0.28082
L*S*Age	-0.00006	0.00943	-0.00608	0.99515

- In the Stage 2 final results table, *Locat_1* refers to the effect of the random intercept (i.e., within-subject mean) on obesity risk; *Locat_2* refers to the effect of the random slope (i.e., within-subject association between weekday/weekend and positive affect) on obesity risk*; Scale* refers to the effect of random scale (i.e., within-subject variance) on obesity risk; and *Locat_1* Scale* is the interaction between random intercept and random scale predicting obesity risk.
- Results show that after controlling for other variables, subjects' Age is positively associated with increased obesity risk (estimate OR= 0.0510, p<.001). Older subjects are more likely to be obese than younger subjects.
- The random intercept (*Locat_1*) positively predicts obesity risk when the random slope and random slope are zero (estimate OR= -0.2622, p<.001). Since the random effects are centered around zero, a random scale of zero represents the average scale. For subjects with average scale of positive affect, higher levels of weekday positive affect are associated with higher daily MVPA minutes.
- The interaction between Age and random scale is significant in predicting obesity risk in this model (estimate OR= -0.0245, p<.001). The positive association of Age on obesity risk is more pronounced for subjects that are more erratic/less stable in

their positive affect scores. Subjects who are older and who have higher variability in positive affect are more likely to be obese.

- The random slope and other 2-way and 3-way interactions do not predict people's obesity risk in this model.

Note:

1. The numbers of level 1 and level 2 observations correspond to the available non-missing observations of level 1 and level 2 variables included in both Stage 1 and Stage 2 models. Thus, these analyses are carried out using observations that are non-missing in terms of both outcomes (Positive affect and Obesity) and all regressors (Weekend, Sex, and Age).
2. In terms of level-1 variables (Positive affect and Weekend), only the level-1 missing observations are removed. In terms of the level-2 variables (Obesity, Sex, and Age), if a given subject is missing any of these, then they are not included in the analyses.
3. Please refer to the supplemental documents for more detailed explanation of the Stage 1 and Stage 2 model results.

7. Appendix A: Steps to take when the program does not converge to a solution

MixWILD can be used to estimate some rather advanced and complicated statistical models. In some cases, the program does not converge to a solution. This can be because the data does not contain enough information to estimate all of the parameters of the specified model. In this case, trying to estimate a simpler model is warranted. In general, it is good practice to start simply and build model complexity incrementally. In this way, users can get a feel for which variables and/or options may be causing trouble in estimation.

In addition to simplifying the model that one is attempting to estimate, the MixWILD program does include some options that can sometimes be modified to help in estimation of the model parameters. These are included on the Options page and will be described below.

1. This first comment pertains to models that include random scale parameters. Random scale parameters allow subjects to have individual estimates of the within-subject variance, and this is the distinguishing feature of a **mixed-effects location scale model**. The possibility of random scale parameters is specified on the first page that is encountered in the MixWILD program under the **"Random Scale?"** selection. In the graphic below, this is not specified, but clicking on the box to the right of **"Random Scale?"** adds random scale parameters to the Stage 1 model.

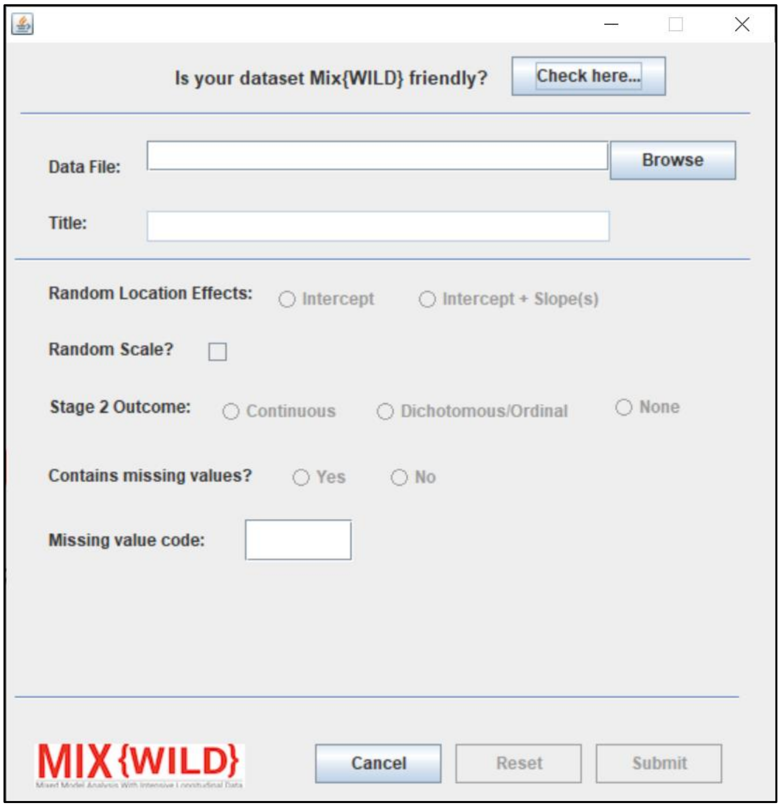

In some cases, there may be subjects in the dataset that exhibit no variation on the Stage 1 outcome variable. In other words, their values on the outcome variable are exactly the same for all of their observations. This can be particularly true if there are subjects with few observations (say 2 or 3) in the dataset and/or the Stage 1 outcome variable is not really continuous but an ordinal outcome with say 5 categories. For such subjects with no variation on the outcome, the estimate of their random scale goes to negative infinity and can cause the program to fail to converge. In this case, selection of the **"Discard Subjects with no Variance?"** option (clicking on the box to the right of this question) on the Options page can facilitate model convergence. Selection of this option will remove these subjects from the Stage 1 analysis (and also the Stage 2 analysis if that is specified).

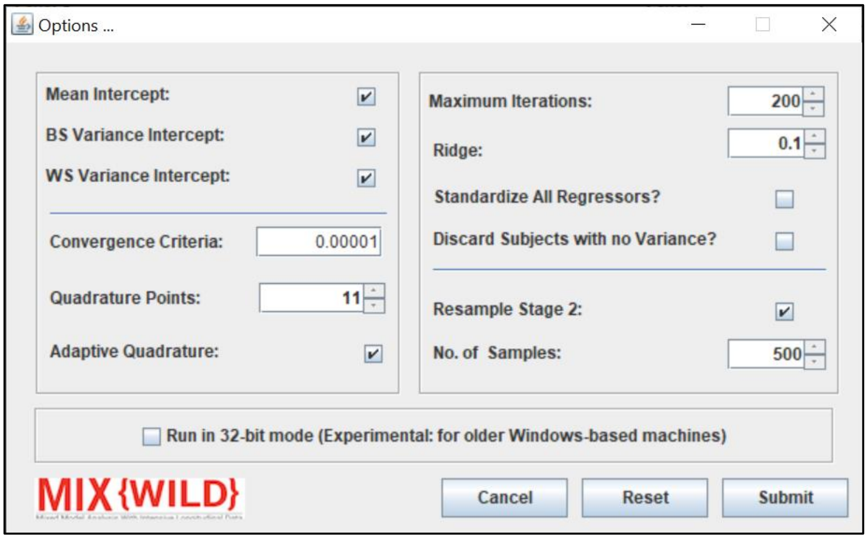

2. In some cases, the scale of the regressors included in an analysis can be very large. For example, consider a scenario in which one has a variable for days in the study, which ranges from 1 to 365 for a year-long study. In this case, because of the large scale of the variable, any parameter associated with this variable will be quite small and can be difficult to estimate. In such cases, standardizing the regressors by selecting "Standardize All Regressors" might be considered. Selection of this option will transform all regressors to be variables with 0 mean and variance of 1.

3. Estimation of the Stage 1 model involves a numerical integration (i.e., quadrature) over the random effect distribution. By default, MixWILD performs adaptive quadrature with 11 points to do this integration. The more points one uses, typically the more accurate is the solution, but also the more time it takes to estimate the model parameters. Usually, 11 points is sufficient, but if model convergence is not achieved, then increasing the points can sometimes help. So, for example, one might try 15, 21, or 25 points rather than the default of 11. Switching from adaptive to non-adaptive quadrature can sometimes work, though typically adaptive quadrature is preferred.

4. The value of the “Ridge” is usually set to a small fractional value. The ridge increases the values of the diagonal elements of the 2nd derivative matrix by a factor of 1 multiplied by the ridge value (i.e., if the ridge is set to .1, then the diagonal elements of the 2nd derivative matrix are all multiplied by 1.1). The reason that this is helpful is that this matrix must be inverted at each iteration of the solution, and inversion of this matrix becomes computationally difficult to the extent that the off-diagonal elements of this matrix get large, relative to the diagonal elements. Thus, in cases of non-convergence, one might try increasing the ridge value to 0.15, 0.2, or even 0.25. This will slow down the estimation, but in some cases can aid in model convergence.

Ultimately, if one tries these options and still experiences convergence problems, it is probably the case that the data does not contain enough information to estimate the specified model. Again, it is a good idea to start simply and to build model complexity in an incremental manner. For example, one might start with a model that does not include any regressors (i.e., a null model) and then to add in regressors one by one or in sets of variables. This will help to isolate where problems develop and to indicate what is and what is not possible with a given dataset.
